# De novo strategy with engineering anti-Kasha/Kasha fluorophores enables reliable ratiometric quantification of biomolecules

**DOI:** 10.1038/s41467-020-14615-3

**Published:** 2020-02-07

**Authors:** Limin Shi, Chenxu Yan, Zhiqian Guo, Weijie Chi, Jingle Wei, Weimin Liu, Xiaogang Liu, He Tian, Wei-Hong Zhu

**Affiliations:** 1 Key Laboratory for Advanced Materials and Joint International Research Laboratory of Precision Chemistry and Molecular Engineering, Feringa Nobel Prize Scientist Joint Research Center, Institute of Fine Chemicals, School of Chemistry and Molecular Engineering, East China University of Science and Technology, Shanghai, 200237 China; 2 Science and Math Cluster, Singapore University of Technology and Design, 8 Somapah Road, Singapore, 487372 Singapore; 3 School of Physical Science and Technology, ShanghaiTech University, Shanghai, 201210 China

**Keywords:** Fluorescent probes, Optical materials

## Abstract

Fluorescence-based technologies have revolutionized in vivo monitoring of biomolecules. However, significant technical hurdles in both probe chemistry and complex cellular environments have limited the accuracy of quantifying these biomolecules. Herein, we report a generalizable engineering strategy for dual-emission anti-Kasha-active fluorophores, which combine an integrated fluorescein with chromene (IFC) building block with donor-π-acceptor structural modification. These fluorophores exhibit an invariant near-infrared Kasha emission from the S_1_ state, while their anti-Kasha emission from the S_2_ state at around 520 nm can be finely regulated via a spirolactone open/closed switch. We introduce bio-recognition moieties to IFC structures, and demonstrate ratiometric quantification of cysteine and glutathione in living cells and animals, using the ratio (S_2_/S_1_) with the S_1_ emission as a reliable internal reference signal. This de novo strategy of tuning anti-Kasha-active properties expands the in vivo ratiometric quantification toolbox for highly accurate analysis in both basic life science research and clinical applications.

## Abstract (web summary)

Fluorescent probes are used in a number of fields but suffer from a lack of quantifiable results due to environmental effects. Here, the authors report on a dual-emission probe which can be used to detect the amount of probe present and the emission from detection applications to allow for quantification.

## Introduction

Fluorescence technologies have revolutionized the way we study life science and conduct medical diagnostics^1–6^. It is now a routine process to directly visualize biomolecules in both fixed and live cells using fluorescence microscopy, which affords high spatial and temporal resolution down to even the single molecule level in a fraction of a second^7–10^. In parallel with the rapid evolution of fluorescent imaging techniques, increased attention is now focused on the accurate quantitative measurement of biomolecules via fluorescent signals^11–14^. However, in a highly heterogeneous and dynamic biological milieu, obtaining quantitative information of biomolecules remains a significant challenge^15^: The emission intensities of fluorescent probes are affected by both the target biomolecules and other extraneous factors such as probe concentrations, excitation power, and cellular context.

**Fig. 1:**
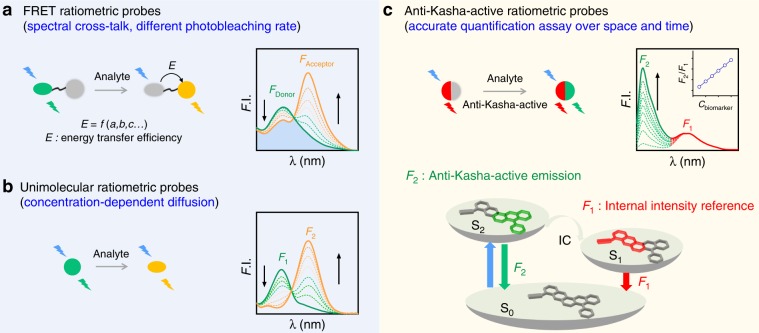
Strategies for ratiometric probes with characteristic analysis. **a** FRET ratiometric probes: excitation/emission cross-talk. **b** Unimolecular ratiometric probes: different photobleaching rates and concentration-dependent diffusion between reactant and product fluorophores in dynamic cellular environment. **c** In this work, anti-Kasha-active ratiometric probes enabling accurate and reliable ratiometric quantification: an invariant NIR emission from the Kasha-based S_1_ state serve as an internal reference; a green emission from the anti-Kasha-active S_2_ state linearly increase with the concentration of targeted analyte, due to the rapid radiative process from S_2_ to S_0_ state, competing with the internal conversion (IC) from S_2_ to S_1_ state.

To eliminate the impact of extraneous factors, one solution is to employ two fluorophores with equal or correlated concentrations to ratiometrically quantify biomolecules. To this end, several techniques have been developed, such as Fӧrster resonance energy transfer (FRET) and unimolecular based ratiometric probes (Fig. 1a, b). The most prominent technology behind these methods is the FRET mechanism, which uses a single excitation source but acquires two emission bands from a set of paired reporter fluorophores (Fig. 1a)^16^. Monitoring the ratiometric changes in the emission intensity of these two paired fluorophores enables the quantification of a target analyte^17^. Despite its undeniably huge impact in research, many issues still complicate FRET measurements, some of which can cause misleading or even render meaningless results, for example inevitable excitation/emission cross-talk^18^, conformational and/or microenvironmental changes between the energy donor and acceptor fluorophores^19^, and different photobleaching efficiencies of the two fluorophores. Thus, FRET requires sophisticated calibrations to generate reliable signals.

It has been established that the preferential ratiometric patterns of a unimolecular fluorophore can overcome some inevitable side effects between donor and acceptor fluorophores in FRET-based methods^20–23^. Unimolecular ratiometric probes can react with the analyte to form a new fluorophore. The emission intensity ratios between the reactant and product fluorophores—which are typically excited at two distinct wavelengths—can enable quantification of the analyte (Fig. 1b). Nevertheless, in dynamic cellular environment, different photobleaching rates and concentration-dependent diffusion properties of the reactant and the product fluorophores have limited the widespread applications of such unimolecular probes. Moreover, it is challenging for unimolecular probes to establish a suitable and reliable internal intensity reference to enable accurate quantitative measurements^24,25^.

One effective approach to address these challenges would be to develop a single-type of probe with dual-emission signals: one serving as the internal intensity reference and the other for representing interactions with the target analyte (Fig. 1c). Unfortunately, such probes are often deemed impossible in that they defy Kasha’s rule. As an important principle in photochemistry to most fluorophores, Kasha’s rule states that fluorophores can only generate one emission band from the lowest-energy excited singlet state (S_1_), irrespective of the excitation wavelength^26^. In contrast, anti-Kasha dyes remain exceedingly rare^27–32^, and most of them emit in the short wavelength region and are thus not suitable for biological applications^33^. Moreover, to the best of our knowledge, the modulation of anti-Kasha emission for ratiometrically detecting bioanalytes has yet to be demonstrated.

Herein, we report a general strategy to construct activatable anti-Kasha dual-emission fluorophores based on a discovered building block, which we coin as integrated fluorescein with chromene (IFC) chromophores, wherein a spirolactone open/closed switch can finely regulate anti-Kasha-active behavior. Using the designed probes such as dicyanomethylene-4H-pyran (DCM)-IFC, we were able to conduct in vivo measurement and quantification of biomolecules at the physiological pH condition. The near-infrared (NIR) Kasha emission signal (700 nm) from S_1_ state of DCM-IFC remains nearly constant, while its anti-Kasha emission signal from S_2_ state increases linearly with analyte concentrations via a spirolactone open/closed switch. The ratiometric bioimaging based on the single fluorophore allows us to accurately perform quantification of various biomolecules in living cells and a mice model. This de novo strategy for activatable anti-Kasha fluorophores would greatly facilitate the advancement of quantitative fluorescence imaging.

## Results

### Revealing the anti-Kasha properties of open-form DCM-IFC

**Fig. 2:**
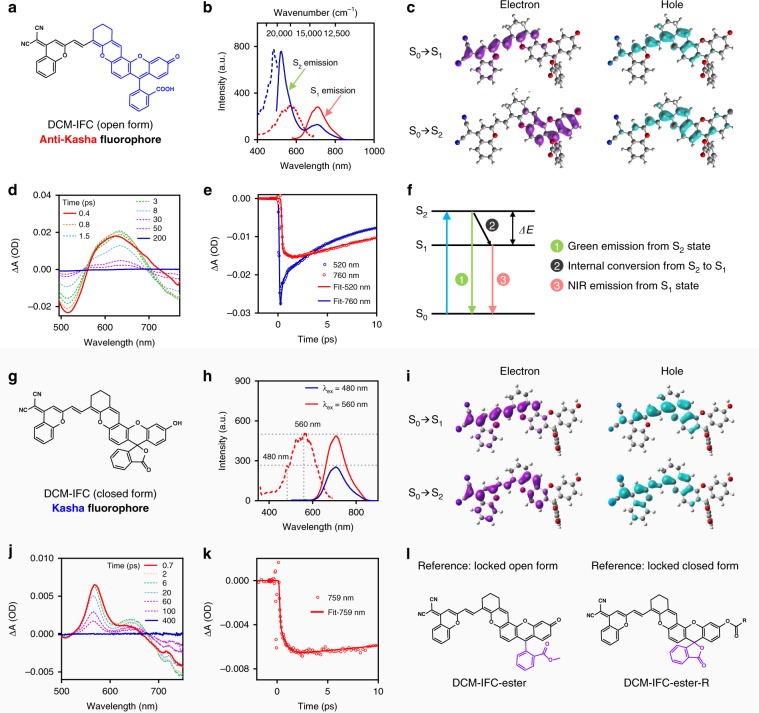
Characterization of anti-Kasha mechanism in open-form versus Kasha mechanism in closed-form. **a** Chemical structure of DCM-IFC in its open form. **b** Excitation spectra (dotted line, monitored at *λ*_em_ = 520 and 710 nm) and emission spectra (solid line, excited at *λ*_ex_ = 480 and 560 nm) of open-form DCM-IFC. **c** Electron–hole analysis involved during the photoexcitation of the open form, based on the molecular structure at the Franck–Condon state. Femtosecond time-resolved transient absorption spectra (**d**) and kinetics (**e**) of open-form DCM-IFC (excited at *λ*_ex_ = 480 nm). **f** Jablonski diagram illustrating the anti-Kasha mechanism. **g** Chemical structure of DCM-IFC in its closed form. **h** Excitation spectra (dotted line, monitored at *λ*_em_ = 710 nm) and emission spectra (solid line, excited at *λ*_ex_ = 480 and 560 nm) of closed-form DCM-IFC. **i** Electron–hole analysis involved during the photoexcitation of the closed form, based on the molecular structure at the Franck–Condon state. Femtosecond time-resolved transient absorption spectra (**j**) and kinetics (**k**) of closed-form DCM-IFC (excited at *λ*_ex_ = 480 nm). Note: open-form DCM-IFC was investigated at pH 11.3 and closed-form DCM-IFC was investigated at pH 2.0. **l** Two structurally related reference compounds that are locked into open form or closed form. Note: all fluorescence spectra were measured in a mixture solution of acetonitrile (MeCN)/Britton–Robinson buffer.

Our group has been engaged in a long-term research project investigating the use of high-performance donor-π-acceptor (D-π-A) fluorescent dyes^34,35^, such as DCM, QM, and BODIPY, aiming to expand the utility of such fluorophores along with tailoring their emission properties for high-fidelity bioimaging in vivo. Among these conjugated molecules (Supplementary Figs. 1 and 2), the DCM-IFC fluorophores (covalently integrated fluorescein with chromene) immediately grabbed and focused our attention when we discovered its extremely unusual dual-emission. Upon excitation at 480 nm, DCM-IFC (open form, Fig. 2a) displayed two emission peaks at 520 and 700 nm, respectively. When the excitation wavelength was shifted to 560 nm, only the NIR peak at 700 nm was observed (Fig. 2b). We verified that this unusual dual-emission did not result from contaminations or molecular aggregations by conducting several solvent-dependent and concentration-dependent studies (Supplementary Fig. 3). Clearly, the excitation-dependent emission in DCM-IFC (open form) is not in agreement with Kasha’s rule^36^ and warranted further investigation.

We subsequently carried out the experiments with excitation and emission spectra to validate the anti-Kasha mechanism in the open-form DCM-IFC. The fluorescence spectra revealed two emission bands that corresponded to different excitation peaks at 480 nm and 560 nm, respectively (Fig. 2b). Each excitation peak was mirror-image of their corresponding emission peak, which led us to hypothesize that the two emission bands originated from two different excited states in the open-form DCM-IFC.

Furthermore, quantum chemical calculations were conducted, and their results strongly corroborate the experimental data: the two emission bands matched the fluorescence from the S_1_ (NIR) and S_2_ (visible) states, respectively (Supplementary Table 1 and Supplementary Fig. 4). Electron–hole analysis also indicated that the electron of the S_2_ state has a considerable distribution in the fluorescein moiety, while the electron of the S_1_ state mainly localizes in the DCM fragment (Fig. 2c). Notably, the two excited states are characterized by a large energy gap: Δ*E*(S_2_−S_1_) is 0.74 eV (around 6000 cm^−1^, Supplementary Table 1). This large gap can effectively suppress the internal conversion (IC) from S_2_ to S_1_ state. Moreover, our calculations indicate that the radiative decay (to S_0_) from S_2_ (6.59 × 10^8^ S^−1^) is approximately three times faster than that of from S_1_ (1.96 × 10^8^ S^−1^). Collectively, these computational results help elucidate the anti-Kasha characteristic which was observed for open-form DCM-IFC.

The anti-Kasha characteristic with dual-emission was also strongly supported by femtosecond time-resolved transient absorption spectroscopic experiments. The time-resolved experiments showed two stimulated emission bands, which is in agreement with our initial hypothesis that the dual-emission stemmed from two discrete excited states (Fig. 2d). The measurement clearly denotes an energy transfer process (IC) from S_2_ to S_1_ state with a lifetime of ~1 ps (Fig. 2e and Supplementary Table 2); the S_2_ fluorescence lifetime is also short (45 ps at 520 nm). According to a general rule^26^, the IC process takes place at least 10^4^ times as fast as the spontaneous S_n_ emission. However, DCM-IFC displayed a rapid radiative process from S_2_ to S_0_ state, which implies that the rate of S_2_ emission decay can compete with IC from S_2_ to S_1_ state. These ultra-fast experimental insights led us to propose an intrinsic mechanism of anti-Kasha with dual-emission: a comparable rate between the radiation decay process from S_2_ state and IC to the S_1_ state can account for the observed dual-emission of our anti-Kasha chromophore DCM-IFC (Fig. 2f).

### Engineering anti-Kasha/Kasha fluorophores

Although open-form DCM-IFC displayed dual-emission with anti-Kasha properties, closed-form DCM-IFC (Fig. 2g) exhibited only a single-emission band at 700 nm (Fig. 2h). Fluorescence spectra, transient absorption spectra, and quantum chemical calculations (Fig. 2h–k) indicated that closed-form DCM-IFC had emission properties that obeyed Kasha's rule. To further support the discrete properties of open- versus closed-form DCM-IFC, two reference compounds were specifically designed and subsequently synthesized: one compound was structurally locked in an open form, while the other was locked in a closed form. Accordingly, the corresponding reference DCM-IFC-ester clearly exhibited two emission peaks at 520 and 700 nm in dichloromethane, which strongly confirms the anti-Kasha property to the open form of DCM-IFCs (Fig. 2l, Supplementary Figs. 5–7 and Supplementary Tables 3–4). In contrast, DCM-IFC-ester-R demonstrated the typical single-emission Kasha property (Supplementary Fig. 8). Taken together, these related IFCs strongly confirm the anti-Kasha/Kasha properties in the open/closed forms of DCM-IFCs.

**Fig. 3:**
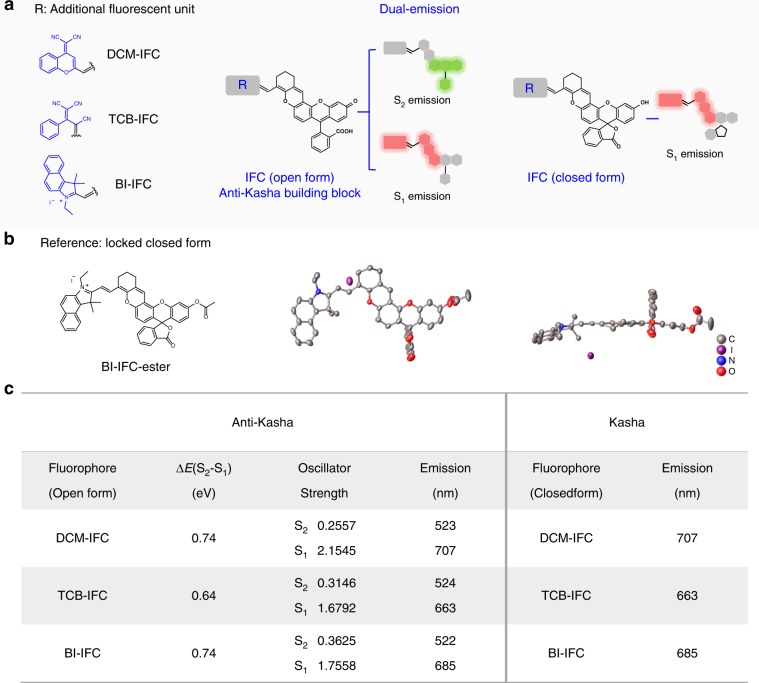
A generalizable molecule engineering strategy for developing anti-Kasha/Kasha fluorophores. **a** Schematic illustration of designing anti-Kasha/Kasha fluorophores. **b** Molecular and crystal structures of BI-IFC-ester (locked closed form). **c** Quantum chemical calculations and experimental data of anti-Kasha/Kasha fluorophores. Δ*E*(S_2_−S_1_) and oscillator strength were obtained from quantum chemical calculations. Emission was obtained from experimental data.

Starting from this initial DCM-IFC molecule, a series of fluorophores were developed based on simple structural modifications using the following guidelines: (i) start with IFC (integrated fluorescein with chromene) backbone as a potential anti-Kasha building block; (ii) introduce an additional electron-withdrawing fluorescence unit (like DCM, TCB, or BI) for extending Δ*E*(S_2_-S_1_) (Fig. 3a). To our delight, the open forms of all resulting fluorophores displayed anti-Kasha dual-emission (S_2_ emission: 520 nm; S_1_ emission: 663 to 707 nm; Fig. 3c and Supplementary Table 5), whereas their closed forms exhibited normal single-emission (S_1_ emission), complying with Kasha’s rule (Fig. 3c).

Subsequent theoretical quantum calculations indicated that the electron distributions for the S_2_ state of these fluorophores (open form) mainly reside in their fluorescein moieties, whereas in the S_1_ state, the electrons are localized to their electron-withdrawing fluorescence units (e.g., TCB, BI; Supplementary Figs. 9 and 10). Moreover, all these fluorophores have large gap Δ*E*(S_2_−S_1_) (Fig. 3c), which is in agreement with a previous report for anti-Kasha fluorophores with substantial S_2_ emission^37^. Furthermore, such IFC-integrated fluorophores in their closed forms demonstrated the typical single-emission Kasha band. Fortunately, we acquired a single crystal of reference compound BI-IFC-ester which was absolutely locked in a closed form (Fig. 3b). According to crystal analyses, the conjugated fluorophore combined IFC building block with BI unit exhibits a large coplanar conformation, which is responsible for NIR emission. Compiling these results together, we have successfully developed a generalizable strategy with molecular engineering to extend Δ*E*(S_2_−S_1_) gaps that enables construction of diverse anti-Kasha/Kasha chromophores.

### Modulating anti-Kasha/Kasha emission for ratiometric mode

**Fig. 4:**
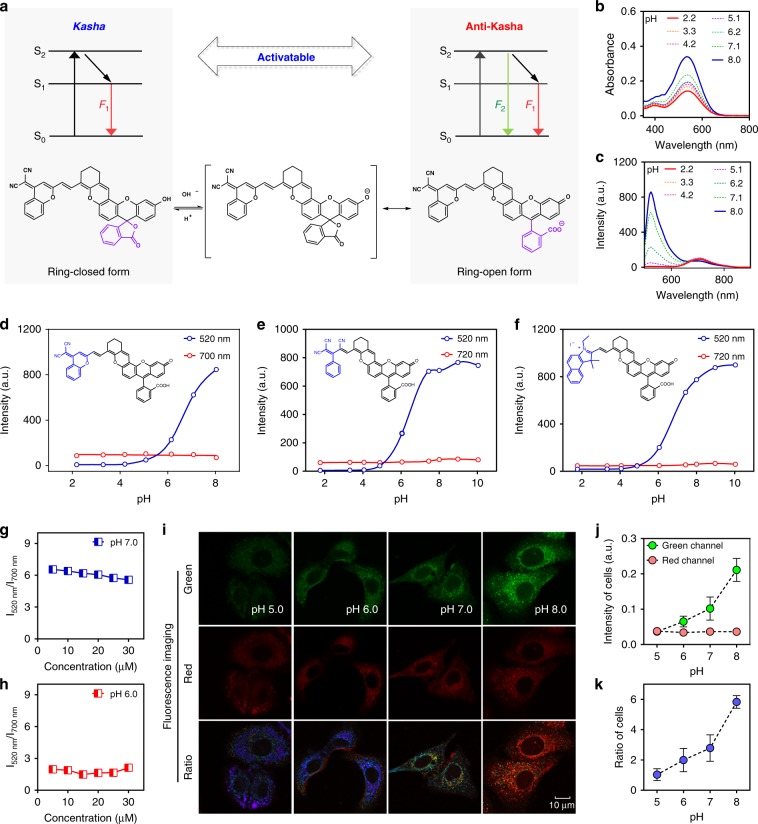
Anti-Kasha/Kasha switch-induced dual-emission response with an internal intensity reference signal. **a** Schematic illustration of spirolactone-switch-controlled molecules for tuning anti-Kasha/Kasha properties. Absorption (**b**) and fluorescence spectra (**c**) of DCM-IFC in the range of pH 2.2–8.0. Dual-emission response with the internal intensity reference signal of DCM-IFC (**d**), TCB-IFC (**e**), and BI-IFC (**f**) at different pH values, *λ*_ex_ = 480 nm. Source data are provided as a Source Data file. Concentration-dependent emission ratios (S_2_/S_1_) detected for DMC-IFC at pH 7.0 (**g**) and pH 6.0 (**h**). Source data are provided as a Source Data file. Dual-channel ratiometric imaging (**i**), fluorescence intensity (**j**) and ratio (**k**) of DCM-IFC in A549 cells across different pH values. Data with error bars are expressed as mean ± s.d., *n* = 3. Source data are provided as a Source Data file. Note: all fluorescence spectra were measured in a mixture solution of MeCN/Britton-Robinson buffer.

To date, it remains very challenging to rationally evoke/regulate emission from higher excited states based on anti-Kasha fluorophores^38^, and these difficulties severely hinder the applicability of such molecules for optical imaging and biosensing. Given that the closed-form DCM-IFC (Kasha fluorophore) and the open-form DCM-IFC (anti-Kasha fluorophore) can be reversibly interchanged via the opening and closing of the spirolactone ring (achieved by manipulating pH, Fig. 4a), we hypothesized that rational modulation of the S_2_ emission could be achieved via anti-Kasha/Kasha switch.

To verify our hypothesis, we investigated the effect of pH on the spirolactone ring for the anti-Kasha/Kasha switching of DCM-IFC (Fig. 4b, c). It can be rationalized that a low pH environment would induce a spirocyclization reaction that causes the IFC moiety to adopt its closed form. Conversely, increasing pH values would promote deprotonation and change the IFC moiety into its open form. The solvent pH was increased from 2.2 to 8.0 and the spirolactone ring-opening was monitored (from Kasha to anti-Kasha): The S_2_ emission at 520 nm progressively intensifies from an initial zero background. Here the S_2_ emission response clearly established that rational modulation of emission from higher excited states can be achieved by tuning anti-Kasha/Kasha properties.

To our surprise, the intensity of the S_1_ emission at 700 nm remains invariable under the tested range of pH values, which suggests that the Kasha/anti-Kasha dynamics may not influence the S_1_ emission of DCM-IFC. This response can be explained by quantum chemical calculations and absorbance spectra: (i) calculations showed that the electron density (S_1_ state) of the open form and closed form displayed remarkable overlap (Fig. 2c, i), (ii) DCM-IFC displayed similar emission oscillator strength in the S_1_ state (*f*_closed_ = 1.9527, *f*_open_ = 2.1545) between the closed and the open form since S_1_ state localizes in the DCM moiety and is relatively unaffected by the spirocyclization of the fluorescein moiety, and (iii) the enhanced emission at 520 nm may originate from DCM-IFC's increased photoreceptivity of the open vs. closed form as the pH changes (Fig. 4b, c).

The combination of variable S_2_ anti-Kasha emission with nearly invariant S_1_ Kasha emission alludes the possibility that our IFCs could be exploited as unimolecular probes for ratiometric imaging and for quantification applications. We envisioned that S_1_ emission could act as an internal intensity reference, while the S_2_ emission would reliably respond and reflect pH changes. As expected, the spirolactone-switch-induced dual-emission response with a constant internal intensity reference was exhibited by all of the IFC-derived chromophores (Fig. 4d–f and Supplementary Figs. 11 and 12). In addition, the ratio (S_2_/S_1_) of dual-emission remained stable across varying probe concentrations (5–30 μM), indicating that the observable ratio (S_2_/S_1_) of IFC-derived chromophores is completely concentration-independent (Fig. 4g, h). Subsequently, as a proof-of-concept, we investigated the applicability of dual-emission IFC probes for monitoring pH changes in living cells (Fig. 4i–k). Overall, we have successfully developed and demonstrated a viable strategy for ratiometric analysis with an internal intensity reference signal based on modulation of the anti-Kasha/Kasha properties.

### Ratiometric quantitative sensing over space and time

**Fig. 5:**
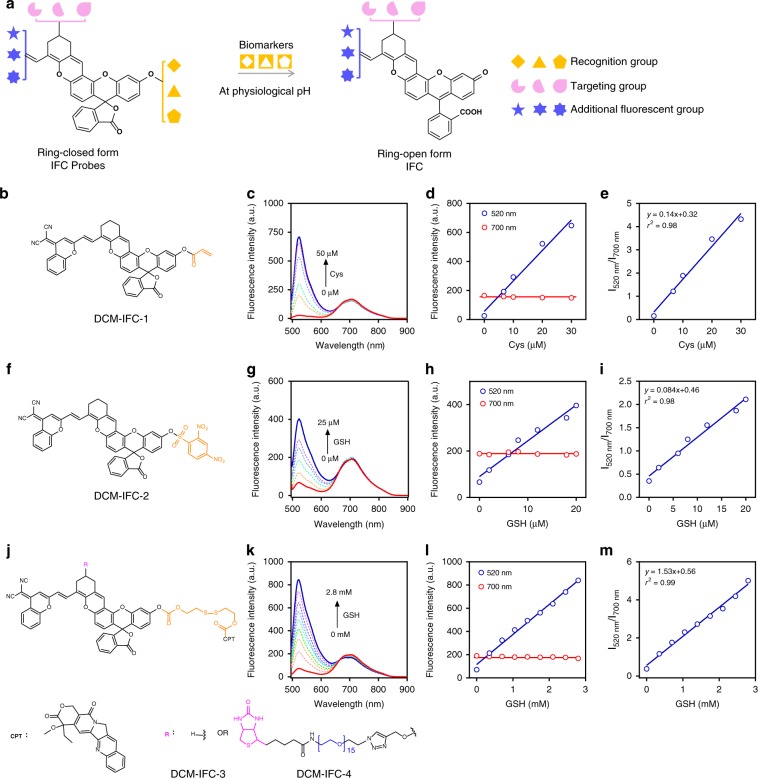
A generalizable method for quantitative analysis with anti-Kasha-active chromophores. **a** Quantitative sensing platform. Chemical structures of DCM-IFC probes with different biomolecular-recognition groups: (**b**) acrylate group towards Cys; (**f**) 2,4-dinitrobenzenesulfonyl towards GSH; (**j**) disulfide bond towards GSH, sense-and-release. Fluorescence spectra (**c**, **g**, **k**), ratiometric response with internal reference signal (**d**, **h**, **l**, Source data are provided as a Source Data file.) and dual-channel linear ratio analysis (**e**, **i**, **m**, Source data are provided as a Source Data file.) of DCM-IFC-1, DCM-IFC-2, DCM-IFC-3, and DCM-IFC-4 for various analytes. Note: all fluorescence spectra were measured in a mixture solution of MeCN/phosphate-buffered saline (PBS, pH = 7.4), *λ*_ex_ = 480 nm.

Given that IFC chromophores mainly remain in their open form, and emit both S_2_ and S_1_ fluorescence signals at the physiological pH range of 7.0–8.0 (Fig. 4c–f), we hypothesized that replacing the hydroxy group in IFCs with different biorecognition units can absolutely lock such probes in their closed form and emit only NIR fluorescence (S_1_). Upon encountering their corresponding analyte, the hydroxyl group would be restored, thereby turning the probe to its open form at the desirable physiological pH, which would emit both green (S_2_) and NIR (S_1_) fluorescence (Fig. 5a). By calculating the ratio of S_2_ and S_1_ emission, it is thus possible to determine the concentration of the target analyte. In this regard, various ratiometric fluorescent probes could be built up via using our specific IFC chromophores.

Consequently, the following functionalized IFC probes were synthesized: DCM-IFC-1 with an acrylate group for sensing cysteine (Cys), DCM-IFC-2 with 2,4-dinitrobenzenesulfonyl for sensing glutathione (GSH)^39,40^, as well as DCM-IFC-3 and DCM-IFC-4 with a disulfide bond that can sense GSH and then release the anticancer drug camptothecin (CPT)^41,42^ (Fig. 5b, f, j). As shown in Fig. 5c, g, k, a ratiometric response with the internal intensity reference signal (linearly increasing S_2_ emission intensity, stable S_1_ emission) was observed in all test assays at desirable physiological pH condition. For example, with probe DCM-IFC-1, the fluorescence signal at 520 nm increased linearly with the concentration of Cys, while the emission at 700 nm remained nearly constant (Fig. 5d). The intensity ratios at these two wavelengths correlated linearly with the concentrations of Cys (Fig. 5e). Similar emission profiles and ratiometric correlations were also observed in DCM-IFC-2, DCM-IFC-3 and DCM-IFC-4 towards GSH (Fig. 5h, i, l, m). These results (Fig. 5 and Supplementary Figs. 13–26) showed that the IFC platform provided a generalizable method for quantitative analysis of various biomolecules via ratiometric imaging using activatable anti-Kasha S_2_ emission and invariant S_1_ emission as a stable reference signal.

**Fig. 6:**
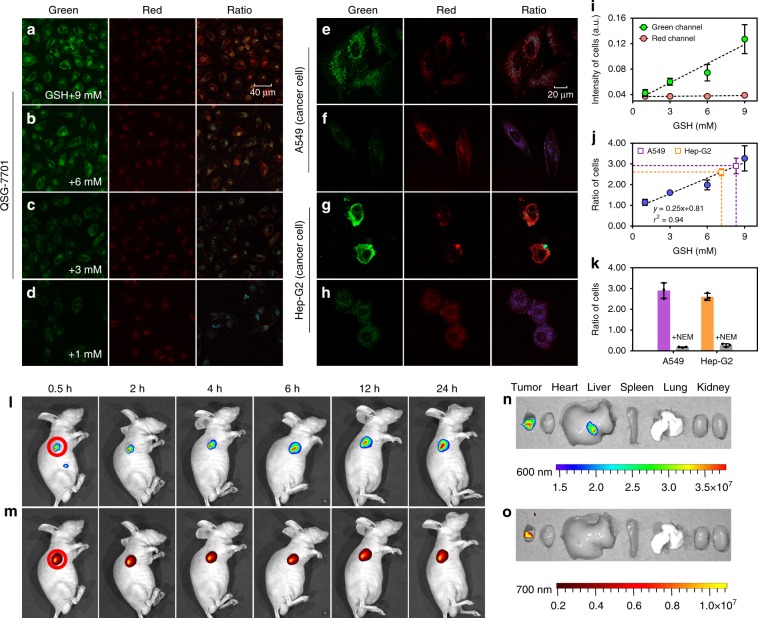
Quantitative sensing in live cells and dual-emission imaging in vivo. Dual-channel and ratiometric imaging of QSG-7701 cells (**a**–**d**, pretreated with various concentrations of GSH); A549 and Hep-G2 cells incubated with DCM-IFC-4 (30 µM) and either untreated (**e**, **g**) or treated with (**f**, **h**) NEM (a derivatization agent which covalently sequesters GSH). Note: The green channel was 520 ± 20 nm, the red channel was 700 ± 20 nm, and ratiometric images were generated from the 520 and 700 nm channels, *λ*_ex_ = 488 nm. **i** Dual-channel response with internal reference signal in cells. Data with error bars are expressed as mean ± s.d., *n* = 3. Source data are provided as a Source Data file. **j** Standard curve of the I_520 nm_: I_700 nm_ ratio as a function of GSH concentration. Data with error bars are expressed as mean ± s.d., n = 3. Source data are provided as a Source Data file. **k** Ratio value in A549 and Hep-G2 cells with and without NEM. Data with error bars are expressed as mean ± s.d., *n* = 3. Source data are provided as a Source Data file. (**l**, **m**) In vivo dual-channel fluorescence imaging of xenograft tumor (A549 cell) bearing mice at various times (0.5, 2, 4, 6, 12, 24 h) after the intravenous injection of DCM-IFC-4 (2.44 mg kg^−1^), the tumor site is circled in red. **n**, **o** Ex vivo dual-channel fluorescence imaging of the excised organs (tumor, heart, liver, spleen, lung, and kidney) at 24 h after the intravenous injection of DCM-IFC-4. Note: fluorescence signals at 600 nm (rainbow scale) and 700 nm (yellow-red scale).

The next step involved the use of our anti-Kasha-active chromophores for quantitative analysis of endogenous biomolecules in living systems. To establish the correlation between the GSH concentration and the ratio (S_2_/S_1_), we pretreated QSG-7701 cells with excessive N-methylmaleimide (NEM, a derivative known to covalently sequester GSH, 500 μM) for 30 min to remove cellular GSH (Fig. 6a–d). After removing the NEM, the pretreated cells were then incubated with different doses of GSH for another 30 min, followed by further incubation in 30 μM of DCM-IFC-4 for 60 min. It is noteworthy that the intensity of the green channel (S_2_ emission, 520 ± 20 nm) gradually increased with the GSH concentration, while that of the red channel (S_1_ emission, 700 ± 20 nm) remained nearly constant (Fig. 6i), which is consistent with previous experiments in vitro (Fig. 5). Moreover, the ratio of resulting fluorescence intensity between the green channel and the red channel demonstrated a strong linear correlation with the GSH concentration (*r*^2^ = 0.94, Fig. 6j). This correlation can be used to generate a calibration curve that enables the precise determination of GSH concentration in cells simply by using reliable ratiometric imaging.

Subsequently, we used DCM-IFC-4 to quantify GSH concentration in GSH-enriched cells from two tumor cell lines^43^ (human lung cancer cells A549 and human hepatic cancer cells Hep-G2, Fig. 6e–h). Under identical experimental conditions, A549 cells and Hep-G2 cells exhibited intensity ratios of 2.90 and 2.59, respectively (Fig. 6j). By extrapolating the calibration curve, we calculated the GSH concentrations in A549 cells (8.36 mM) and in Hep-G2 cells (7.12 mM). These concentrations are in good agreement with previously reported GSH concentration values^44^. Furthermore, upon exposure of both cell lines with NEM, we observed a sharp decrease in the green channel emission and the associated intensity ratio between the green and red channels (Fig. 6f, h, k). These experiments helped to establish how our anti-Kasha-active fluorophores enable quantitative ratiometric analysis of endogenous biomolecules in living cells.

In addition to quantitative analysis of biomolecules in cells, our anti-Kasha IFC platform also realized a "sense-and-release" activity for targeted drug delivery. An ideal targeted drug delivery system should allow accurate pathologic diagnosis of a given cell's state and then precisely deliver said drug at its optimal dosage, thus achieving personalized theragnostics. However, owing to a dearth of reliable quantitative analytical methods, it remains largely difficult for the controlled release of dosages. To address this issue, we designed and synthesized DCM-IFC-4 that contains two functional groups. One of the functional groups is a hydrophilic PEG oligomer-bridged biotin segment, responsible for active targeting of tumor cells^45^. The other is a hydrophobic disulfide-bridged anticancer prodrug CPT (Supplementary Fig. 27). Upon DCM-IFC-4's interaction with GSH, the CPT drug is released. High-performance liquid chromatography (HPLC) experiments showed that the CPT release dosages were linearly dependent on GSH concentrations (Supplementary Fig. 28). These results serve to demonstrate a controlled way of releasing drug dosages based on our designed DCM-IFC motif.

Finally, we explored the use of DCM-IFC-4 for selectively sensing biomolecules and releasing CPT in A549 tumor-bearing mice. After the intravenous injection of DCM-IFC-4 (2.44 mg kg^−1^) to A549 xenograft tumor-bearing mice, in vivo and ex vivo fluorescence bioimaging was performed via a dual read-out channel: 600 ± 20 nm (Fig. 6l) and 700 ± 20 nm (Fig. 6m). The 700 nm emission signal was indicative of specific probe accumulation in tumors, and here the observed specificity can be attributed to the synergistic passive (a preferable micelle-based EPR effect) and active (biotin receptor-mediated endocytosis) targeting abilities. The emission intensity at 600 nm gradually increased throughout the treatment window period. To verify that the fluorescence signals were only caused by probe accumulation in the tumor cells, we recorded fluorescence images of ex vivo tumor tissue and major organs (Fig. 6n, o). Ex vivo data showed that only the tumor tissue displayed a strong fluorescence signal, exactly in good agreement with the in vivo results. These imaging results further highlight the potential of DCM-IFC-4 for in vivo quantification of biomolecules in targeted cell types, as well as for synergistic tumor targeting and controlled drug release strategies.

## Discussion

There are well-known limitations with fluorescence-based technologies for cellular imaging, and especially for the inevitable excitation/emission cross-talk in FRET mode. Another important limitation of the current analytical strategy is the lack of a constant signal for monitoring and measuring biomolecules which can be used as an internal intensity reference. Indeed, this lack is widely recognized among the FRET community, and all manner of workarounds have been proposed and employed to overcome this fundamental deficiency. Some groups have adopted unimolecular probes in their efforts to overcome challenges to accurate quantification in dynamic cellular environment, but still suffer from inherent limitations particularly, including errors from environmental sensitivity and concentration-dependent diffusion properties of dual emission between reactant and product fluorophores.

The development of anti-Kasha chromophores with dual-emission properties and a stable internal intensity reference signal simultaneously addresses major challenges to limited quantitative analysis of biomolecules in dynamic cellular environment. Fundamentally, the essential enabling innovation of our study is aimed at evoking/regulating emission from higher excited states based on anti-Kasha fluorophores, whereas almost all molecules exhibit only single emission according to the so-called Kasha’s rule. In particular, given the exceedingly rare anti-Kasha dyes, it is a long-term goal for scientists that unimolecular fluorophores with discrete excitation states and attendant Kasha vs. anti-Kasha emission, exhibit dual-emission spectra behaviors. Notably, this elaboration of the underlying design principle to generate ratiometric probes can construct and exert the reliable quantitative calibration for various biomolecules, not affected by extraneous factors including probe concentrations, excitation power, and cellular context.

In summary, we focused on engineering long-wavelength anti-Kasha/Kasha fluorophores for reliable ratiometric quantification of biomolecules in a physiological context. We have successfully developed a generalizable strategy with molecular engineering to extend Δ*E*(S_2_−S_1_) gaps that enables construction of diverse anti-Kasha/Kasha chromophores via introducing an additional electron-withdrawing fluorescence unit to integrated fluorescein with chromene (IFC) building block. With the quantum chemical calculations and femtosecond time-resolved transient absorption spectroscopic experiments, the IFC-based fluorophores display a rapid radiative process from S_2_ to S_0_ state, and can compete with the IC from S_2_ to S_1_ state, thereby accounting for the intrinsic anti-Kasha mechanism with dual-emission: an invariant NIR emission from the Kasha-based S_1_ state and a green emission at around 520 nm from the anti-Kasha-based S_2_ state. The combination of the dual-emission and invariant NIR internal reference in these fluorophores allows construction of various probes that can be applied for reliable ratiometric bioimaging and quantitative sensing of biomolecules (such as GSH and Cys) at the desirable physiological pH range of 7.0-8.0, as well as monitoring targeted drug release, both in vitro and in vivo.

Our work represents a ratiometric sensing platform based on anti-Kasha activatable chromophores, and its applicability in accurately quantifying multiple parameters with concentration-independence over space and time in biological and medical research. We anticipate that our strategy of tuning anti-Kasha/Kasha characteristics would inspire the creation of a generation of quantitative metabolic assays in a physiological context, thus greatly expanding the bio-analytical toolboxes for both basic life science research and clinical applications.

## Methods

### Materials and general methods

Unless special stated, all solvents and chemicals were purchased from commercial suppliers in analytical grade and used without further purification. The ^1^H and ^13^C NMR spectra were recorded on a Bruker AM 400 spectrometer, using TMS as an internal standard. High resolution mass spectrometry data were obtained with a Waters LCT Premier XE spectrometer. UV-vis absorption spectra were collected on a Varian Cary 500 spectrophotometer, and fluorescence spectrum measurements were performed on a Varian Cary Eclipse fluorescence spectrophotometer. Femtosecond time-resolved transient absorption spectra were recorded using HELIOS Fire Femtosecond Transient Absorption Spectrometer. Particle size was measured by dynamic light scattering (DLS) with a NICOMP 380 ZLS. Transmission electron microscopy (TEM) images were obtained on a JEOL 100CX transmission electron microscope operating at an accelerating bias voltage of 100 kV. HPLC analysis was performed on an Agilent 1100 series. Confocal fluorescence images were taken on a Leica TCS SP8 (63 × oil lens). In vivo fluorescence images were measured with a PerkinElmer IVIS Lumina Kinetic Series III imaging system. Synthesis methods for all compounds, characterization data are provided in the Supplementary Information.

### Theoretical calculation details

Density functional theory (DFT) and time-dependent (TD)-DFT calculations were performed using Gaussian 16^46^. Unless stated otherwise, geometry optimizations of all molecules in the ground (S_0_) and excited state (S_1_ and S_2_) were performed using the M062X functional and the def2SVP basis set in water^47^. Solvent effects were taken in account using the SMD model^48^. Frequency checks were carried out after each geometry optimization to ensure that the minima on the potential energy surfaces (PESs) were found. The radiation rates and non-radiation rates of compounds in aqueous solution were predicted using the MOlecular MAterials Property Prediction Package (MOMAP) 1.0 developed by Shuai group^49^. During the quantum yield prediction for open form, we had replaced the carboxyl phenyl ring with a hydrogen atom for two reasons: (1) MOMAP tends to overestimate the non-radiation rate associated with the slight rotation of the carboxyl phenyl ring upon photoexcitation, while this ring had little contribution to the UV–vis absorption and fluorescence properties of molecules (open form). (2) This replacement reduces computational load.

### Crystallography

CCDC 1909724 (BI-IFC-ester) contain the supplementary crystallographic data for this paper. These data can be obtained free of charge from the Cambridge Crystallographic Data Centre via www.ccdc.cam.ac.uk/data_request/cif.

### Cell lines

The cell lines were purchased from the Institute of Cell Biology (Shanghai, China). Cells were all propagated in T-75 flasks cultured at 37 °C under a humidified 5% CO_2_ atmosphere in RPMI-1640 medium or DMEM medium (GIBCO/Invitrogen, Camarillo, CA, USA), which were supplemented with 10% fetal bovine serum (FBS, Biological Industry, Kibbutz Beit Haemek, Israel) and 1% penicillin-streptomycin (10,000U mL^−1^ penicillin and 10 mg mL^−1^ streptomycin, Solarbio life science, Beijing, China).

### In vitro cellular imaging

The cells at 1 × 10^5^ cells/well were seeded onto glass-bottom petri dishes with complete medium (1.5 mL) for 12 h. Then the cells pre-incubated with and without GSH or NEM were exposed to desired concentrations of compounds for 0.5 h. PBS was used to wash cells for three times to clean the background. Four percent paraformaldehyde was added at room temperature for 20 min. The fixed cells were rinsed with PBS twice. The images were then photographed by using a confocal laser scanning microscope Leica TCS SP8 (63 × oil lens).

### Animals

All animal studies were approved by the Animal Care and Use Committee in accordance with the guidelines for the care and use of laboratory animals. The 3–4-week-old female BALB/cA nude mice were purchased from Shanghai Genechem Co.,Ltd., and maintained under standard conditions. The animals were housed in sterile cages within laminar airflow hoods in a specific pathogen-free room with a 12-h light/12-h dark schedule and fed autoclaved chow and water ad libitum. Production Permit No.: SCXK (Shanghai) 2013-0017. SYXK No. of Shanghai Institute of Materia Medica: SYXK (Shanghai) 2013-0049.

### Real-time in vivo imaging in tumor-bearing mice

The nude mice were inoculated with A549 cells on their right flanks by injecting 10^6^ cells subcutaneously. When the tumors grew up to 10 mm in diameter, DCM-IFC-4 in PBS were intravenously injected via tail vein into the A549 cell tumor-bearing nude mice. The real-time in vivo imaging was recorded at different time internals after DCM-IFC-4 injection. In vivo fluorescence images were measured with a PerkinElmer IVIS Lumina Kinetic Series III imaging system. After injection, the mice were sacrificed at 24 h. The grafted tumor tissues and major organs, including kidney, lung, spleen, liver, heart were excised and washed with 0.9% saline. The optical images of the organs and tissues were taken using a PE in vivo Professional Imaging System as described above.

### Reporting summary

Further information on research design is available in the Nature Research Reporting Summary linked to this article.

## Supplementary information

Supplementary Information

Reporting Summary

## Data Availability

The X-ray crystallographic coordinates for structures reported in this study have been deposited at the Cambridge Crystallographic Data Centre (CCDC), under deposition numbers 1909724. These data can be obtained free of charge from The Cambridge Crystallographic Data Centre via www.ccdc.cam.ac.uk/data_request/cif. The source data underlying Fig. 4d-h, j, k, 5d, e, h, i, l, m, and 6i-k, as well as Supplementary Figs. 15, 17, 24, 26, 31, 32, 34f and Supplementary Table 7 are provided as a Source Data file. All other data are available from the corresponding author.

## Source data

Source Data
