# Supplementary material for: De novo strategy with engineering anti-Kasha/Kasha fluorophores enables reliable ratiometric quantification of biomolecules
Source: Nat Commun. 2020 Feb 7;11:793. doi: 10.1038/s41467-020-14615-3 (PMC7005775; doi:10.1038/s41467-020-14615-3)
Supplement: Supplementary file 1 — Supplementary Information [file 41467_2020_14615_MOESM1_ESM.pdf]

Supplementary Information

De novo strategy with engineering anti-Kasha/Kasha fluorophores enables reliable ratiometric quantification of biomolecules

Limin Shi[1†], Chenxu Yan[1†], Zhiqian Guo[1*], Weijie Chi[2], Jingle Wei[3], Weimin Liu[3], Xiaogang Liu[2*], He Tian[1], and Wei-Hong Zhu[1*]

Supplementary Figures

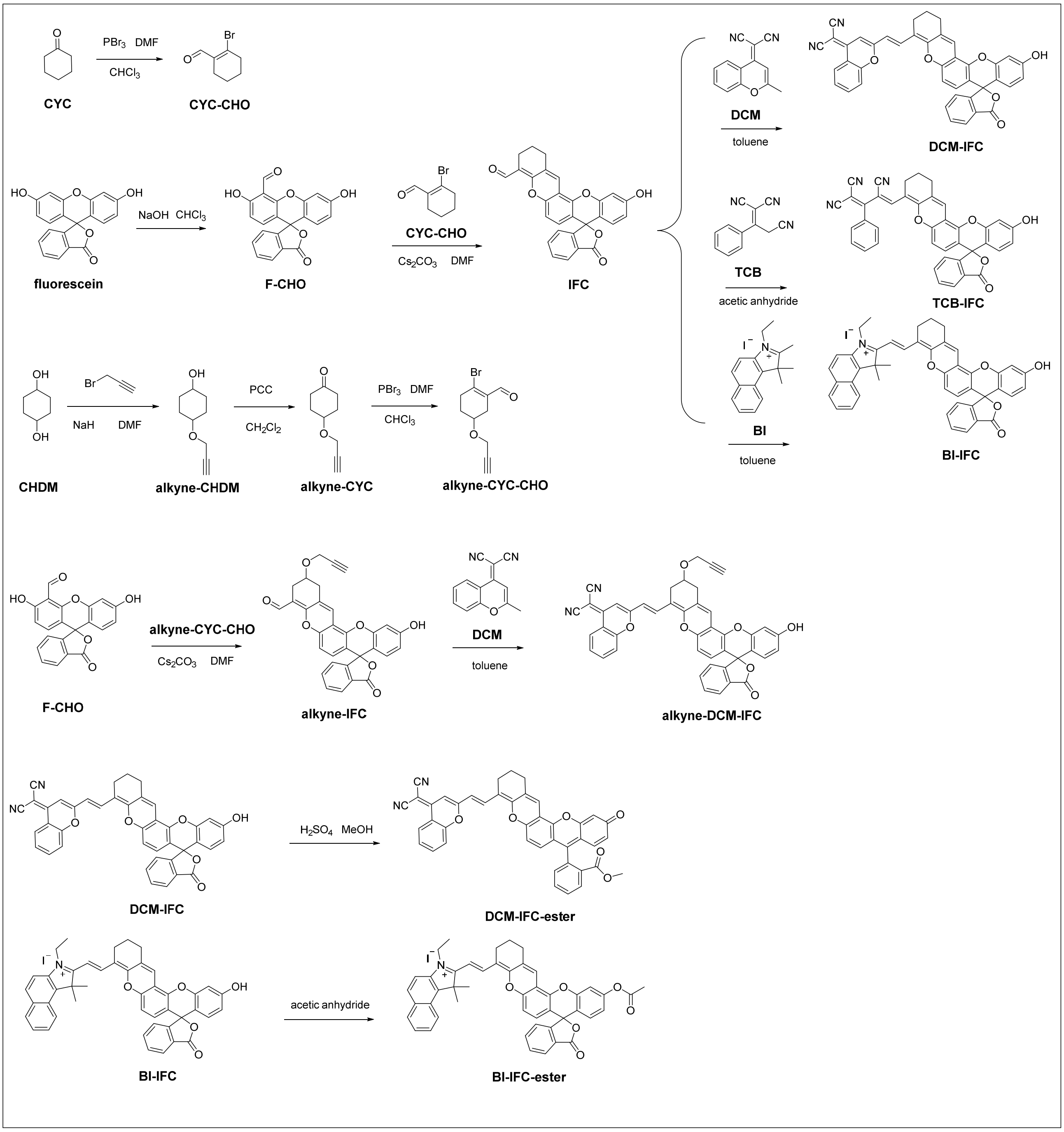

Supplementary Figure 1. Synthetic route of IFC family chromophores

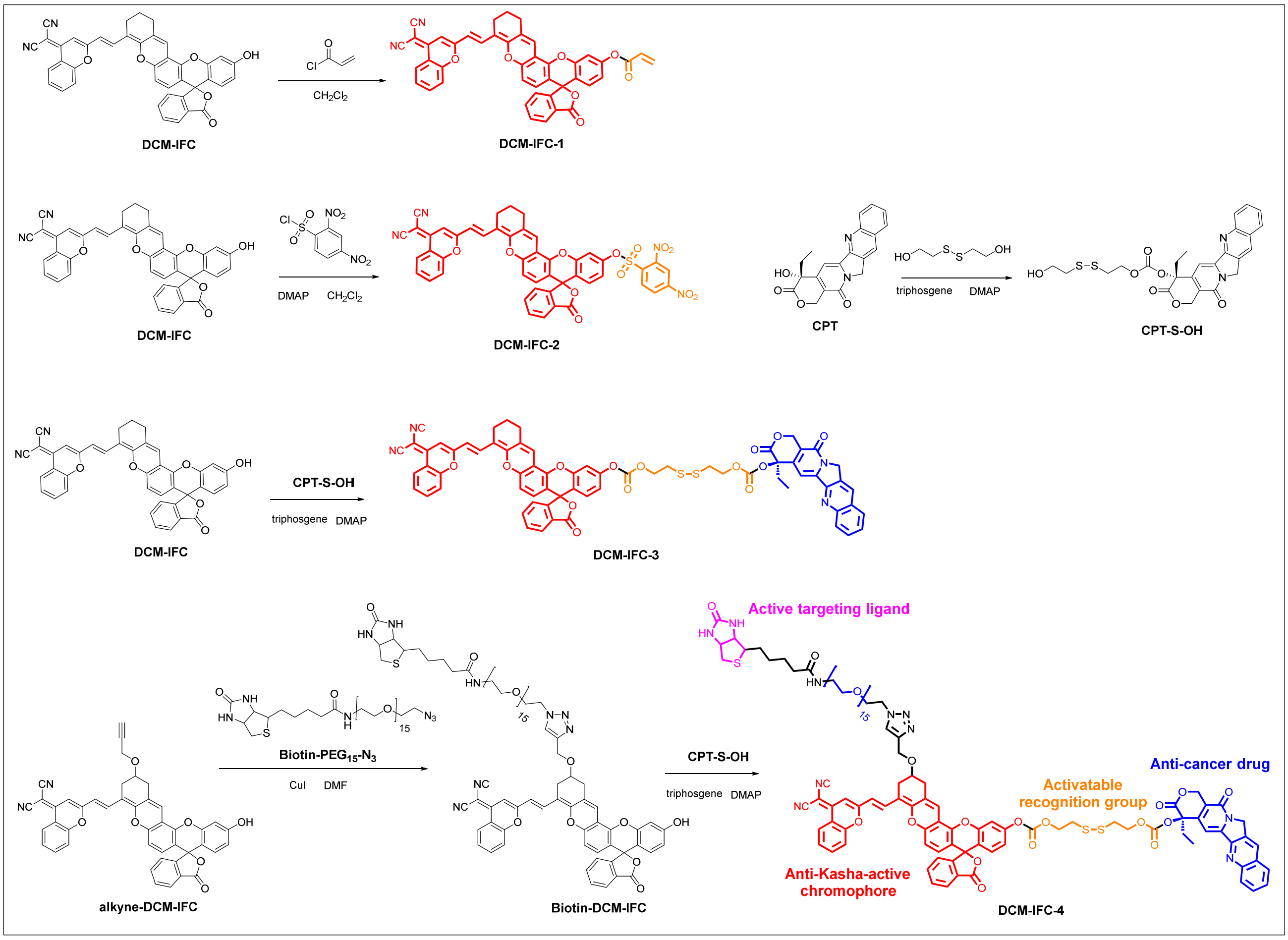

Supplementary Figure 2. Synthetic route of DCM-IFC probes

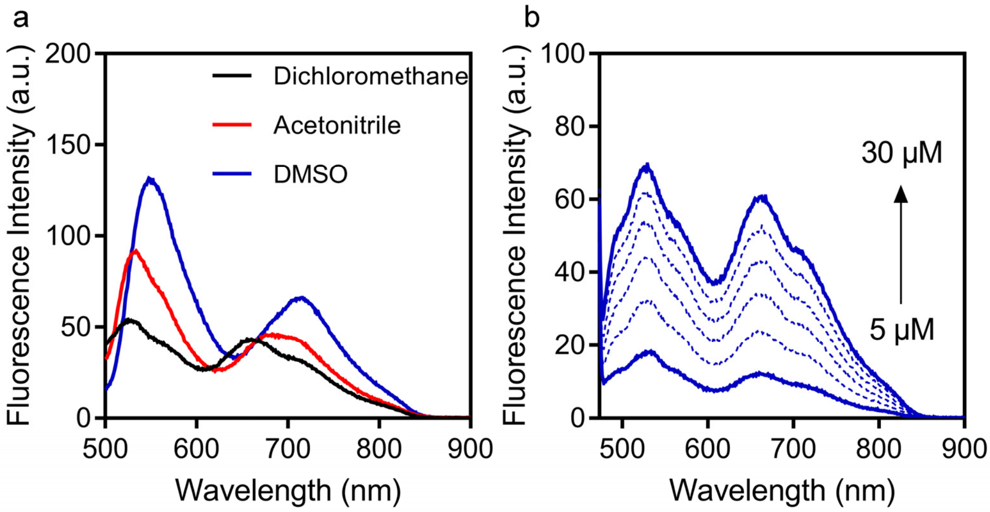

Supplementary Figure 3. Photoproperties of DCM-IFC-ester. Solvent-dependent (a) and concentration-dependent (b) of fluorescence spectra for DCM-IFC-ester, λ_{ex} = 480 nm.

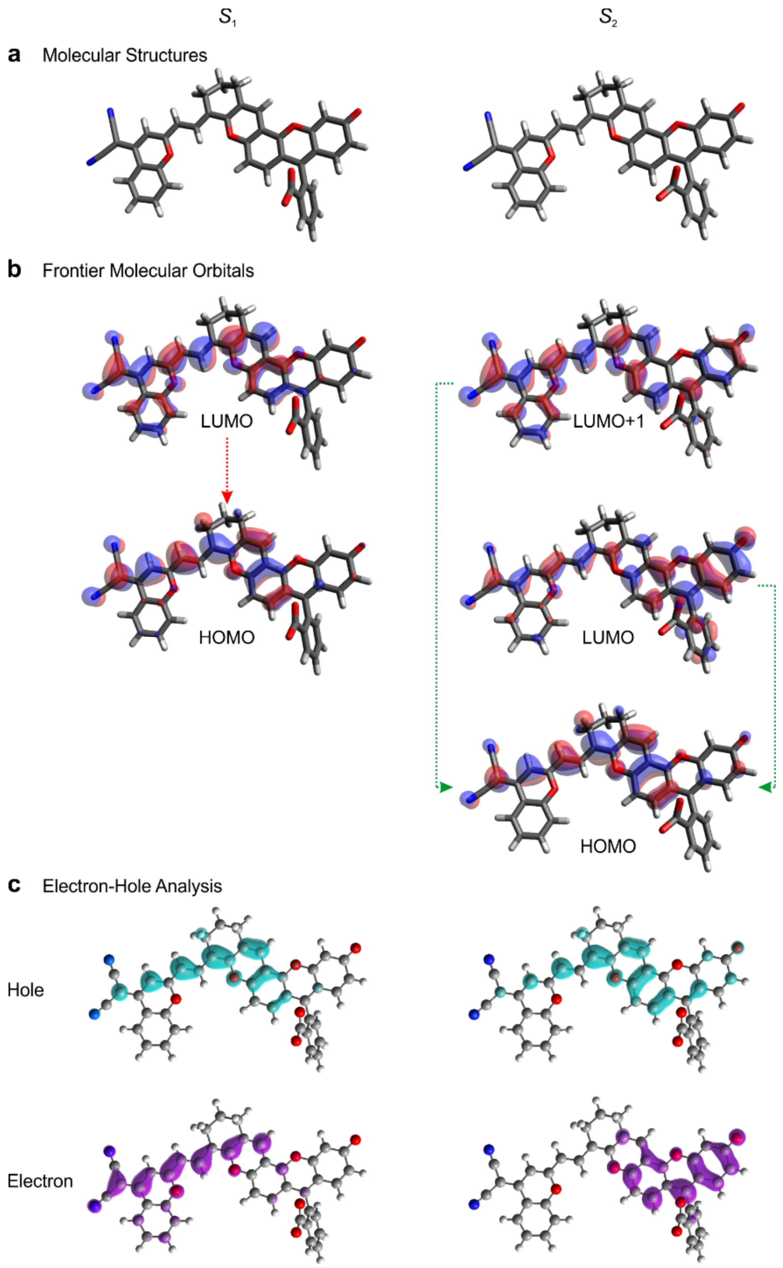

Supplementary Figure 4. Quantum chemical calculations of open-form DCM-IFC. Molecular structures (a), frontier molecular orbitals involved during the de-excitation (b) and electron-hole analysis (c) of the open form of DCM-IFC in the S_1 and S_2 states in water.

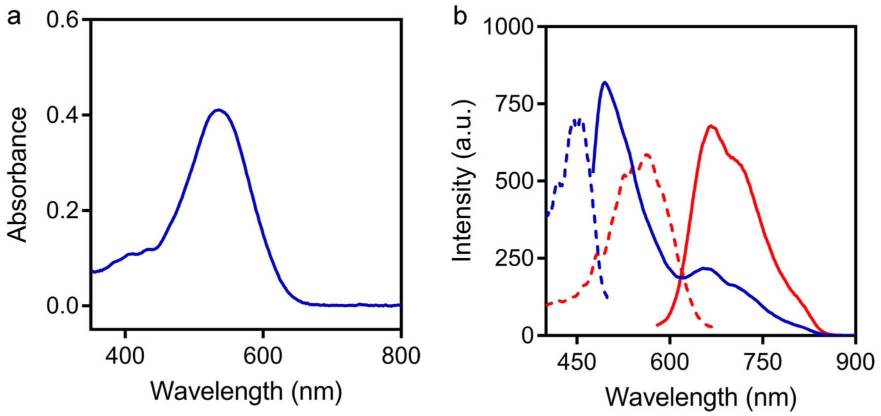

Supplementary Figure 5. Photoproperties of DCM-IFC-ester. (a) Absorption; (b) fluorescence spectra. Note: excitation spectra: dotted line, λ_{em} = 520 nm and 700 nm; emission spectra: solid line, λ_{ex} = 460 nm and 560 nm.

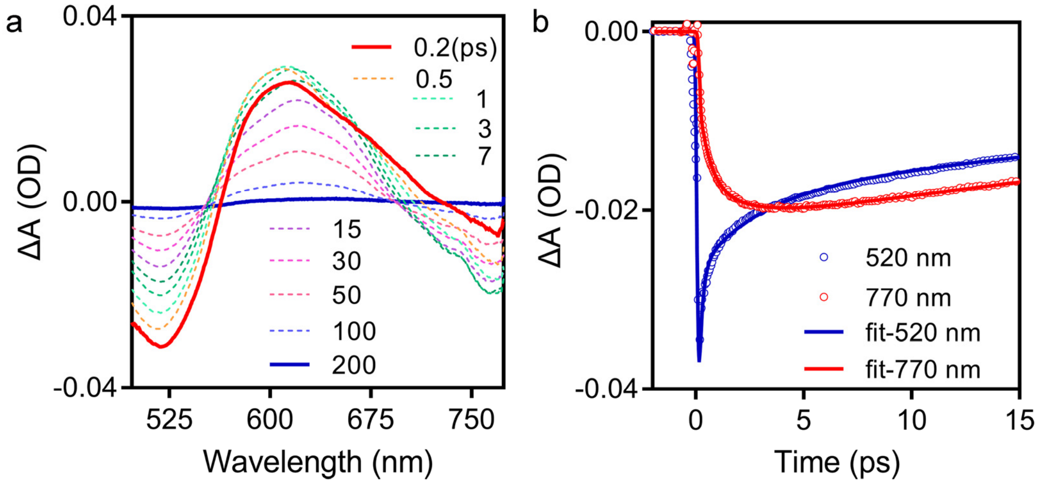

Supplementary Figure 6. Femtosecond time-resolved transient absorption spectra of DCM-IFC-ester. (a) Transient absorption spectra and (b) kinetics in dichloromethane.

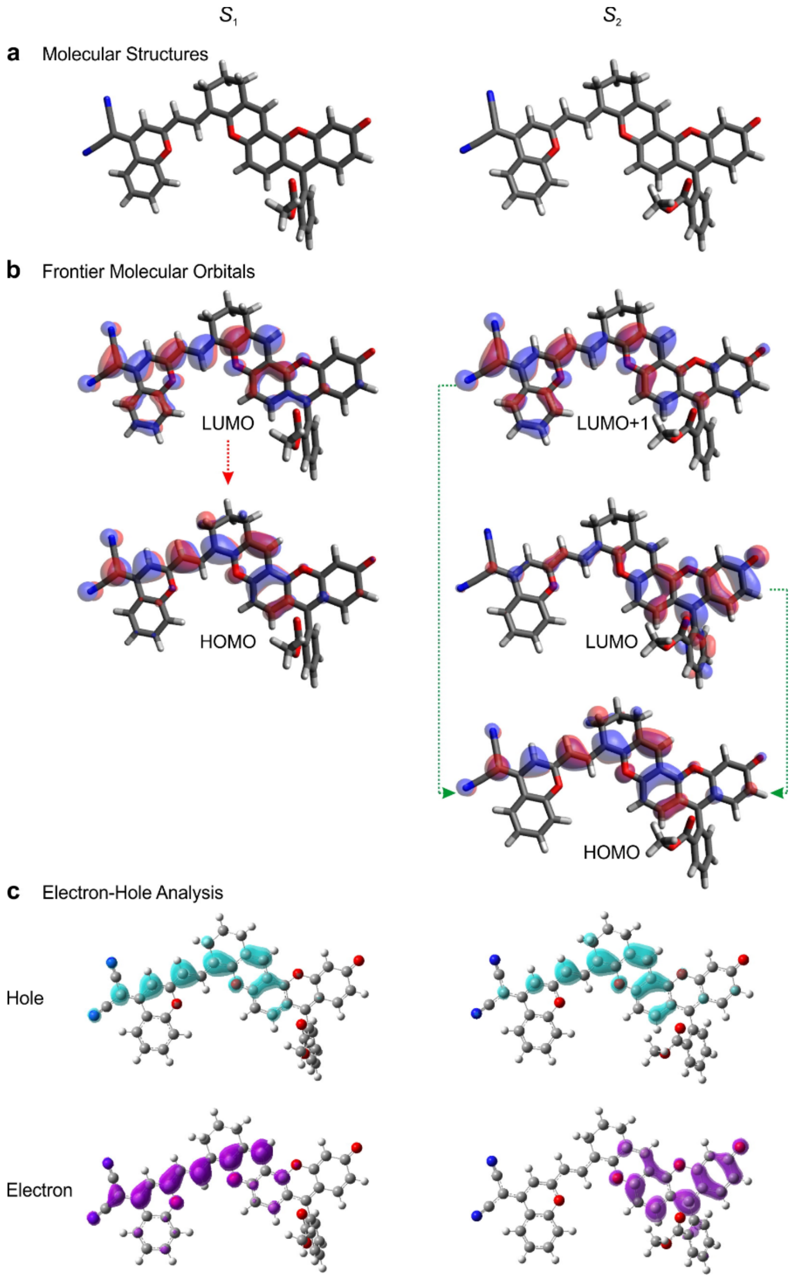

Supplementary Figure 7. Quantum chemical calculations of DCM-IFC-ester. Molecular structures (a), frontier molecular orbitals involved during the de-excitation (b) and electron-hole analysis (c) of the open form of DCM-IFC-ester in the S_1 and S_2 states in water. Note: LUMO and LUMO+1 swapped relative position during the optimization of the S_2 state, with respect to the corresponding orbitals as optimized in the S_1 state.

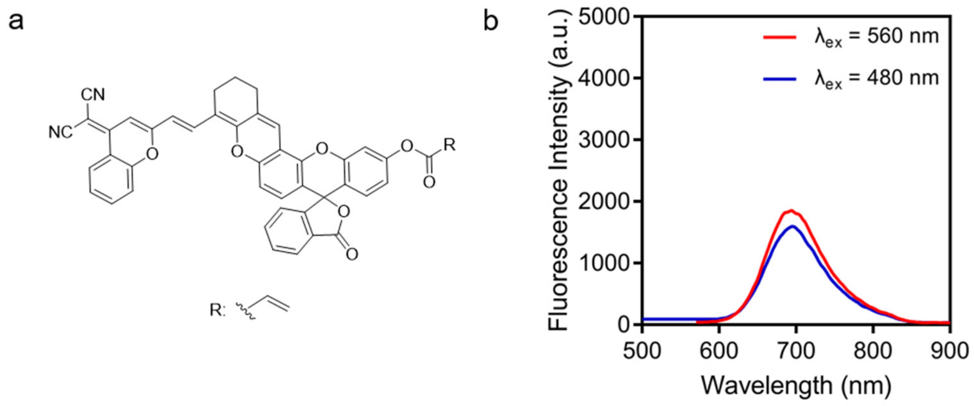

Supplementary Figure 8. Photoproperties of DCM-IFC-ester-R. (a) Structure and (b) fluorescence spectra of DCM-IFC-ester-R, λ_{ex} = 480 nm and 560 nm.

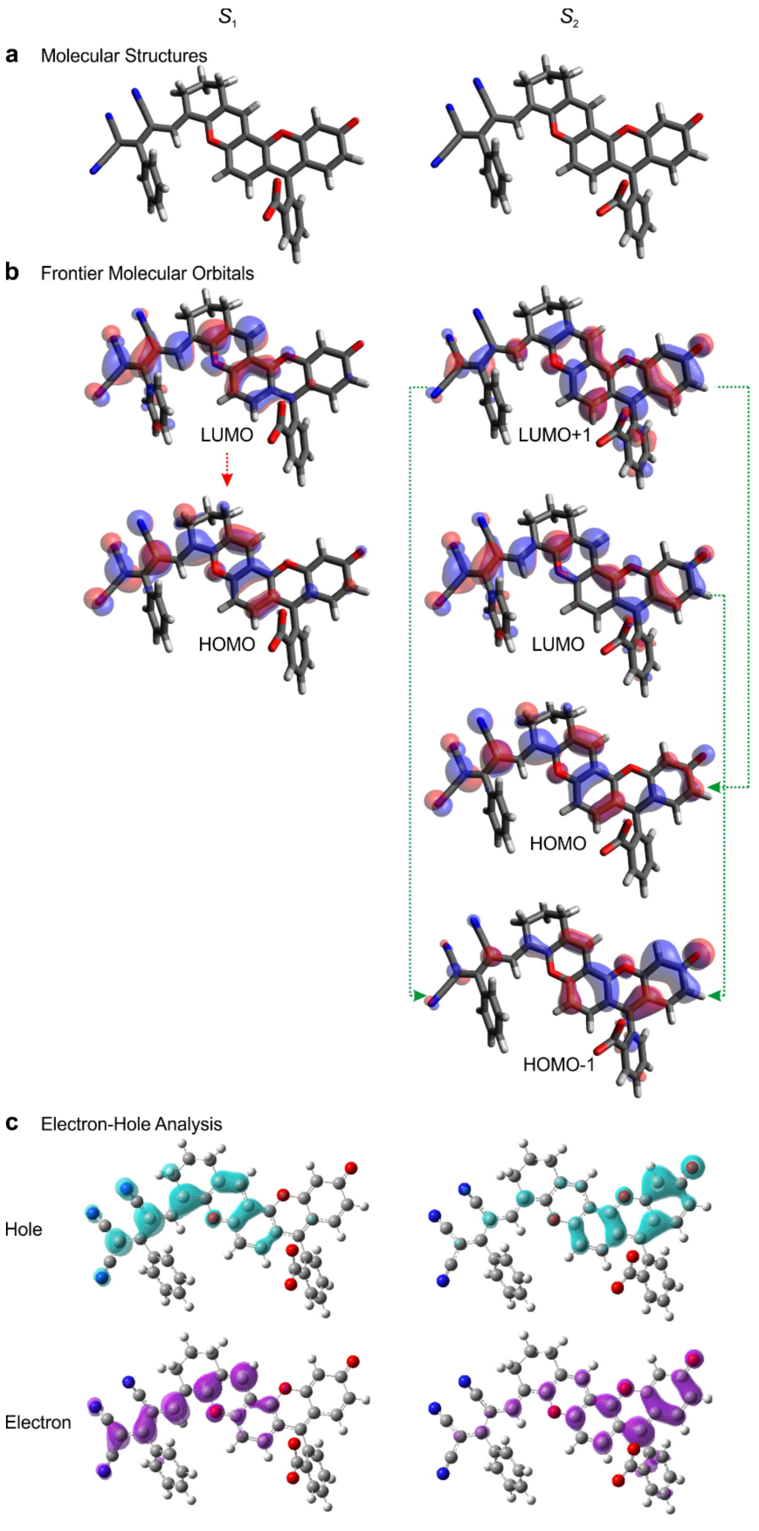

Supplementary Figure 9. Quantum chemical calculations of open-form TCB-IFC. Molecular structures (a), frontier molecular orbitals involved during the de-excitation (b) and electron-hole analysis (c) of the open form of TCB-IFC in the S_1 and S_2 states in water.

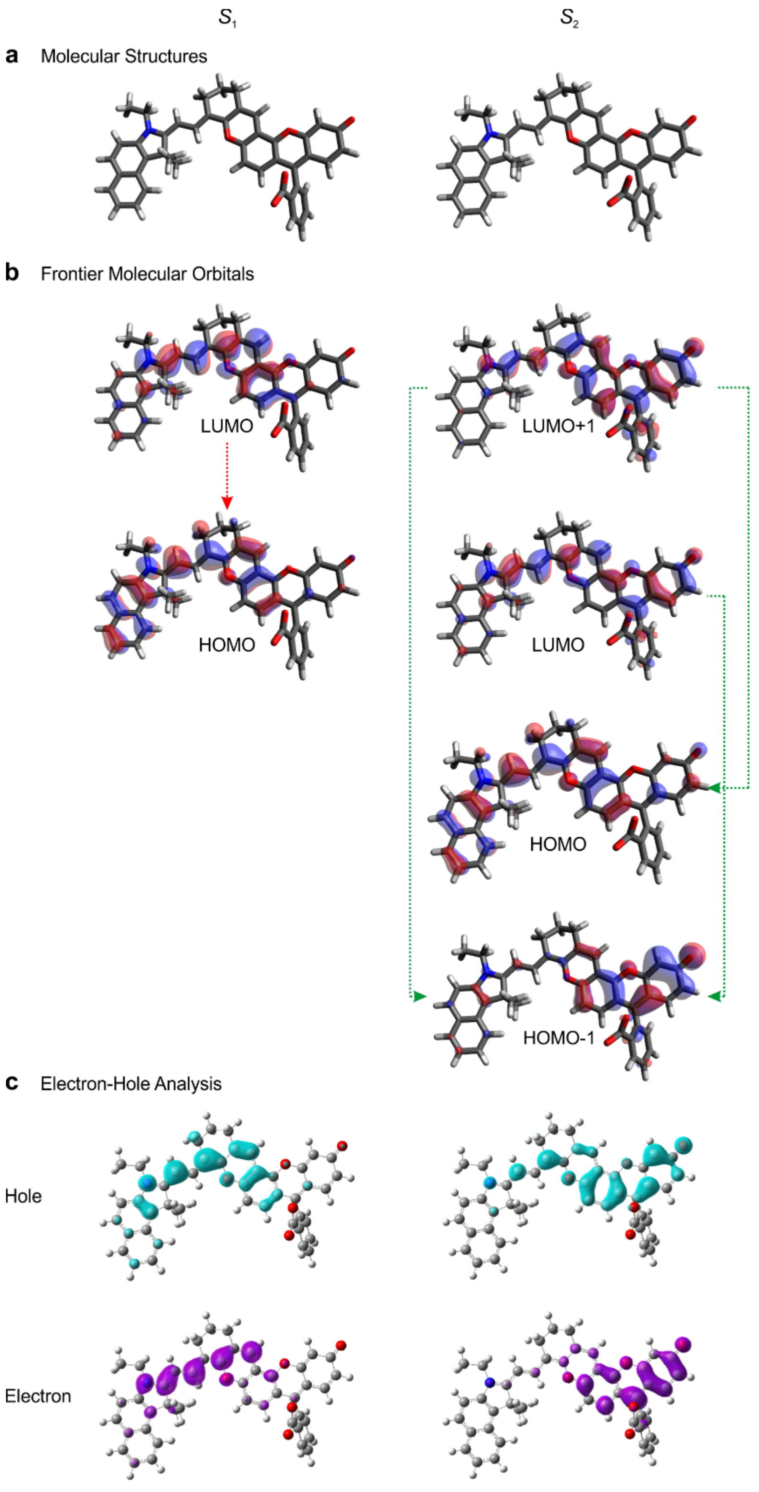

Supplementary Figure 10. Quantum chemical calculations of open-form BI-IFC. Molecular structures (a), frontier molecular orbitals involved during the de-excitation (b) and electron-hole analysis (c) of the open form of BI-IFC in the S_1 and S_2 states in water.

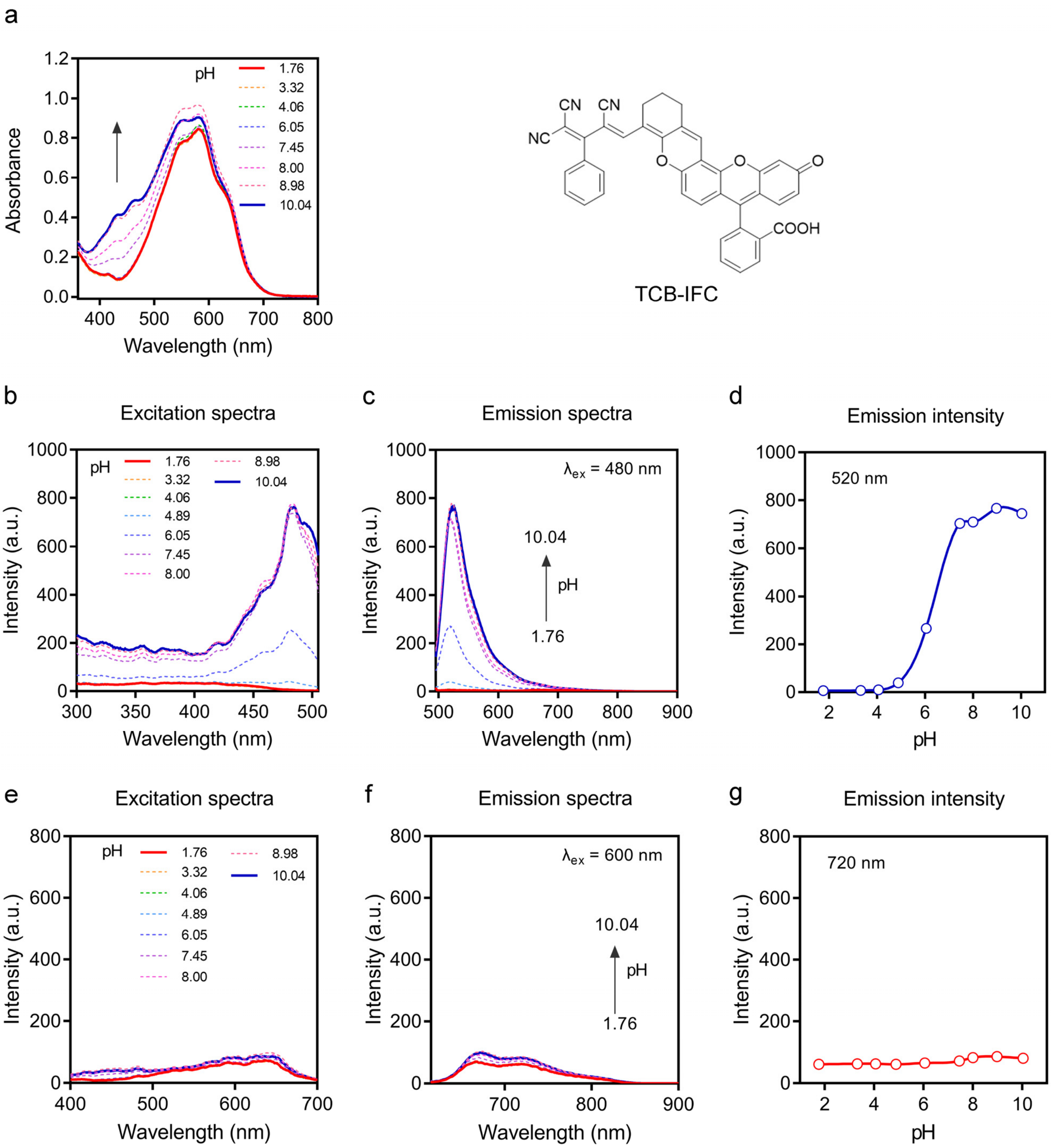

Supplementary Figure 11. Photophysical properties of TCB-IFC. (a) Absorbance spectra; (b) Chemical structure of TCB-IFC; (c) Excitation spectra monitored at 520 nm; (d) Emission spectra excited at 480 nm; (e) pH-dependent emission intensity monitored at 520 nm; (f) Excitation spectra monitored at 720 nm; (g) Emission spectra excited at 600 nm; (h) pH-dependent emission intensity monitored at 720 nm. Note: TCB-IFC. (20 μM) in a mixed solution of MeCN : Britton-Robinson buffer(7 : 3, 0.01 M) with pH changing from 1.76 to 10.04.

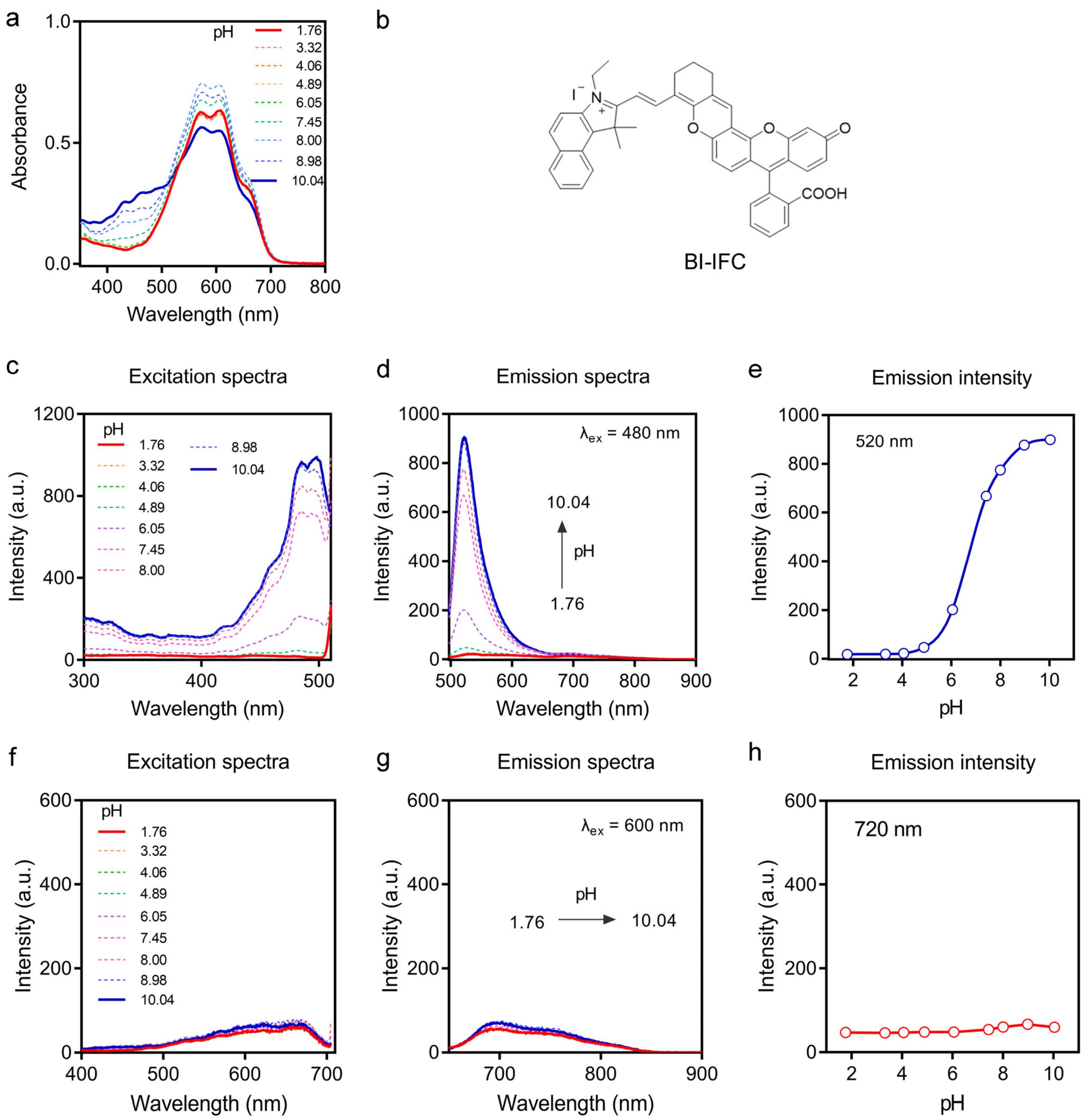

Supplementary Figure 12. Photophysical properties of BI-IFC. (a) Absorbance spectra; (b) Chemical structure of BI-IFC; (c) Excitation spectra monitored at 520 nm; (d) Emission spectra excited at 480 nm; (e) pH-dependent emission intensity monitored at 520 nm; (f) Excitation spectra monitored at 720 nm; (g) Emission spectra excited at 600 nm; (h) pH-dependent emission intensity monitored at 720 nm. Note: BI-IFC. (20 μM) in a mixed solution of MeCN : Britton-Robinson buffer(7 : 3, 0.01 M) with pH changing from 1.76 to 10.04.

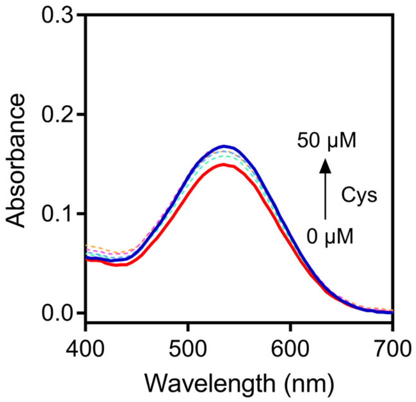

Supplementary Figure 13. Absorbance spectra of DCM-IFC-1 with the titration of Cys. Note: DCM-IFC-1 (10 μM) with the titration of Cys and incubation for 20 min in a mixture solution of MeCN/ phosphate-buffered saline (PBS) (7/3, v:v, pH = 7.4), λ_{ex} = 480 nm.

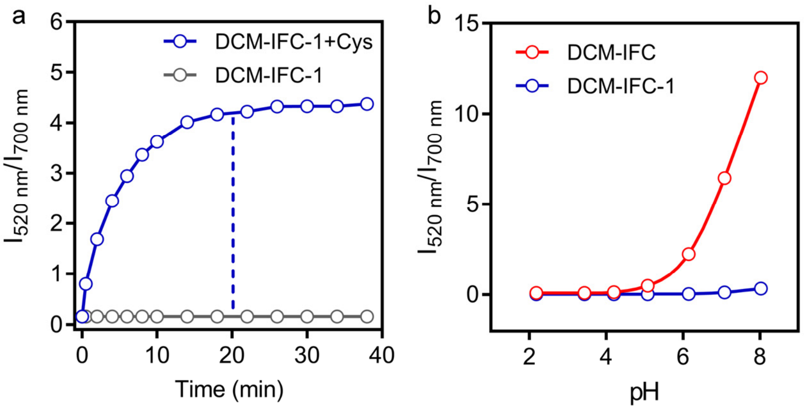

Supplementary Figure 14. Photoproperties of DCM-IFC-1. (a) Time-dependent of $I_{520\ nm}/I_{700\ nm}$ (0–40 min) for DCM-IFC-1 (10 μM) in a mixture solution of MeCN/PBS (7/3, v:v, pH = 7.4) with (blue) or without (gray) Cys, λ_{ex} = 480 nm. (b) pH-dependent of $I_{520\ nm}/I_{700\ nm}$ for DCM-IFC-1 and DCM-IFC (10 μM) in a mixture solution of MeCN/Britton-Robinson buffer (7/3, v:v, 0.01 M) for various pH, λ_{ex} = 480 nm.

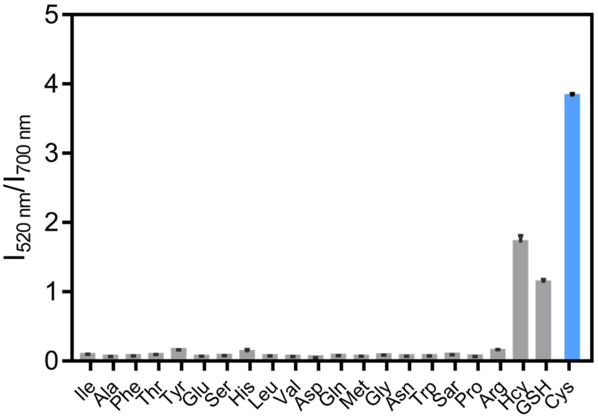

Supplementary Figure 15. Selectivity of DCM-IFC-1. Fluorescence ratio ($I_{520\ nm}/I_{700\ nm}$) responses of DCM-IFC-1 (10 μM) toward various amino acids in a mixture solution of MeCN/PBS (7/3, v:v, pH = 7.4). Data with error bars are expressed as mean ± s.d., n = 3. Source data are provided as a Source Data file.

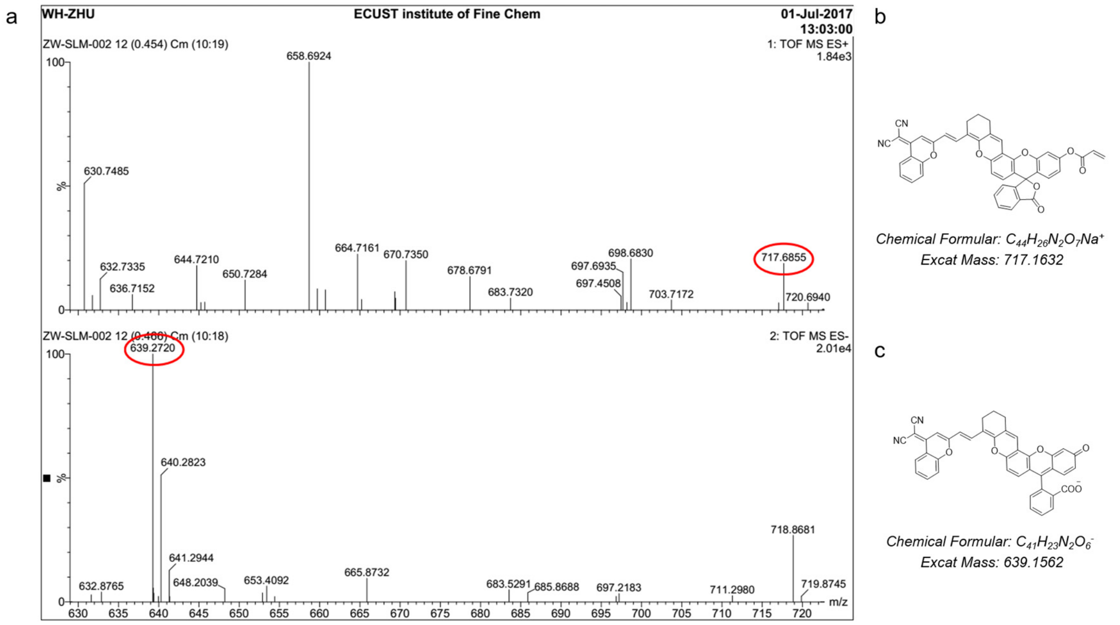

Supplementary Figure 16. ESI-MS spectra of the products from the reaction of DCM-IFC-1 with Cys. (a) ESI-MS spectra; (b) Chemical structure of DCM-IFC-1; (c) Chemical structure of product.

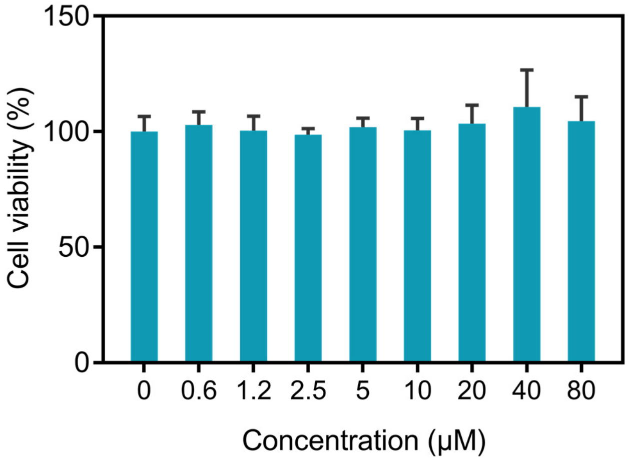

Supplementary Figure 17. Cytotoxicity of DCM-IFC-1. Relative viability of HeLa cells *in vitro* after incubation for 24 h with DCM-IFC-1 at various concentrations. Data with error bars are expressed as mean ± s.d., n = 5. Source data are provided as a Source Data file. Note: DCM-IFC-1 has minimal toxicity and enjoy superior biocompatibility toward cultured cell lines.

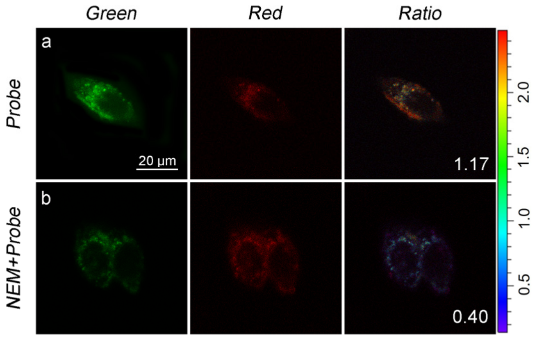

Supplementary Figure 18. Ratiometric imaging of DCM-IFC-1 in living cells. HeLa cells incubated with DCM-IFC-1 (10 μM): without (a) and with (b) NEM. Note: the green channel was 520± 20 nm, the red channel was 700± 20 nm, and ratiometric images were generated from the 520 nm and 700 nm, λ_{ex} = 488 nm.

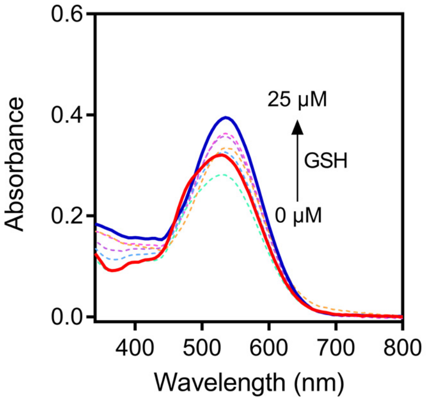

Supplementary Figure 19. Absorbance spectra of DCM-IFC-2 incubation with GSH. Note: DCM-IFC-2 (10 μM) with the titration of GSH and incubation for 35 min in a mixture solution of MeCN/PBS (7/3, v:v, pH = 7.4), λ_{ex} = 480 nm.

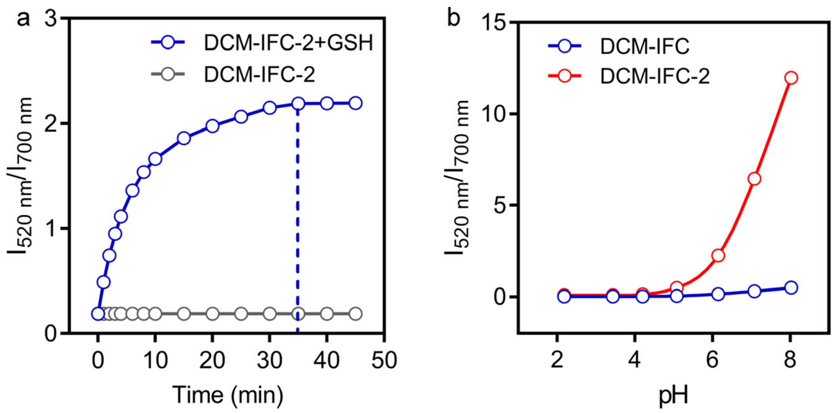

Supplementary Figure 20. Photoproperties of DCM-IFC-2. (a) Time-dependent of $I_{520\ nm}/I_{700\ nm}$ (0−45 min) for DCM-IFC-2 (10 μM) in a mixture solution of MeCN/PBS (7/3, v:v, pH = 7.4) with (blue) or without (gray) GSH, λ_{ex} = 480 nm. (b) pH-dependent of $I_{520\ nm}/I_{700\ nm}$ for DCM-IFC-2 and DCM-IFC (10 μM) in a mixture solution of MeCN/Britton-Robinson buffer (7/3, v:v, 0.01 M) for various pH, λ_{ex} = 480 nm.

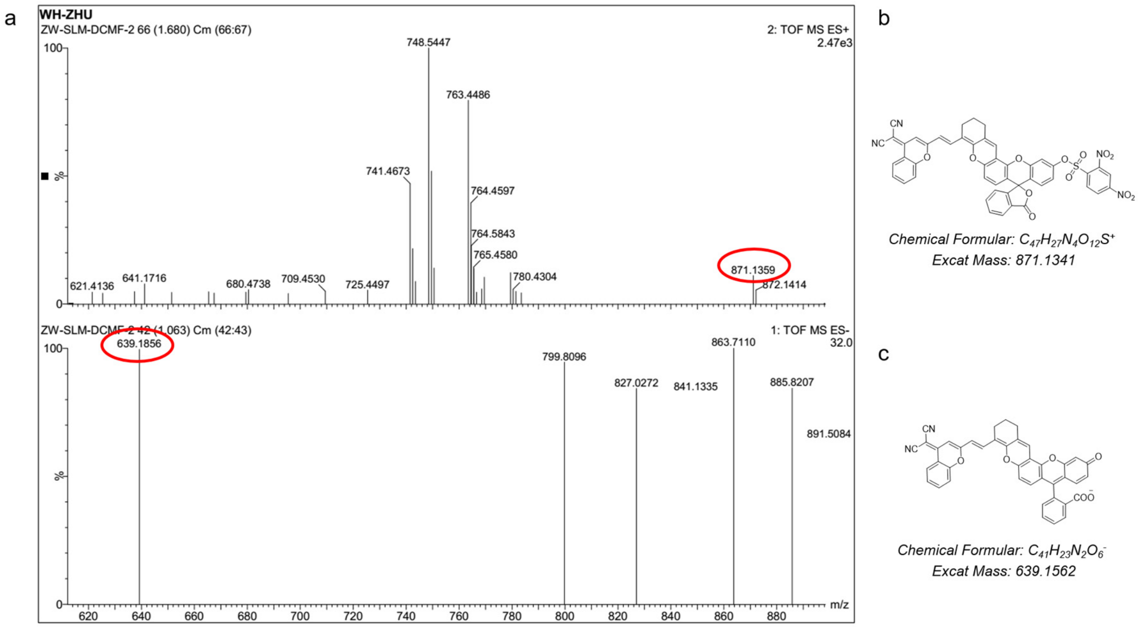

Supplementary Figure 21. ESI-MS spectra of the products from the reaction of DCM-IFC-2 with GSH. (a) ESI-MS spectra; (b) Chemical structure of DCM-IFC-2; (c) Chemical structure of product.

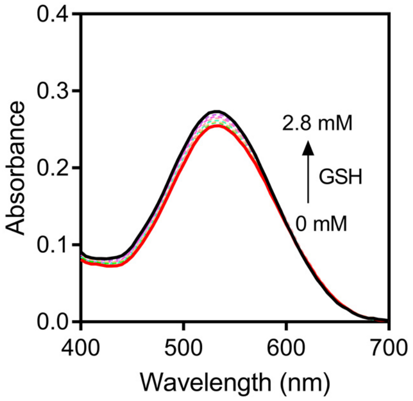

Supplementary Figure 22. Absorbance spectra of DCM-IFC-3/4 incubation with GSH. Note: DCM-IFC-3/4 (10 μM) with the titration of GSH and incubation for 40 min in a mixture solution of MeCN/PBS (pH = 7.4), λ_{ex} = 480 nm.

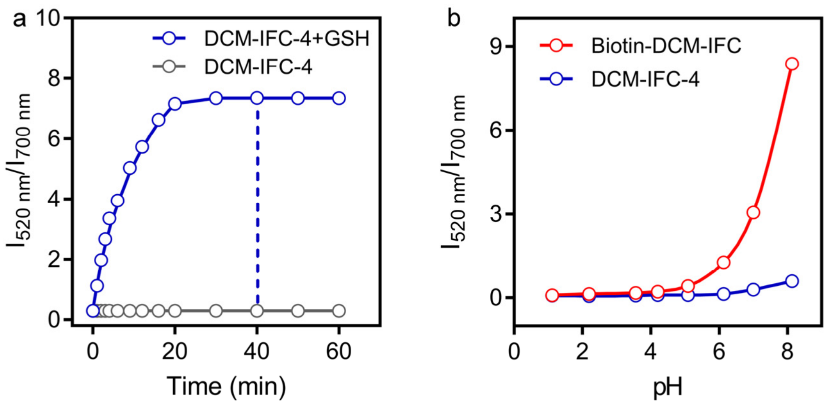

Supplementary Figure 23. Photoproperties of DCM-IFC-4. (a) Time-dependent of $I_{520\ nm}/I_{700\ nm}$ (0−60 min) for DCM-IFC-4 (10 μM) in a mixture solution of MeCN/PBS (1/1, v:v, pH = 7.4) with (blue) or without (gray) GSH, λ_{ex} = 480 nm. (b) pH-dependent of $I_{520\ nm}/I_{700\ nm}$ for DCM-IFC-4 and Biotin-DCM-IFC (10 μM) in a mixture solution of MeCN/Britton-Robinson buffer (1/1, v:v, 0.01 M) for various pH, λ_{ex} = 480 nm.

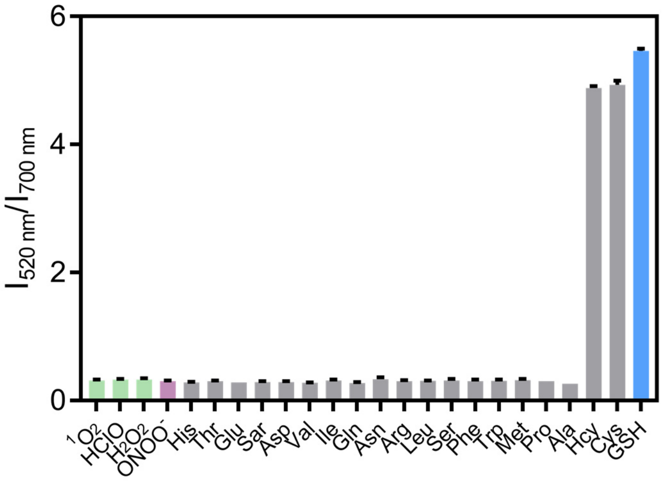

Supplementary Figure 24. Selectivity of DCM-IFC-4. Fluorescence ratio ($I_{520\ nm}/I_{700\ nm}$) responses of DCM-IFC-4 (10 μM) toward various ROS, RNS, and amino acids in a mixture solution of MeCN/PBS (1/1, v:v, pH = 7.4). Data with error bars are expressed as mean ± s.d., n = 3. Source data are provided as a Source Data file.

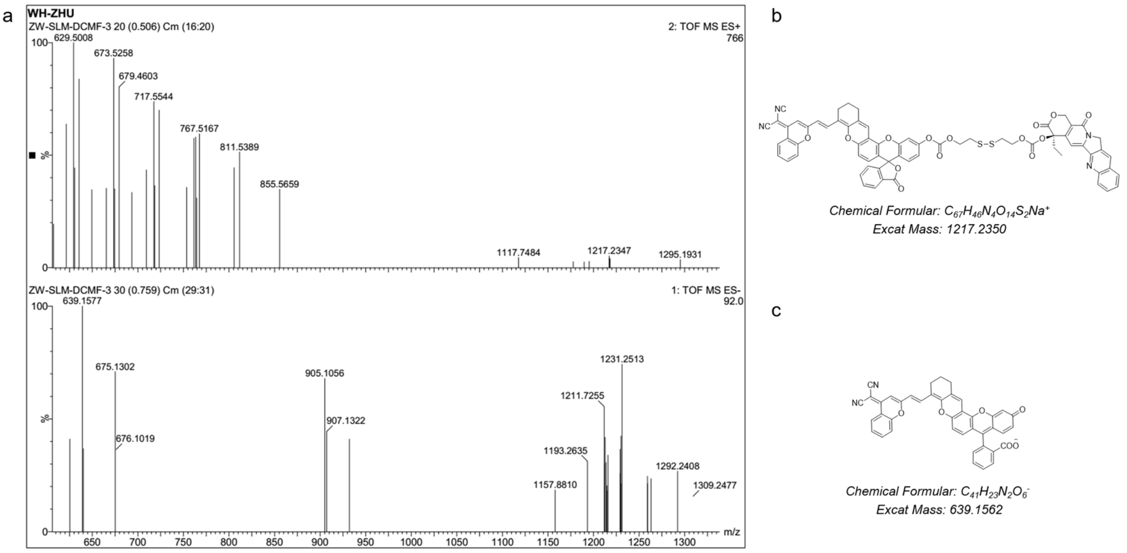

Supplementary Figure 25. ESI-MS spectrum of the products from the reaction of DCM-IFC-3 with GSH. (a) ESI-MS spectra; (b) Chemical structure of DCM-IFC-3; (c) Chemical structure of product.

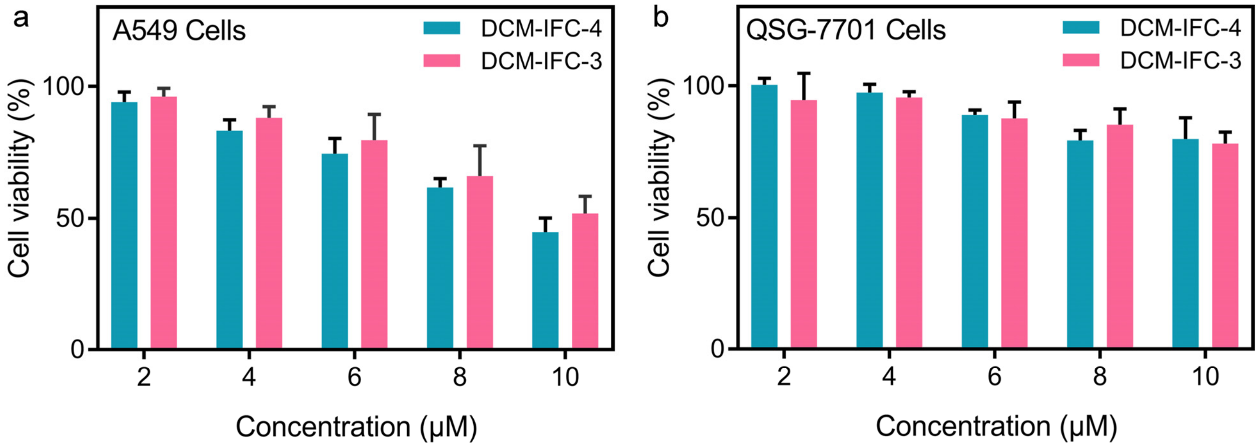

Supplementary Figure 26. Cytotoxicity of DCM-IFC-4 and DCM-IFC-3. (a) A549 cells (cancer cells) and (b) QSG-7701 cells (normal cells) were incubated with various concentrations (0-10 μM) of DCM-IFC-4 and DCM-IFC-3 for 24 h. Data with error bars are expressed as mean ± s.d., n = 3. Source data are provided as a Source Data file.

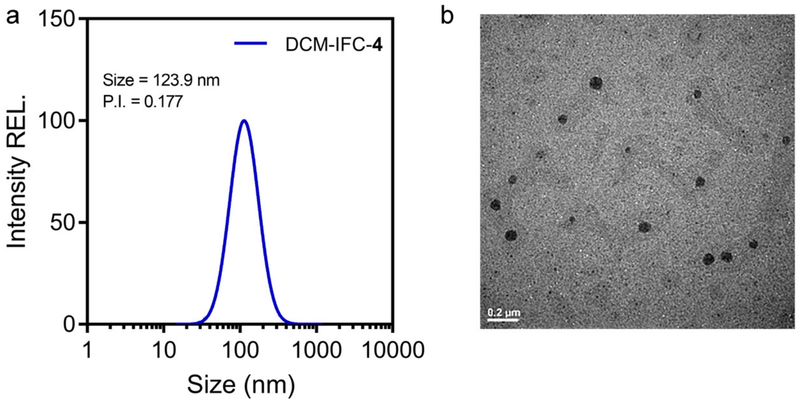

Supplementary Figure 27. Size distribution and TEM image of DCM-IFC-4. (a) Size distribution and (b) TEM imag of DCM-IFC-4 at 10 μM in water.

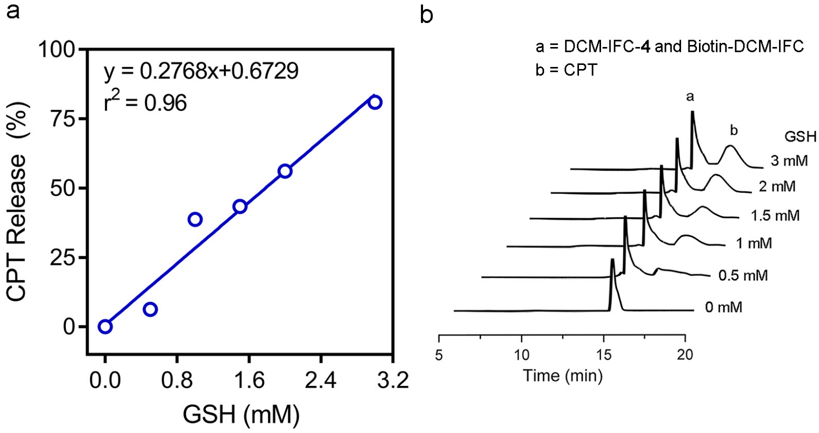

Supplementary Figure 28. CPT release of DCM-IFC-4 with increasing concentrations of GSH. (a) *In vitro* CPT release in a mixture solution of MeCN/PBS (1/1, v:v, pH = 7.4) with increasing concentrations of GSH. (b) The increase of CPT concentrations after treatment with increasing concentrations of GSH was determined by HPLC.

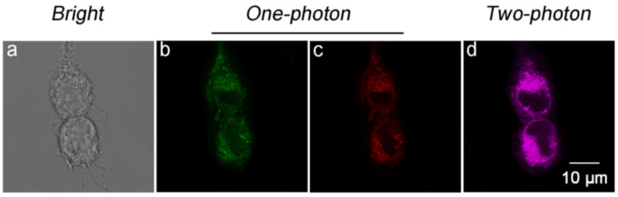

Supplementary Figure 29. Two-photon properties of DCM-IFC. CLSM images of DCM-IFC (10 μM). Bright field (a), and one-photon image (λ_{ex} = 488 nm) of HeLa cells (b, λ_{ex} = 520-600 nm; c, λ_{em} = 620-750 nm). (c) Two-photon image (λ_{ex} = 800 nm) of HeLa cells (λ_{em} = 500-750 nm).

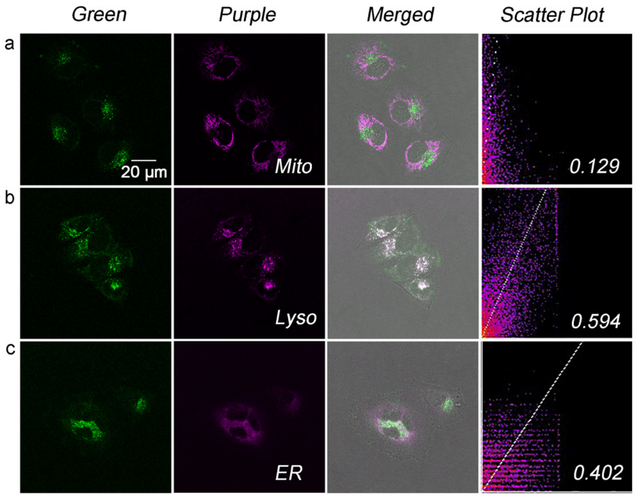

Supplementary Figure 30. Co-localization experiments of DCM-IFC. CLSM images for intracellular localization of DCM-IFC in HeLa cells. Cells were incubated with DCM-IFC (10 μM, green channel, λ_{ex} = 480 nm; λ_{em} = 520-600 nm) for 2 h and then co-stained with mitochondria-targeting tracker (a, 50 nM Mito Tracker Deep Red FM for 30 min, λ_{ex} = 630 nm; λ_{em} = 650-700 nm), lysosome-targeting tracker (b, 50 nM Lyso Tracker Red DND-99 for 60 min, λ_{em} = 560 nm; λ_{em} = 570-630 nm), and endoplasmic reticulum-targeting tracker (c, 1 μM ER Tracker Red for 30 min, λ_{ex} = 560 nm; λ_{em} = 570-630 nm).

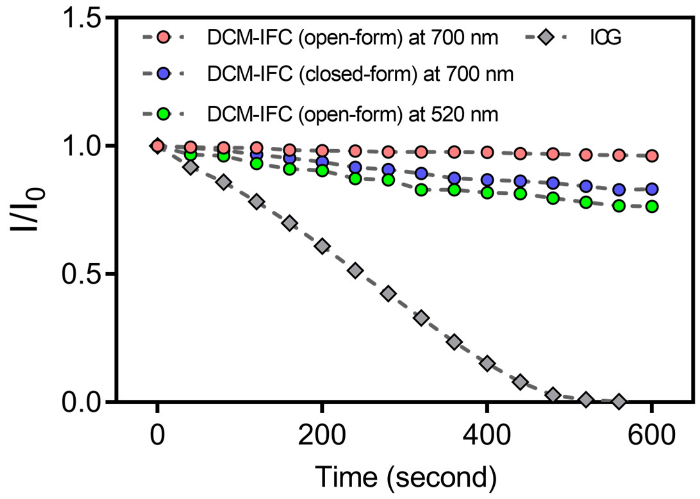

Supplementary Figure 31. Photostability of DCM-IFC. Time-dependent fluorescence intensity of DCM-IFC (5 μM) and ICG (5 μM) under sustained illumination (Hamamatsu, LC8 Lightningcure, 300 W). Source data are provided as a Source Data file.

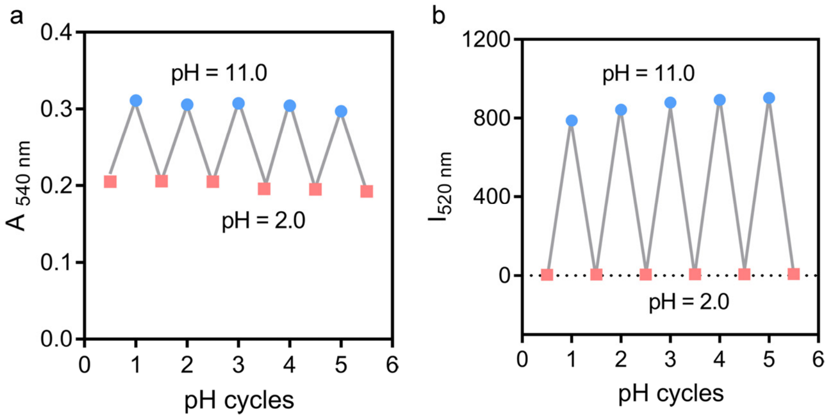

Supplementary Figure 32. Reversible pH-dependent properties of DCM-IFC. The reversible pH-dependent absorption (a) and fluorescence (b) changes of DCM-IFC. Conditions: 10 μM of DCM-IFC in a mixture solution of MeCN/ water (7/3, v:v), λ_{ex} = 480 nm. The pH was adjusted by HCl (aq) and NaOH (aq). Source data are provided as a Source Data file.

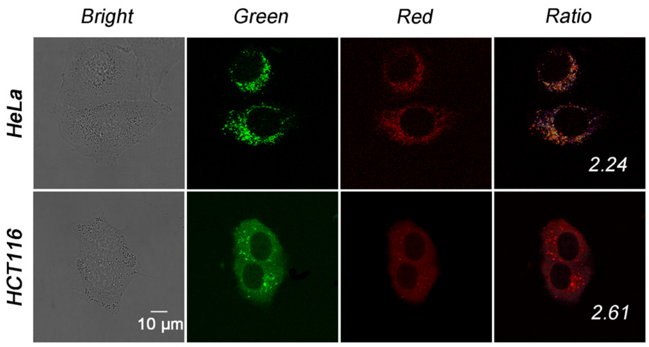

Supplementary Figure 33. Quantitative sensing GSH in different cancer cells with DCM-IFC-4. Dual-channel and ratiometric imaging of HeLa and HCT116 cells incubated with DCM-IFC-4 (10 μM). Note: The green channel was 520 ± 20 nm, the red channel was 700 ± 20 nm, and ratiometric images were generated from the 520 and 700 nm channels, λ_{ex} = 488 nm.

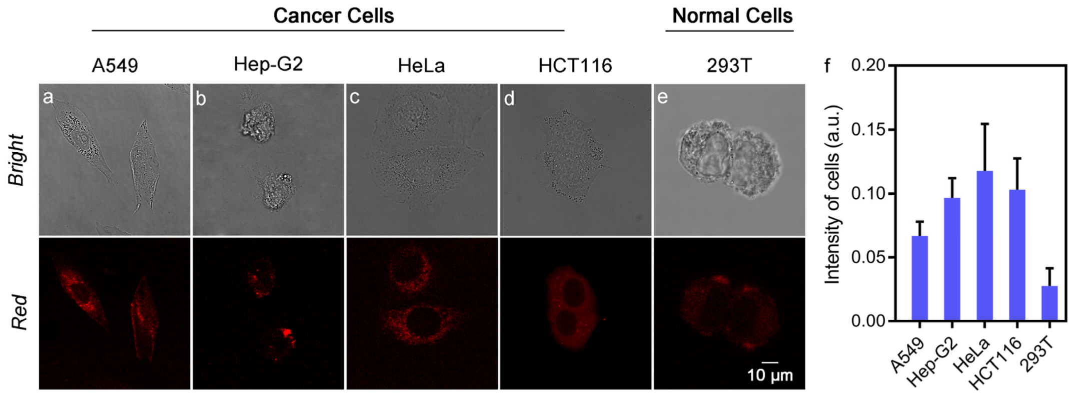

Supplementary Figure 34. Targeting properties of biotin-functionalized DCM-IFC-4. (a-e) CLSM images of A549, Hep-G2, HeLa, HCT116 and 293T cells incubated with DCM-IFC-4 (10 μM, red channel, λ_{ex} = 480 nm; λ_{em} = 700 ± 20 nm). (f) Fluorescence intensity of different cell lines. Data with error bars are expressed as mean ± s.d., n = 3. Source data are provided as a Source Data file.

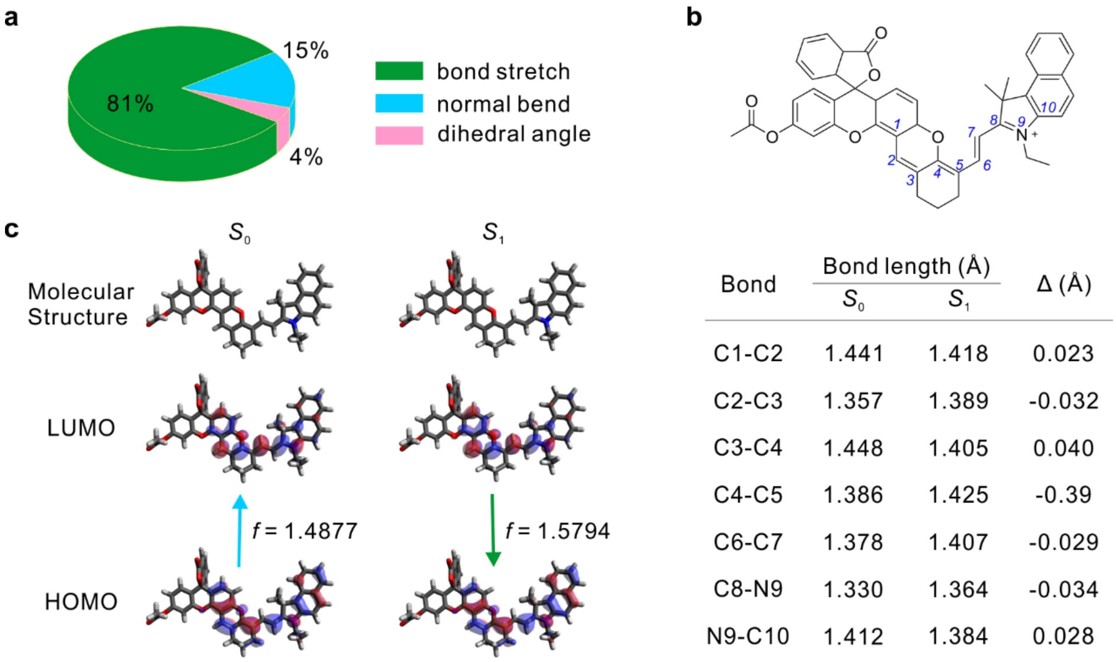

Bond	Bond length (Å) S_0	Bond length (Å) S_1	Δ (Å)
C1-C2	1.441	1.418	0.023
C2-C3	1.357	1.389	-0.032
C3-C4	1.448	1.405	0.040
C4-C5	1.386	1.425	-0.39
C6-C7	1.378	1.407	-0.029
C8-N9	1.330	1.364	-0.034
N9-C10	1.412	1.384	0.028

Supplementary Figure 35. Theoretical calculation of BI-IFC-ester. (a) Contributions to the reorganization energy of BI-IFC-ester upon photoexcitation from the S_0 to S_1 states; (b) Chemical structure of BI-IFC-ester and key bond lengths involved in the bond stretches after photoexcitation; (c) Optimized geometries, LUMO, HOMO and oscillator strength (f) of BI-IFC-ester in the S_0 and S_1 states. All calculations are performed in water. All calculations are performed in water. Note: (a, b) The significant non-radiative rate mainly derived from the molecular structural changes of several bond stretches upon photo-excitations, which contribute to 81% of the reorganization energy. (c) This fragment with substantial bond stretches are heavily involved in the light absorption and emission of BI-IFC-ester, as the HOMO and LUMO of BI-IFC-ester resides in it.

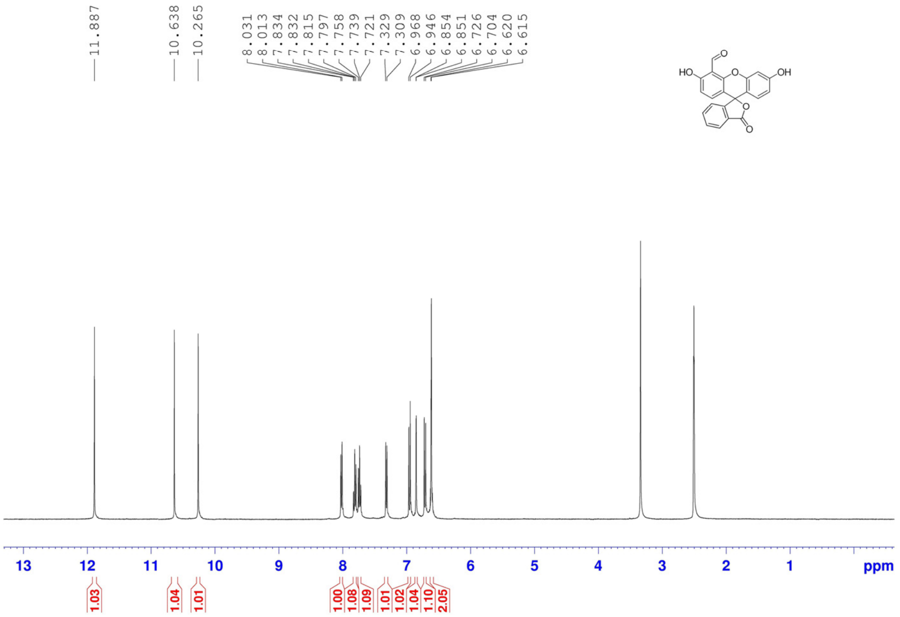

Supplementary Figure 36. ^{1}H NMR spectrum of F-CHO in DMSO-d_6

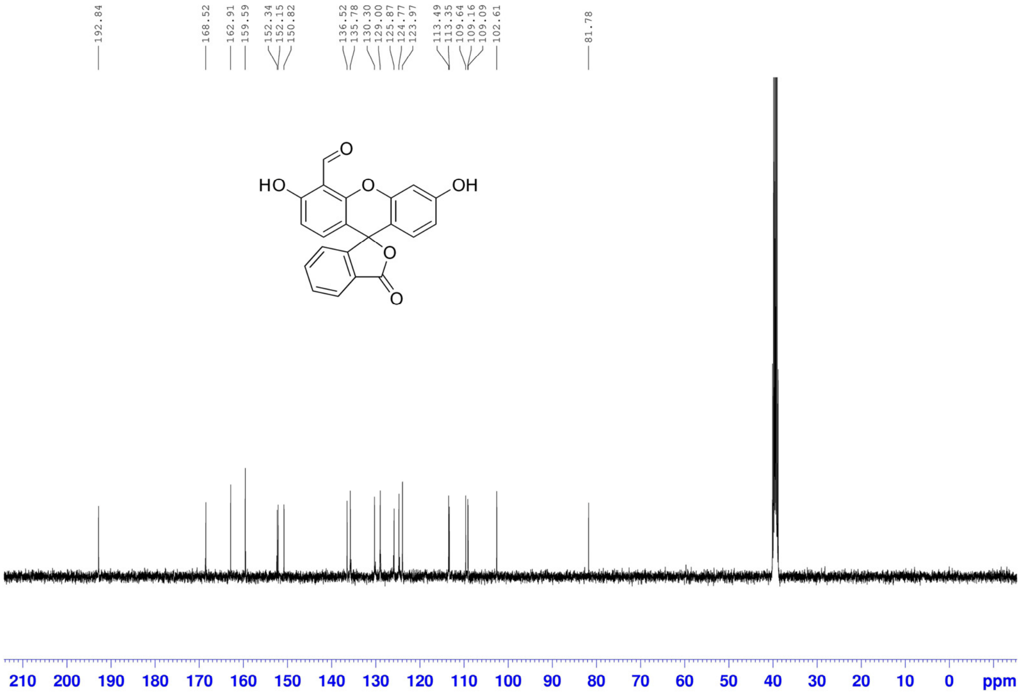

Supplementary Figure 37. ^{13}C NMR spectrum of F-CHO in DMSO-d_6

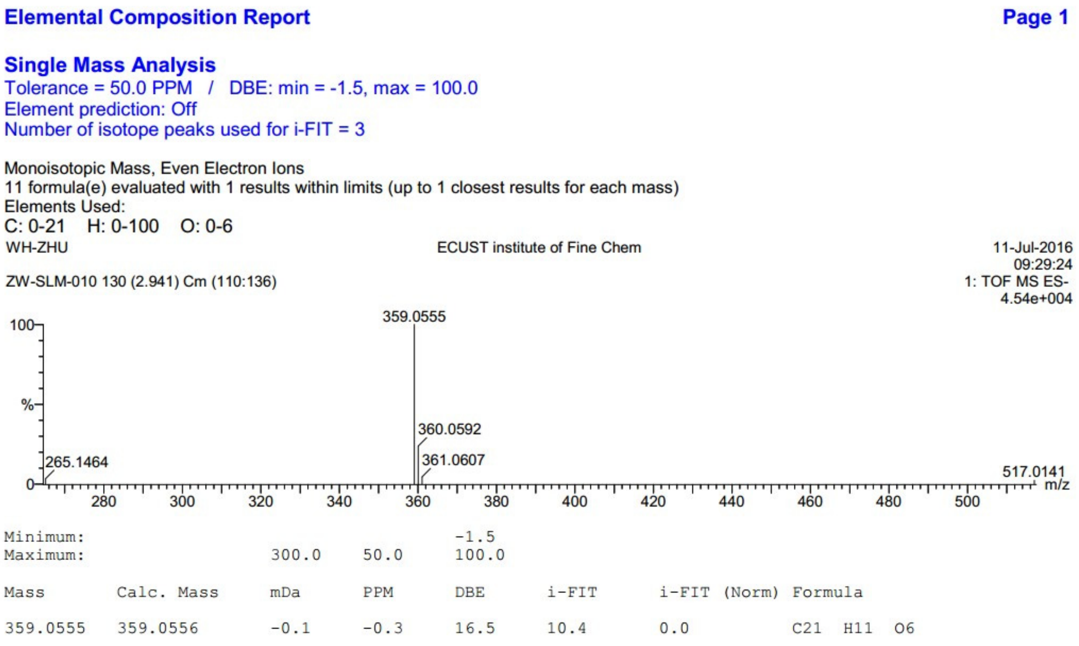

Supplementary Figure 38. HRMS spectrum of F-CHO

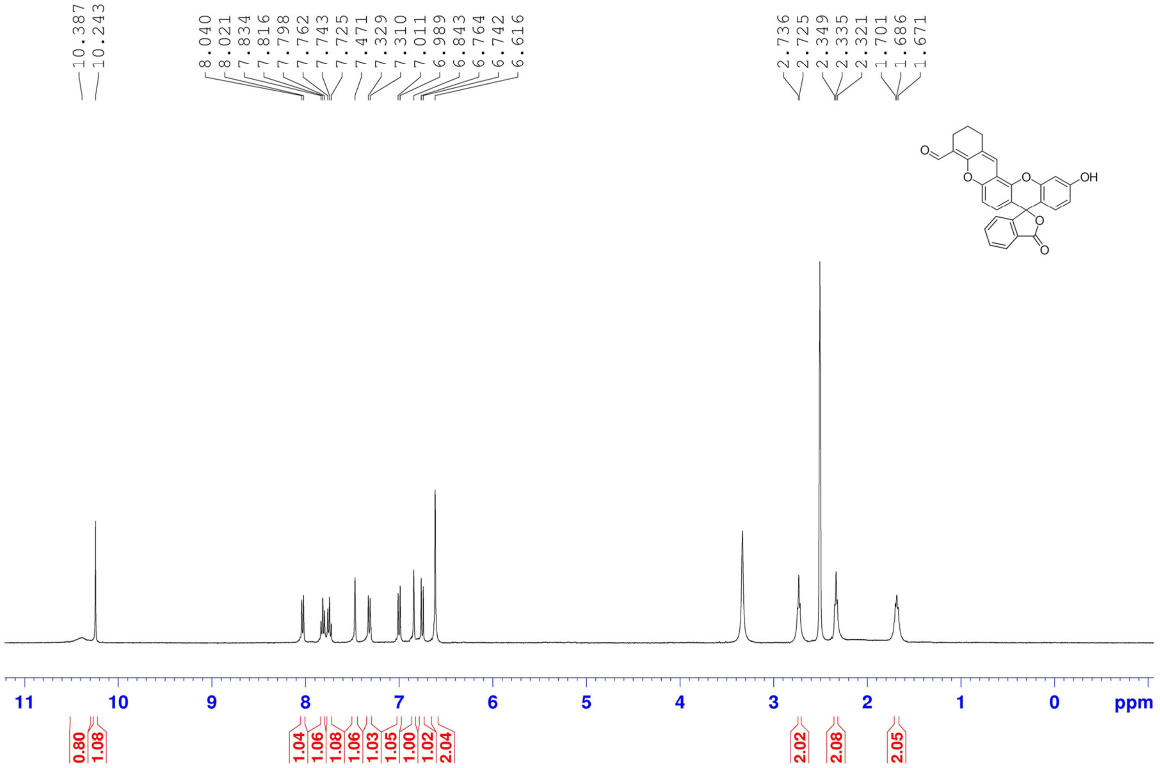

Supplementary Figure 39. ^{1}H NMR spectrum of IFC in DMSO-d_6

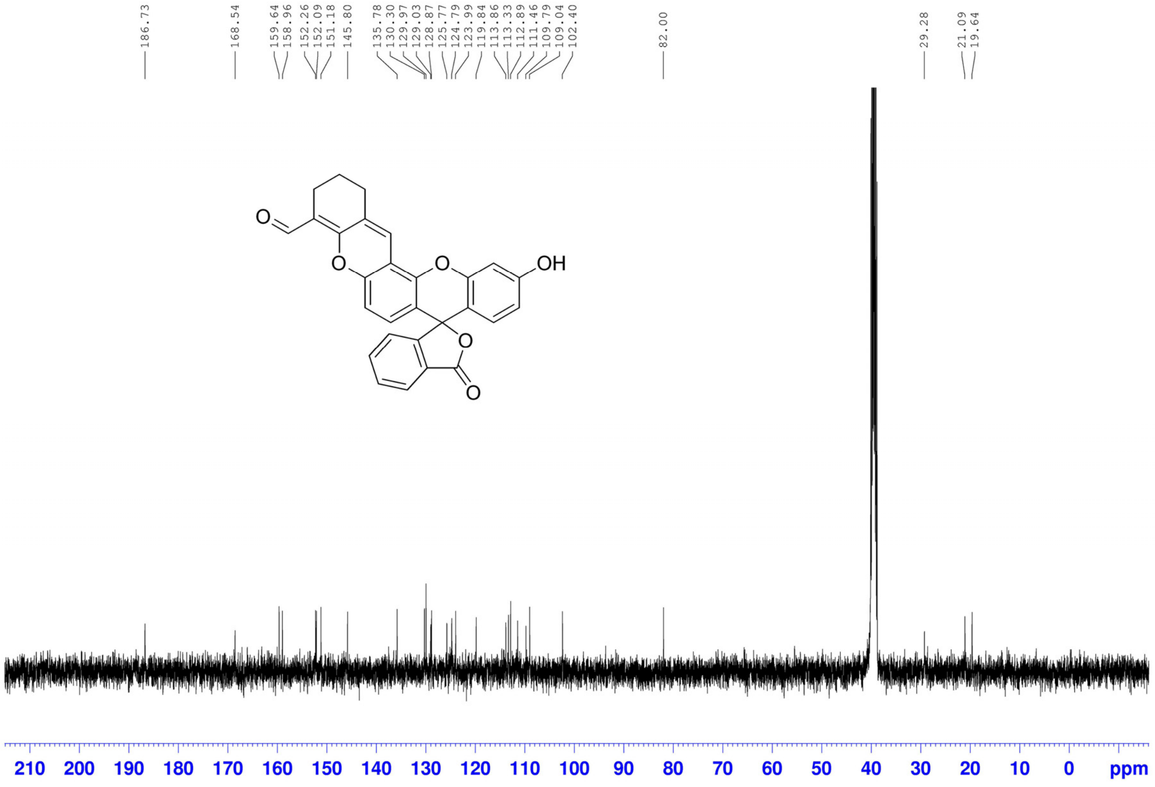

Supplementary Figure 40. ^{13}C NMR spectrum of IFC in DMSO-d_6

Elemental Composition Report **Page 1**

Single Mass Analysis
Tolerance = 50.0 PPM / DBE: min = -1.5, max = 100.0
Element prediction: Off
Number of isotope peaks used for i-FIT = 3

Monoisotopic Mass, Even Electron Ions
6 formula(e) evaluated with 1 results within limits (up to 1 closest results for each mass)
Elements Used:
C: 0-28 H: 0-40 O: 0-6

WH-ZHU ECUST institute of Fine Chem 07-Jun-2016 20:56:53
ZW-SLM-009 3 (0.170) Cm (3:4) 1: TOF MS ES- 6.14e+003

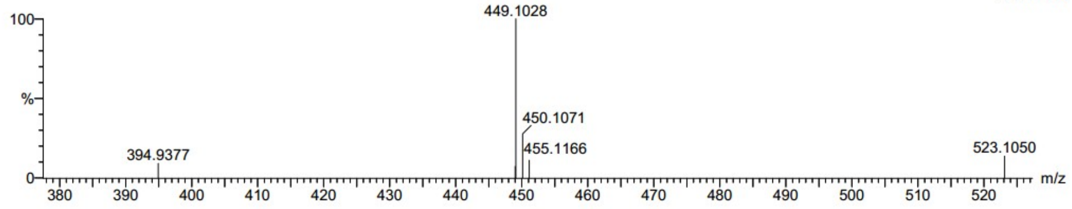

Minimum:				-1.5			
Maximum:		300.0	50.0	100.0			
Mass	Calc. Mass	mDa	PPM	DBE	i-FIT	i-FIT (Norm)	Formula
449.1028	449.1025	0.3	0.7	20.5	203.4	0.0	C28 H17 O6

Supplementary Figure 41. HRMS spectrum of IFC

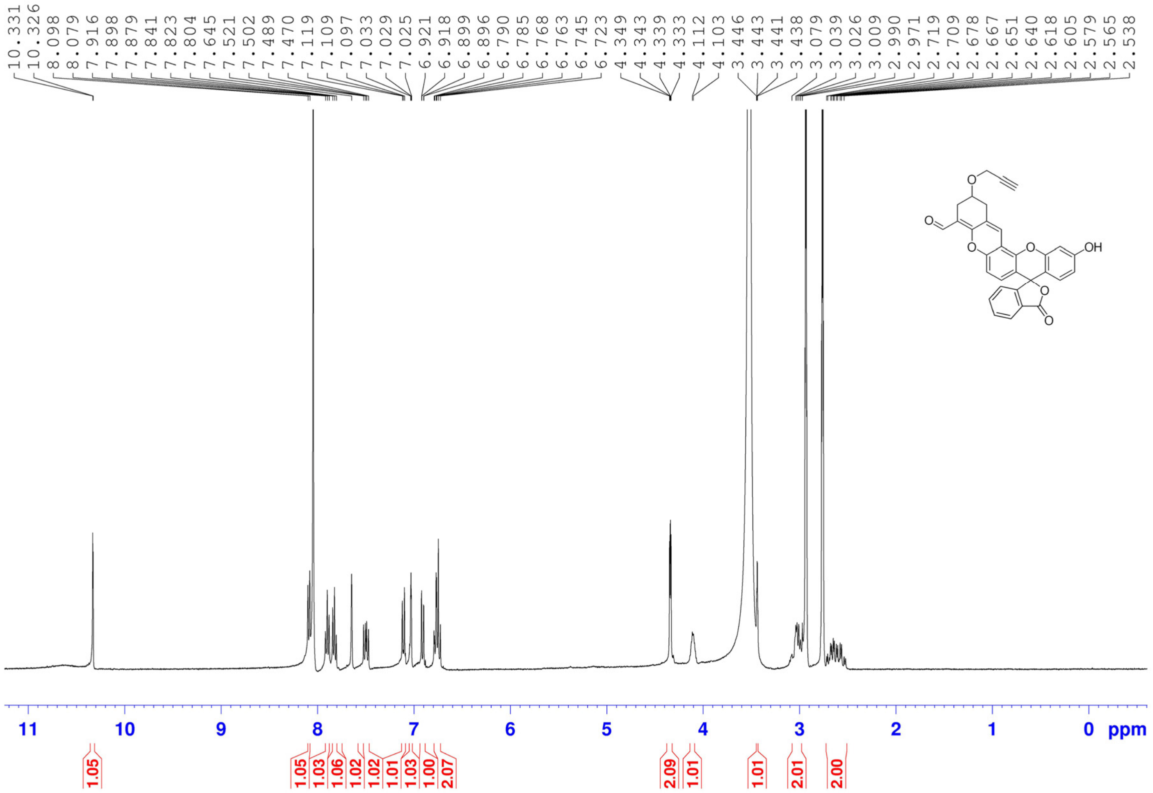

Supplementary Figure 42. ^{1}H NMR spectrum of alkyne-IFC in DMF-d_7

Elemental Composition Report **Page 1**

Single Mass Analysis
Tolerance = 30.0 PPM / DBE: min = -1.5, max = 100.0
Element prediction: Off
Number of isotope peaks used for i-FIT = 2

Monoisotopic Mass, Even Electron Ions
14 formula(e) evaluated with 1 results within limits (up to 1 best isotopic matches for each mass)
Elements Used:
C: 0-31 H: 0-169 O: 0-7

WH-ZHU ECUST institute of Fine Chem 09-Mar-2018 20:43:19

ZW-SLM-1 27 (0.664) Cm (26:28) 1: TOF MS ES- 1.72e+004

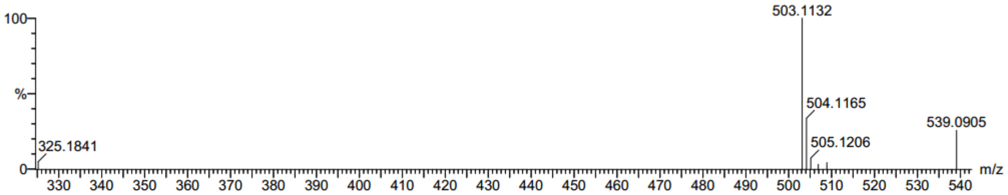

Minimum:				-1.5			
Maximum:		30.0	30.0	100.0			
Mass	Calc. Mass	mDa	PPM	DBE	i-FIT	i-FIT (Norm)	Formula
503.1132	503.1131	0.1	0.2	22.5	9.5	0.0	C31 H19 O7

Supplementary Figure 43. HRMS spectrum of alkyne-IFC

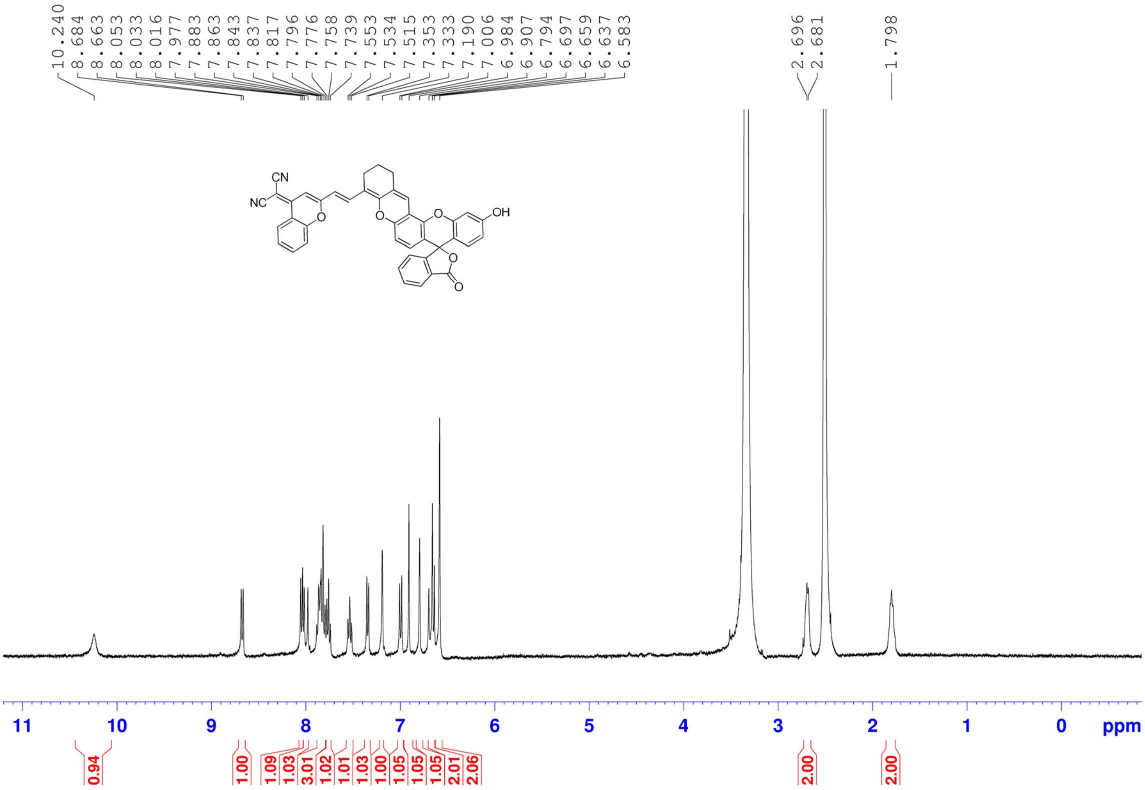

Supplementary Figure 44. ^{1}H NMR spectrum of DCM-IFC in DMSO-d_6

Elemental Composition Report **Page 1**

Single Mass Analysis
Tolerance = 50.0 PPM / DBE: min = -1.5, max = 100.0
Element prediction: Off
Number of isotope peaks used for i-FIT = 3

Monoisotopic Mass, Even Electron Ions
45 formula(e) evaluated with 1 results within limits (up to 1 closest results for each mass)
Elements Used:
C: 0-41 H: 0-100 N: 0-2 O: 0-6

WH-ZHU ECUST institute of Fine Chem 30-Jun-2016 16:19:29
ZW-SLM-011 13 (0.346) Cm (13) 1: TOF MS ES- 2.28e+003

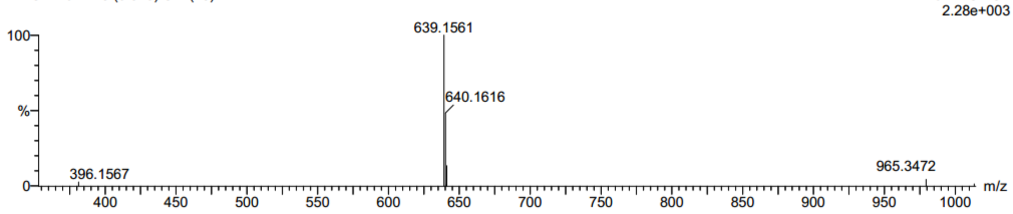

Minimum:				-1.5			
Maximum:		300.0	50.0	100.0			
Mass	Calc. Mass	mDa	PPM	DBE	i-FIT	i-FIT (Norm)	Formula
639.1561	639.1556	0.5	0.8	31.5	21.7	0.0	C41 H23 N2 O6

Supplementary Figure 45. HRMS spectrum of DCM-IFC.

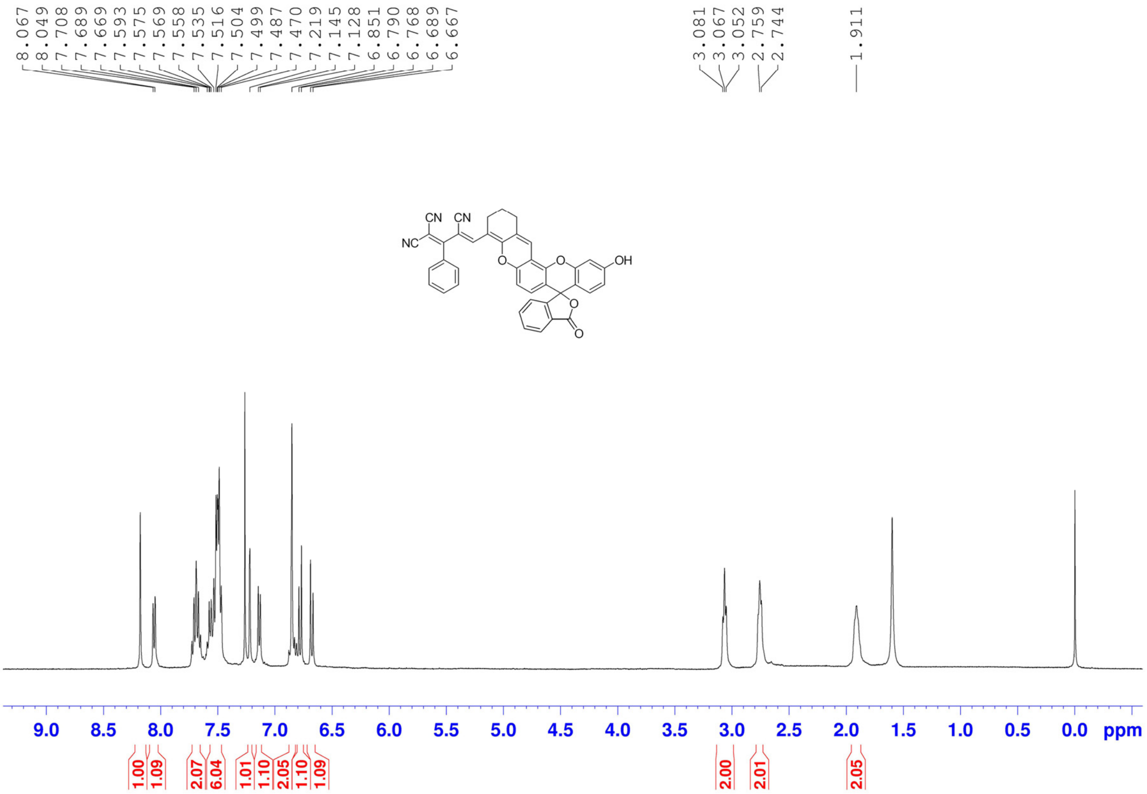

Supplementary Figure 46. ^{1}H NMR spectrum of TCB-IFC in $CDCl_3$

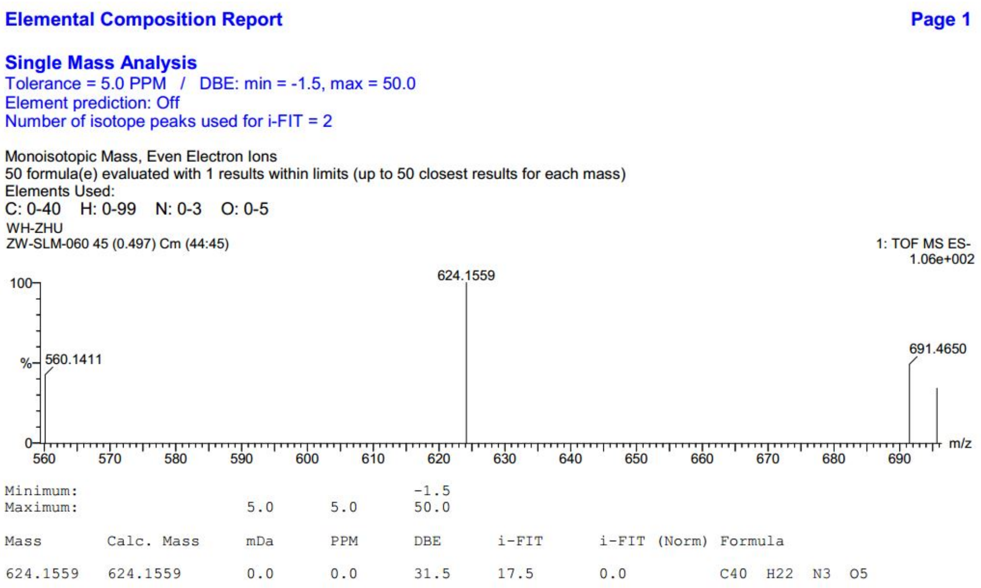

Minimum:				-1.5			
Maximum:		5.0	5.0	50.0			
Mass	Calc. Mass	mDa	PPM	DBE	i-FIT	i-FIT (Norm)	Formula
624.1559	624.1559	0.0	0.0	31.5	17.5	0.0	C40 H22 N3 O5

Supplementary Figure 47. HRMS spectrum of TCB-IFC.

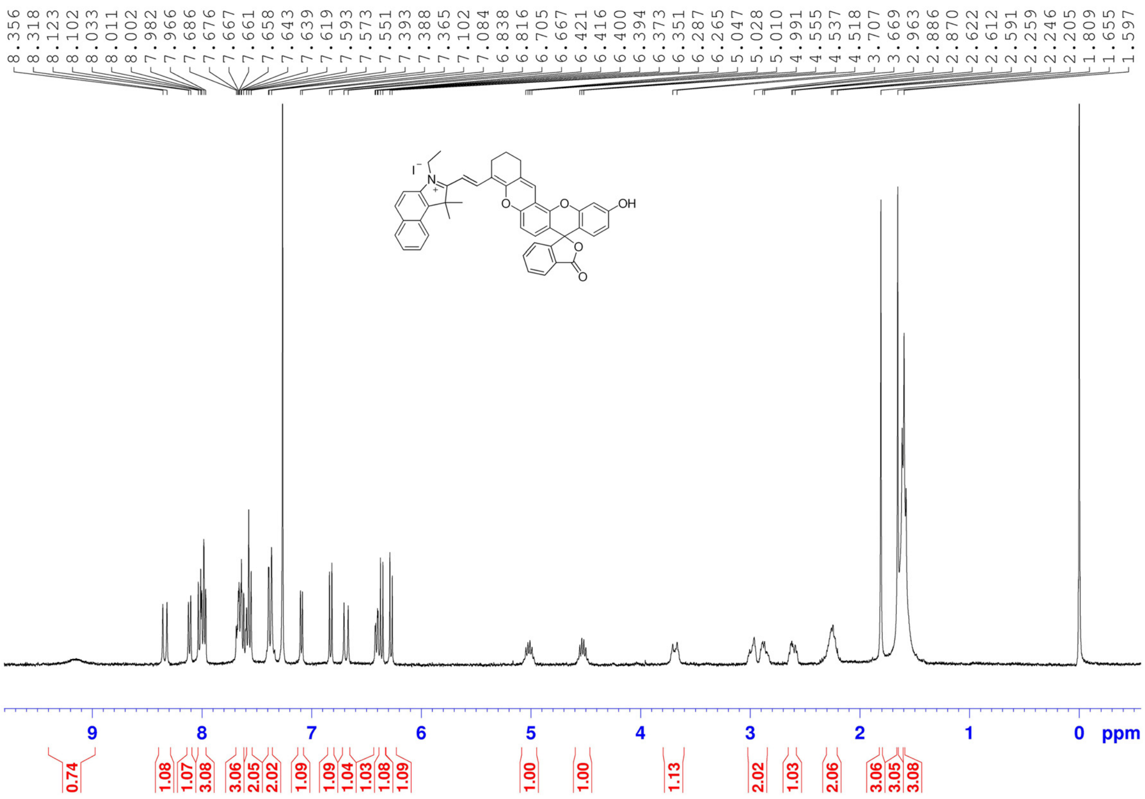

Supplementary Figure 48. ^{1}H NMR spectrum of BI-IFC in $CDCl_3$

Elemental Composition Report Page 1

Single Mass Analysis
Tolerance = 5.0 mDa / DBE: min = -1.5, max = 50.0
Element prediction: Off
Number of isotope peaks used for i-FIT = 2

Monoisotopic Mass, Even Electron Ions
4 formula(e) evaluated with 1 results within limits (up to 50 best isotopic matches for each mass)
Elements Used:
C: 0-45 H: 0-40 N: 0-1 O: 0-5
WH-ZHU
ZW-SLM-041 39 (0.434) Cm (38:39) 1: TOF MS ES+ 1.28e+003

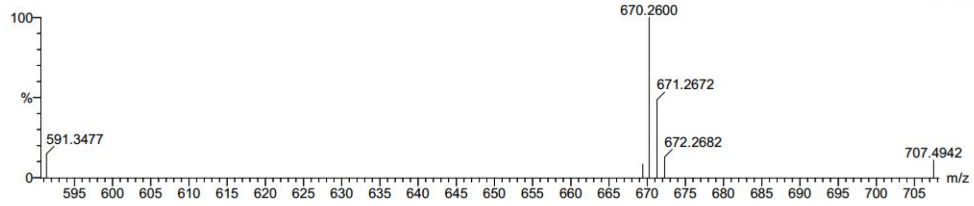

Minimum:				-1.5			
Maximum:		5.0	30.0	50.0			
Mass	Calc. Mass	mDa	PPM	DBE	i-FIT	i-FIT (Norm)	Formula
670.2600	670.2593	0.7	1.0	28.5	10.2	0.0	C45 H36 N O5

Supplementary Figure 49. HRMS spectrum of BI-IFC

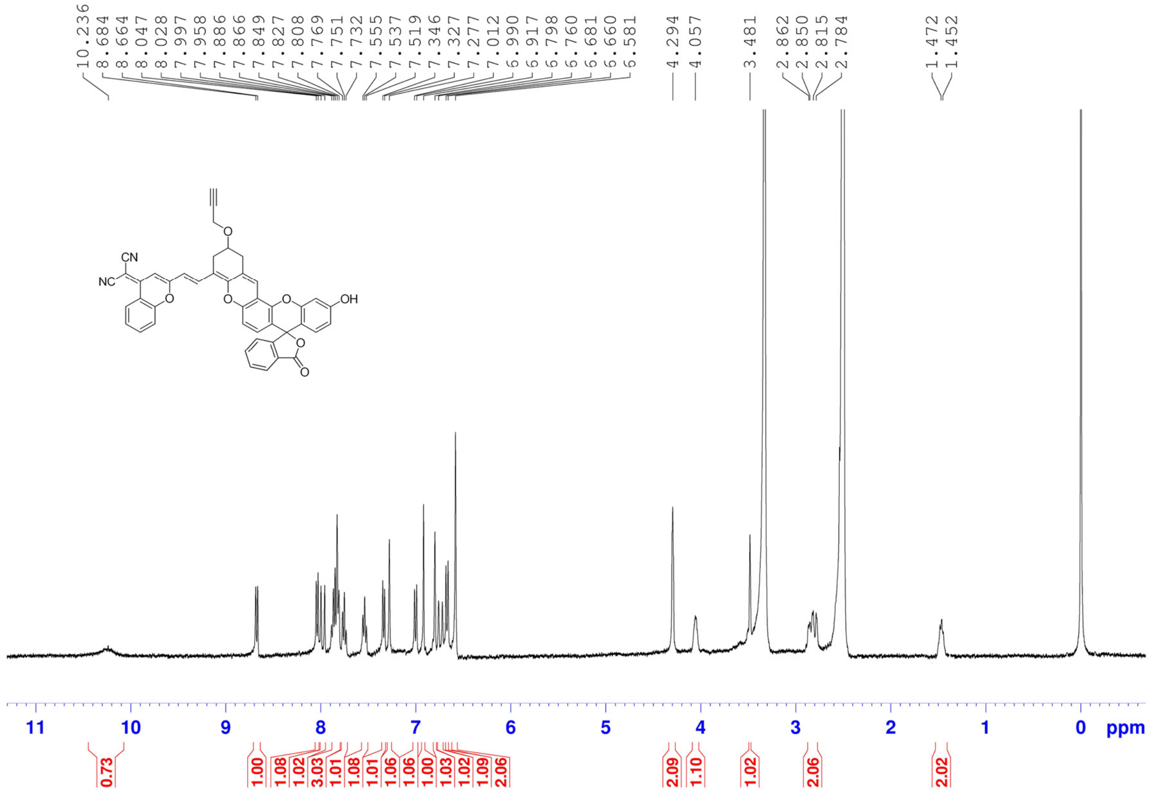

Supplementary Figure 50. ^{1}H NMR spectrum of alkyne-DCM-IFC in DMSO-d_6

Elemental Composition Report Page 1

Single Mass Analysis
Tolerance = 50.0 PPM / DBE: min = -1.5, max = 100.0
Element prediction: Off
Number of isotope peaks used for i-FIT = 3

Monoisotopic Mass, Even Electron Ions
33 formula(e) evaluated with 1 results within limits (up to 1 best isotopic matches for each mass)
Elements Used:
C: 0-44 H: 0-57 N: 0-3 O: 0-7

WH-ZHU ECUST institute of Fine Chem 23-Jun-2017 20:44:26
ZW-SLM-026 32 (0.769) Cm (31:33) 1: TOF MS ES- 2.28e+004

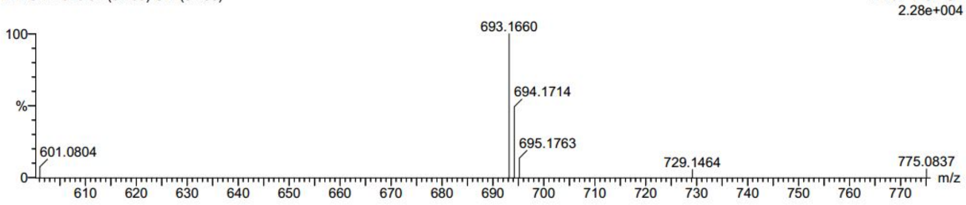

Minimum:				-1.5			
Maximum:		30.0	50.0	100.0			
Mass	Calc. Mass	mDa	PPM	DBE	i-FIT	i-FIT (Norm)	Formula
693.1660	693.1662	-0.2	-0.3	33.5	14.0	0.0	C44 H25 N2 O7

Supplementary Figure 51. HRMS spectrum of alkyne-DCM-IFC

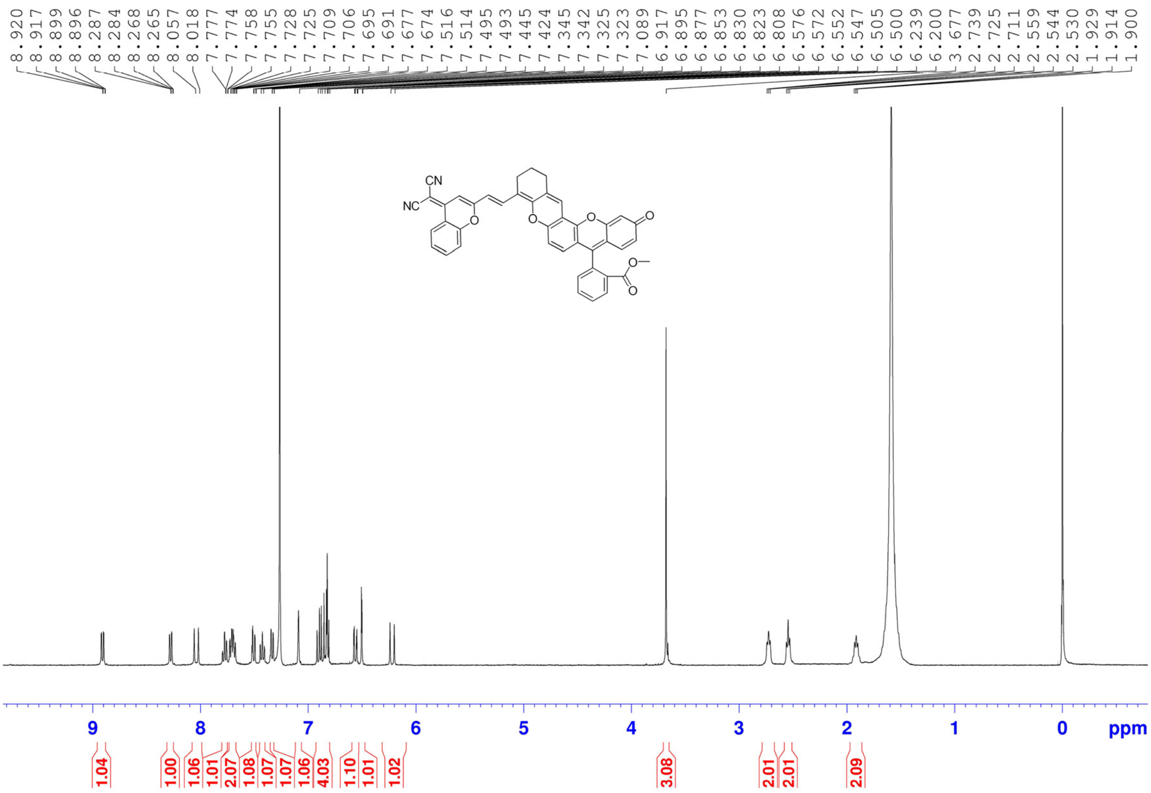

Supplementary Figure 52. ^{1}H NMR spectrum of DCM-IFC-ester in $CDCl_3$

Elemental Composition Report Page 1

Single Mass Analysis
Tolerance = 5.0 PPM / DBE: min = -1.5, max = 50.0
Element prediction: Off
Number of isotope peaks used for i-FIT = 2

Monoisotopic Mass, Even Electron Ions
42 formula(e) evaluated with 1 results within limits (up to 50 closest results for each mass)
Elements Used:
C: 0-42 H: 0-99 N: 0-2 O: 0-6
WH-ZHU
ZW-SLM-62 44 (0.489) Cm (44:45)

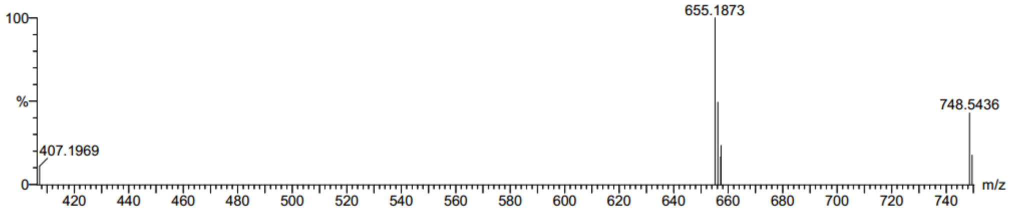

Minimum:				-1.5			
Maximum:		5.0	5.0	50.0			
Mass	Calc. Mass	mDa	PPM	DBE	i-FIT	i-FIT (Norm)	Formula
655.1873	655.1869	0.4	0.6	30.5	7.7	0.0	C42 H27 N2 O6

Supplementary Figure 53. HRMS spectrum of DCM-IFC-ester

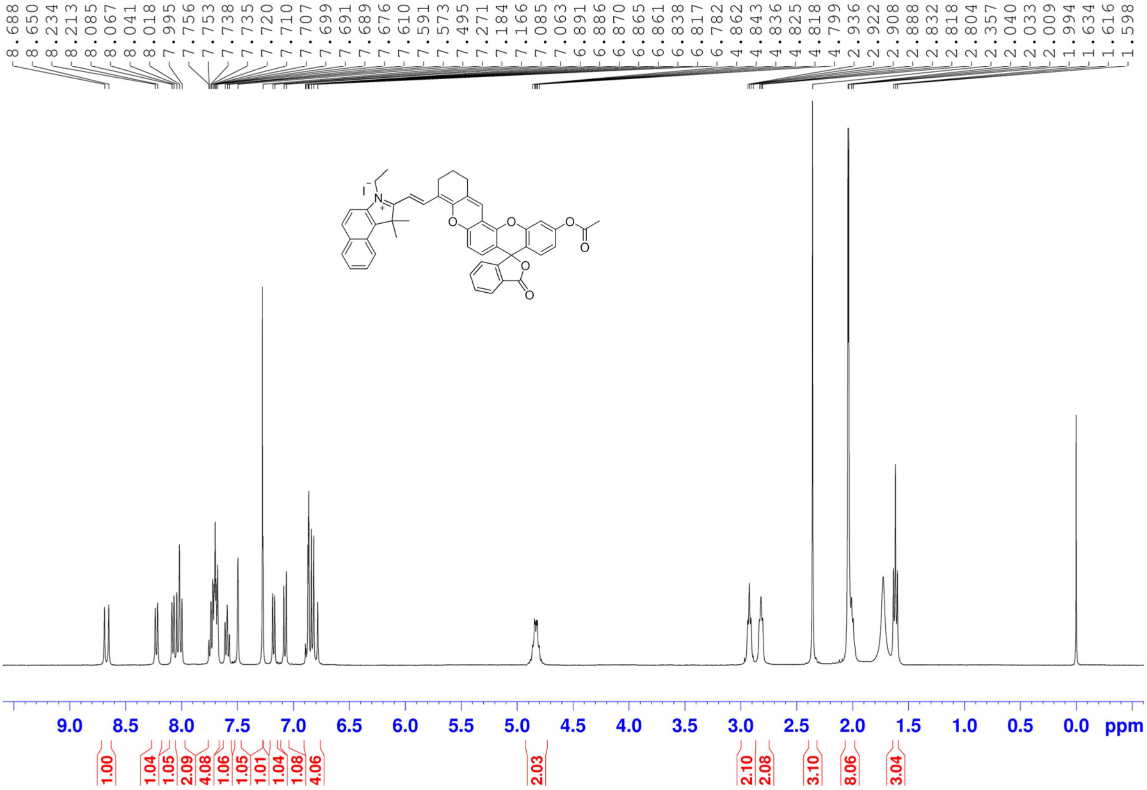

Supplementary Figure 54. ^{1}H NMR spectrum of BI-IFC-ester in $CDCl_3$

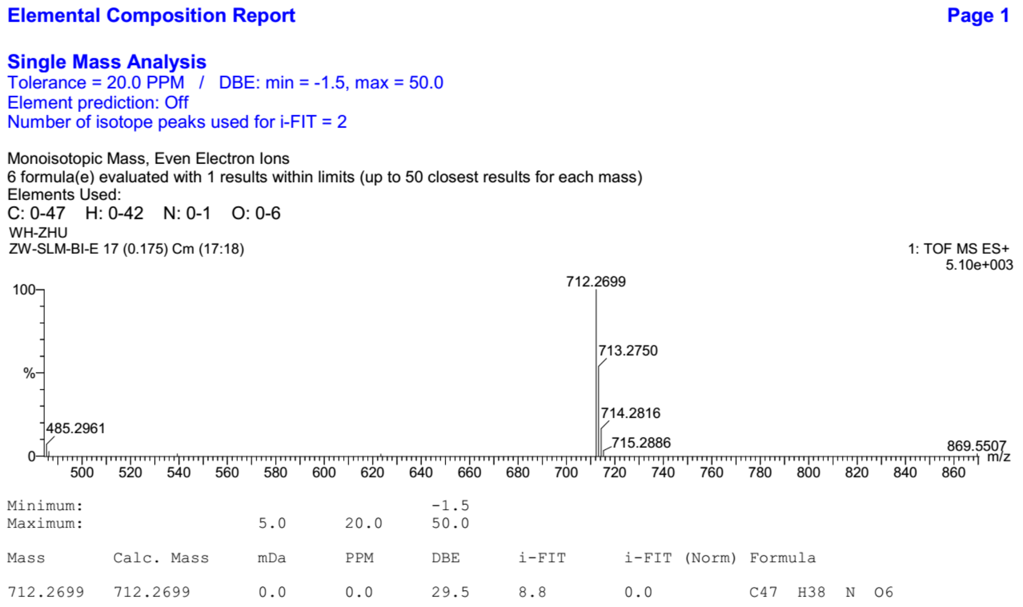

Supplementary Figure 55. HRMS spectrum of BI-IFC-ester

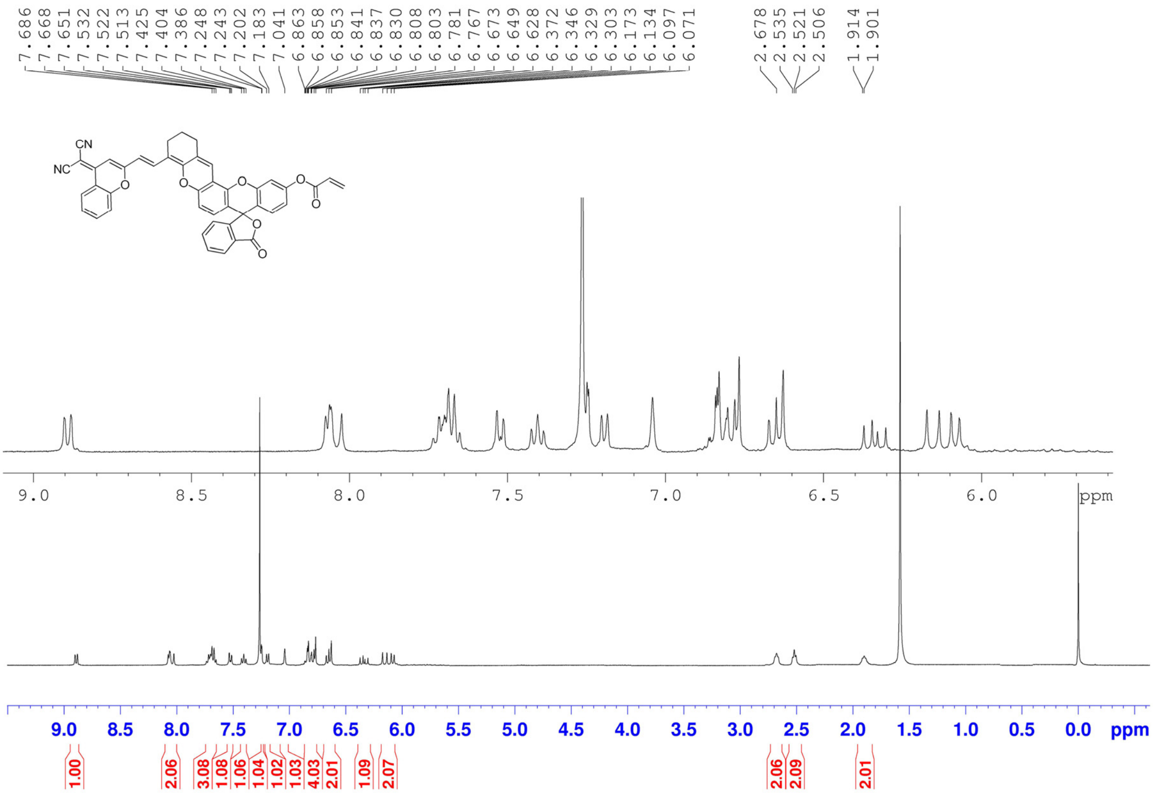

Supplementary Figure 56. ^{1}H NMR spectrum of DCM-IFC-1 in $CDCl_3$

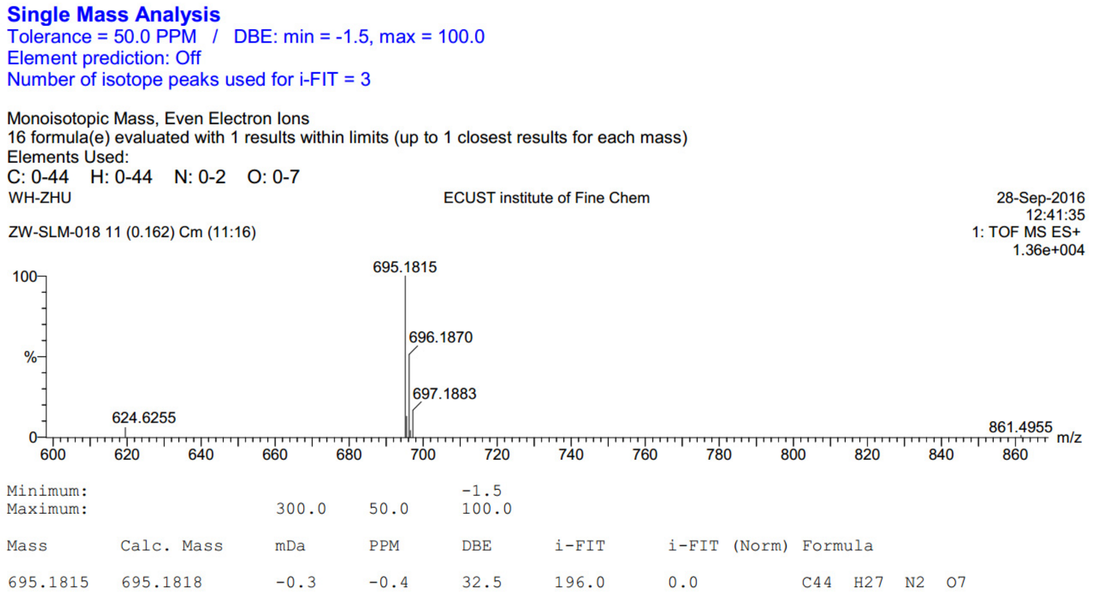

Supplementary Figure 57. HRMS spectrum of DCM-IFC-1

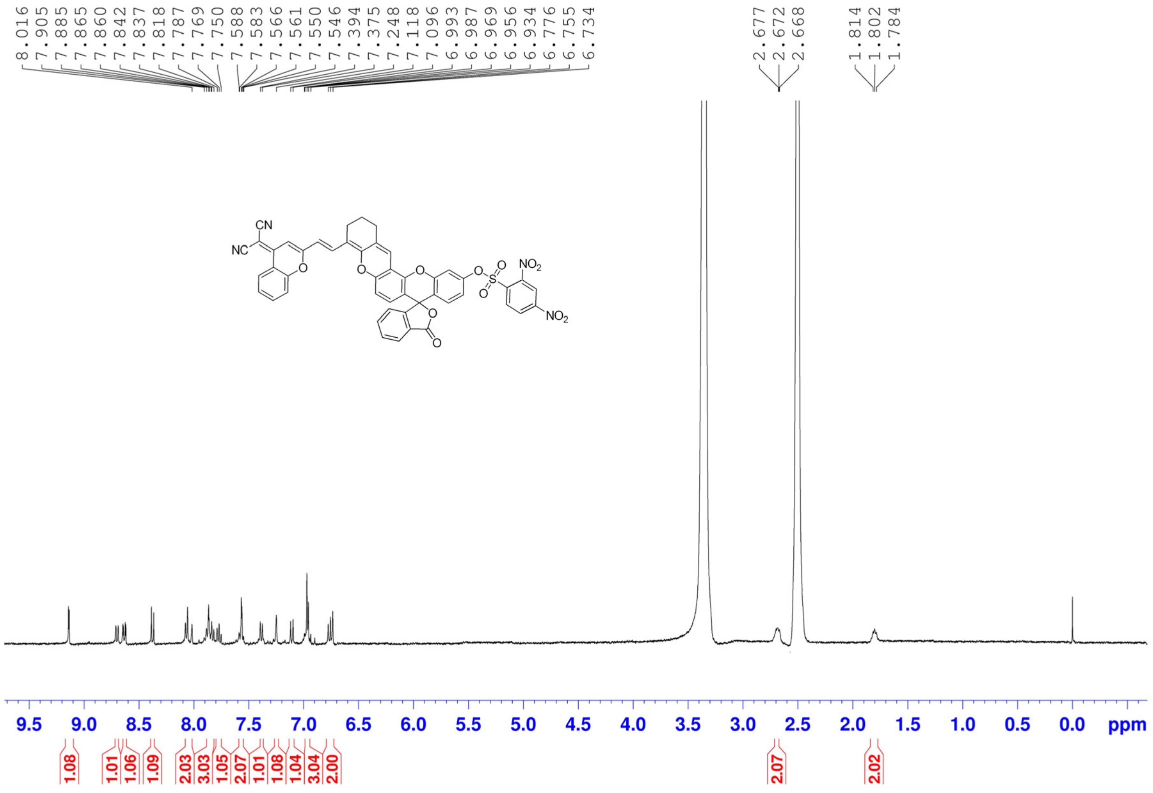

Supplementary Figure 58. ^{1}H NMR spectrum of DCM-IFC-2 in DMSO-d_6

Elemental Composition Report **Page 1**

Single Mass Analysis
Tolerance = 5.0 PPM / DBE: min = -1.5, max = 50.0
Element prediction: Off
Number of isotope peaks used for i-FIT = 2

Monoisotopic Mass, Even Electron Ions
163 formula(e) evaluated with 1 results within limits (up to 50 closest results for each mass)
Elements Used:
C: 0-47 H: 0-99 N: 0-4 O: 0-12 S: 0-1
WH-ZHU
ZW-SLM-061 34 (0.379) Cm (34:37) 1: TOF MS ES+ 2.55e+002

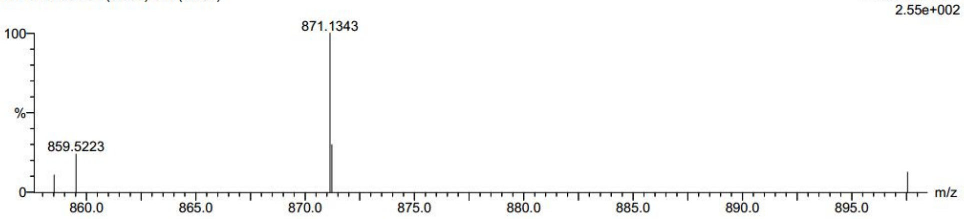

Minimum:				-1.5			
Maximum:		5.0	5.0	50.0			
Mass	Calc. Mass	mDa	PPM	DBE	i-FIT	i-FIT (Norm)	Formula
871.1343	871.1346	-0.3	-0.3	36.5	37.9	0.0	C47 H27 N4 O12 S

Supplementary Figure 59. HRMS spectrum of DCM-IFC-2

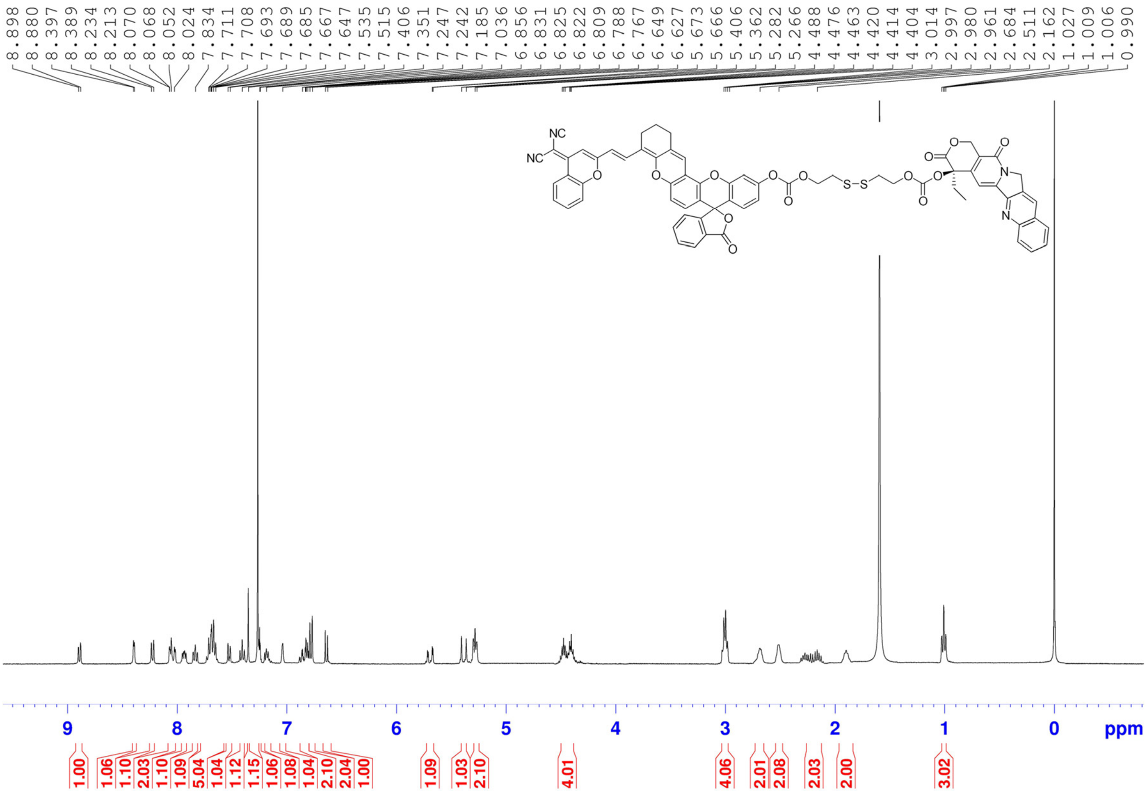

Supplementary Figure 60. ^{1}H NMR spectrum of DCM-IFC-3 in $CDCl_3$

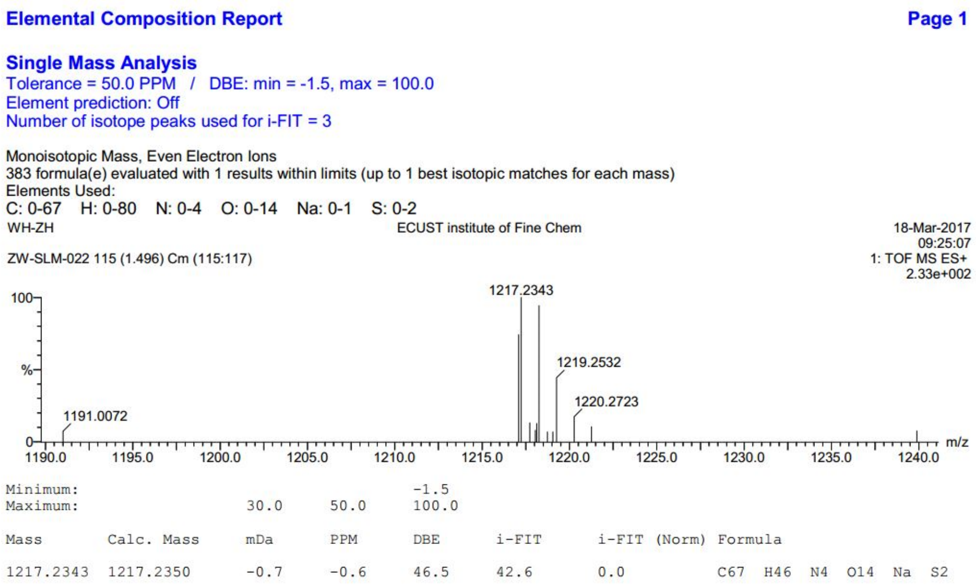

Elemental Composition Report Page 1

Single Mass Analysis
Tolerance = 50.0 PPM / DBE: min = -1.5, max = 100.0
Element prediction: Off
Number of isotope peaks used for i-FIT = 3

Monoisotopic Mass, Even Electron Ions
383 formula(e) evaluated with 1 results within limits (up to 1 best isotopic matches for each mass)
Elements Used:
C: 0-67 H: 0-80 N: 0-4 O: 0-14 Na: 0-1 S: 0-2

WH-ZH ECUST institute of Fine Chem 18-Mar-2017 09:25:07
ZW-SLM-022 115 (1.496) Cm (115:117) 1: TOF MS ES+ 2.33e+002

Minimum:				-1.5			
Maximum:		30.0	50.0	100.0			

Mass	Calc. Mass	mDa	PPM	DBE	i-FIT	i-FIT (Norm)	Formula
1217.2343	1217.2350	-0.7	-0.6	46.5	42.6	0.0	C67 H46 N4 O14 Na S2

Supplementary Figure 61. HRMS spectrum of DCM-IFC-3

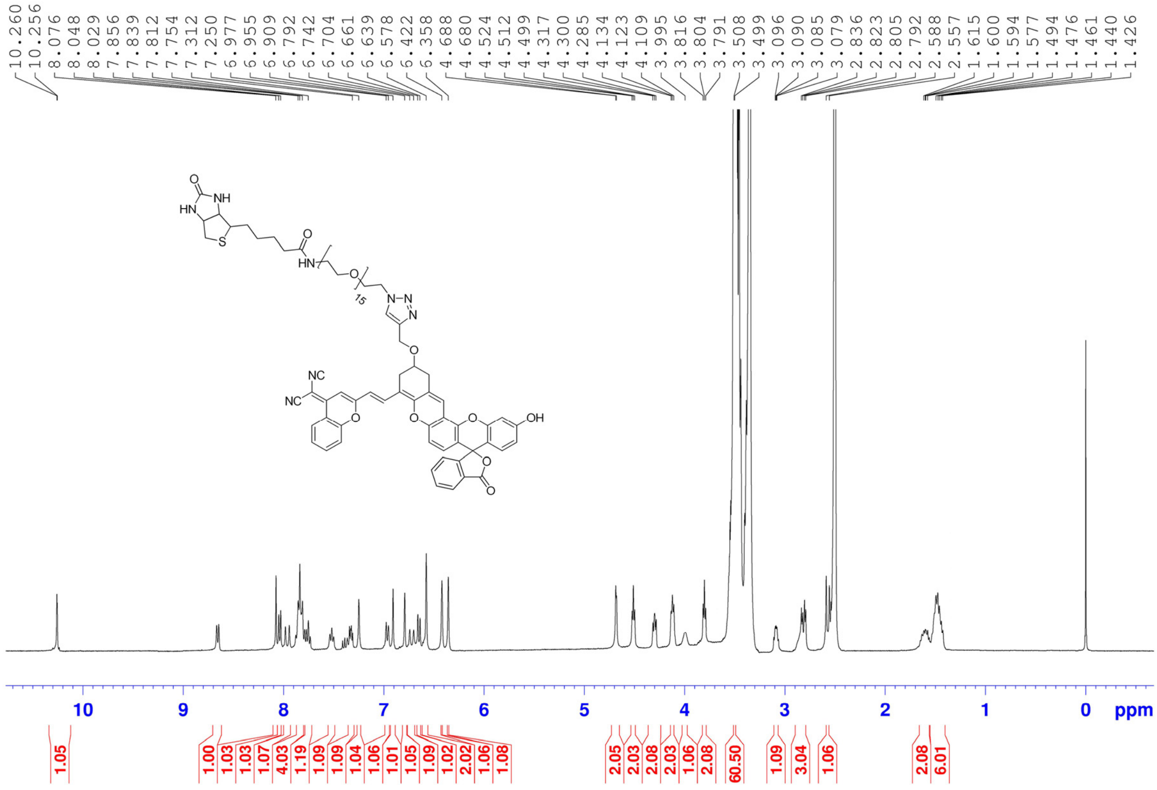

Supplementary Figure 62. ^{1}H NMR spectrum of Biotin-DCM-IFC in DMSO-d_6

Elemental Composition Report **Page 1**

Single Mass Analysis
Tolerance = 20.0 PPM / DBE: min = -1.5, max = 50.0
Element prediction: Off
Number of isotope peaks used for i-FIT = 2

Monoisotopic Mass, Even Electron Ions
439 formula(e) evaluated with 1 results within limits (up to 50 closest results for each mass)
Elements Used:
C: 0-86 H: 0-149 N: 0-8 O: 0-24 S: 0-1
WH-ZHU
ZW-SLM-B-D 71 (1.791) Cm (70:72) 1: TOF MS ES- 6.23e+001

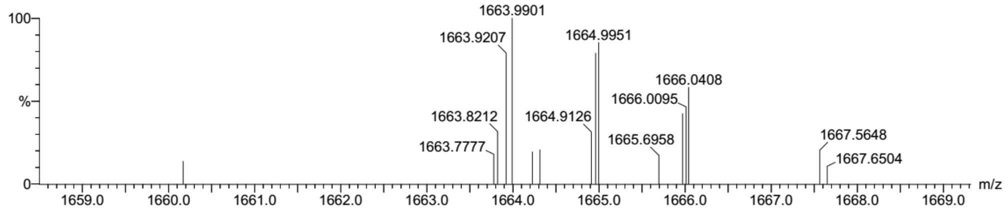

Minimum:				-1.5			
Maximum:		5.0	20.0	50.0			
Mass	Calc. Mass	mDa	PPM	DBE	i-FIT	i-FIT (Norm)	Formula
1665.6958	1665.6962	-0.4	-0.2	38.5	58.5	0.0	C86 H105 N8 O24 S

Supplementary Figure 63. HRMS spectrum of Biotin-DCM-IFC

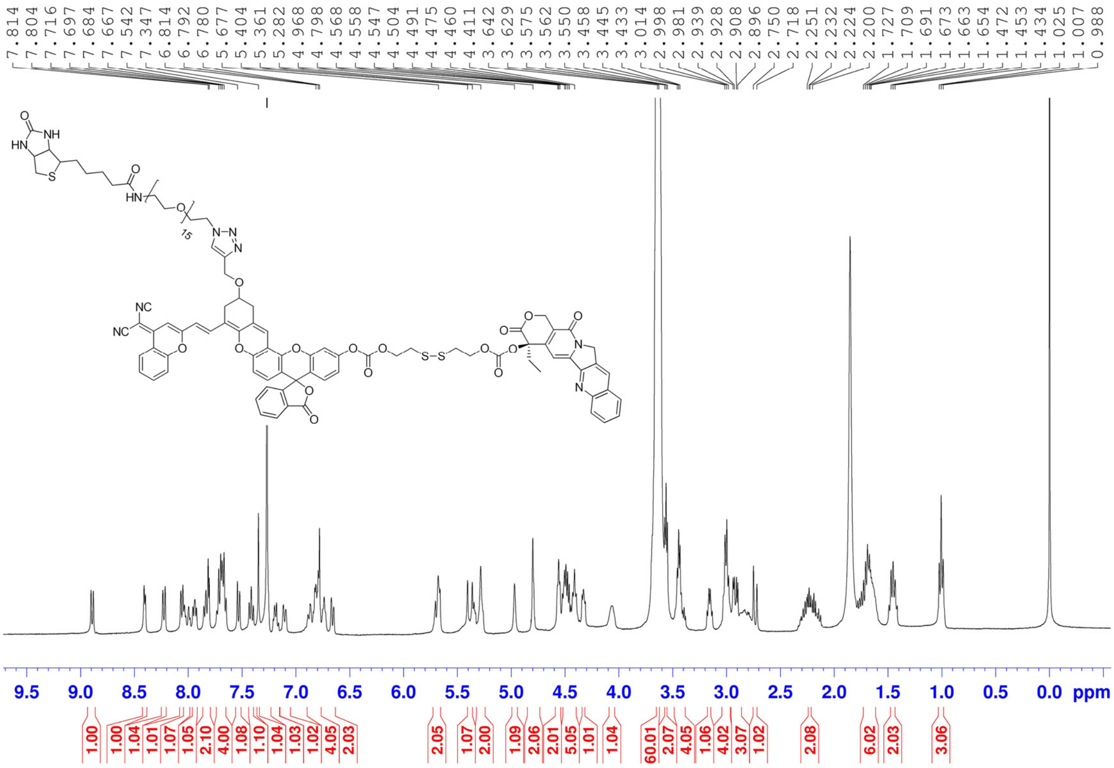

Supplementary Figure 64. ^{1}H NMR spectrum of DCM-IFC-4 in $CDCl_3$

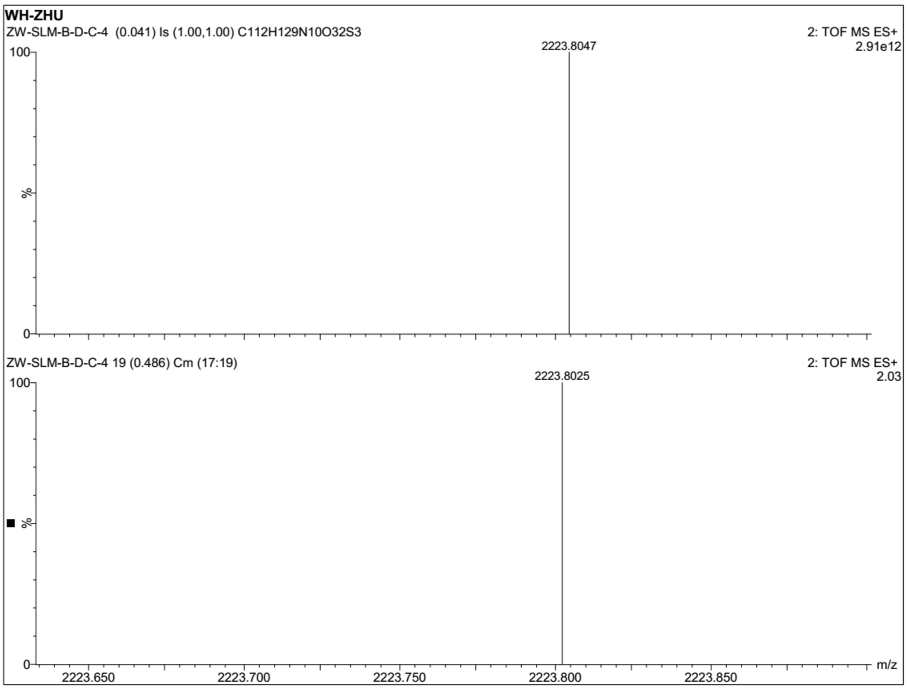

Supplementary Figure 65. HRMS spectrum of DCM-IFC-4. Mass spectrometry (ESI positive ion mode for $[M + H]^+$): calcd for $[C_{112}H_{128}N_{10}O_{32}S_3^+]$: 2223.8047 (above); found: 222.8025 (below)

Supplementary Tables

Supplementary Table 1. Calculated fluorescence properties of open form DCM-IFC from both the S_1 and S_2 states in water.

Compound	Emission State	Major Frontier Molecular Orbital Transitions	f	E_{em} (eV)	λ_{em} (nm)
**DCM-IFC (open form)**	S_1	H←L (96%)	2.1545	1.75	706
	S_2	H←L (44%)<br>H←L+1 (39%)	0.2557	2.49	497

Note: Major frontier molecular orbitals involved during the emission processes (Abbreviations: H for HOMO and L for LUMO), oscillator strength (*f*), de-excitation energy (eV) and emission wavelengths (nm). Computational method: M062X/def2SVP, unless stated otherwise.

Supplementary Table 2. Best-fit parameters of the femtosecond transient absorption of open form DCM-IFC.

	t_1 (ps)	A_1	t_2 (ps)	A_2	t_3 (ps)	A_3
**520 nm**	45.4	-0.00762	1.33	-0.00889	7.379	-0.0104
**760 nm**	22.4	-0.540	1.33	0.00718	0.202	0.0132

Supplementary Table 3. Best-fit parameters of the femtosecond transient absorption of DCM-IFC-ester in dichloromethane.

	t_1 (ps)	A_1	t_2 (ps)	A_2
**520 nm**	47.78 ± 0.40	-0.61	0.66 ± 0.018	-0.39
**770 nm**	62.40 ± 3.97	-0.61	0.96 ± 0.13	0.39

Supplementary Table 4. Calculated fluorescence properties of DCM-IFC-ester from both the S_1 and S_2 states in water.

Compound	Emission State	Major Frontier Molecular Orbital Transitions	*f*	E_{em} (eV)	λ_{em} (nm)
**DCM-IFC-ester** [a]	S_1	H←L (95%)	2.1732	1.76	705
	S_2	H←L (63%)<br>H←L+1 (24%)	0.1781	2.41	515

Note: Major frontier molecular orbitals involved during the emission processes (Abbreviations: H for HOMO and L for LUMO), oscillator strength (*f*), de-excitation energy (eV) and emission wavelengths (nm). Computational method: M062X/def2SVP, unless stated otherwise.

[a] LUMO and LUMO+1 swapped relative position during the optimization of the S_2 state, with respect to the corresponding orbitals as optimized in the S_1 state.

Supplementary Table 5. Calculated fluorescence properties of TCB-IFC (open form) and open form BI-IFC from both the S_1 and S_2 states in water.

Compound	Emission State	Major Frontier Molecular Orbital Transitions	*f*	E_{em} (eV)	λ_{em} (nm)
**TCB-IFC (open form)**	S_1	H←L (95%)	1.6792	1.96	634
	S_2	H-1←L (37%)<br>H-1←L+1 (32%)<br>H←L+1 (28%)	0.3146	2.60	476
**BI-IFC (open form)**	S_1	H←L (93%)	1.7558	1.86	666
	S_2	H-1←L (38%)<br>H-1←L+1 (30%)<br>H←L+1 (27%)	0.3625	2.60	476

Note: Major frontier molecular orbitals involved during the emission processes (Abbreviations: H for HOMO and L for LUMO), oscillator strength (*f*), de-excitation energy (eV) and emission wavelengths (nm). Computational method: M062X/def2SVP, unless stated otherwise.

Supplementary Table 6. Fluorescence quantum yield of DCM-IFC.

**700 nm**	DMSO	Acetonitrile	EtOH	Dichloromethane	Water
$\boldsymbol{\Phi}_{\mathbf{DCM\text{-}IFC}}/\boldsymbol{\Phi}_{\mathbf{DCM\text{-}OH}}$	1.8	0.7	2.4	3.5	0.2

The relative fluorescence quantum yield $\boldsymbol{\Phi}_{\mathrm{DCM\text{-}IFC}}$ value was determined using DCM-OH[5] as a reference.

Supplementary Table 7. Quantification of GSH levels in different cancer cell lines using live imaging based methods. Data with error bars are expressed as mean ± s.d., n = 3. Source data are provided as a Source Data file.

**Cancer Cell Lines**	**A549**	**Hep-G2**	**HeLa**	**HCT116**
**C_{GSH} (mM)**	8.4 ±1.5	7.1 ± 0.2	5.7 ±1.0	7.2 ± 1.8

Supplementary Methods

Synthesis of IFC family chromophores. The intermediate compounds CYC-CHO[1], F-CHO[2], alkyne-CYC[3], BI[3] were synthesized by the established procedures.

Synthesis of CYC-CHO. PBr_3 (26.8 mL, 282.5 mmol) was slowly added to DMF (26 mL, 339 mmol) and $CHCl_3$ (100 mL) at 0 °C. After 60 min, cyclohexanone (CYC) (10 mL, 113 mmol) in solution $CHCl_3$ (15 mL) was added and the mixture was stirred at 25 °C for 16 h. The resulting red solution was then poured onto ice and solid $NaHCO_3$ was slowly added until pH ~ 7. The layers were separated and the aqueous layer was extracted with CH_2Cl_2 (100 mL). The organic layer was dried over anhydrous Na_2SO_4, filtered and concentrated by evaporation. The chemical purity of the crude yellow oil was good and could be used directly in the next step without further purification. CYC-CHO could be stored for months under N_2 below -20 °C.

Synthesis of F-CHO. Fluorescein (4 g, 12 mmol), $CHCl_3$ (10 mL), MeOH (6 mL) and 15-crown-5 (0.06 g, 0.27mmol) were placed in a 100 mL flask. Then 50% NaOH solution (20 g) are carefully added while the reaction temperature is maintained at 0 ºC. The mixture was stirred at 55 ºC for 5 h. After cooling, the mixture was acidified with 10 M H_2SO_4. The precipitates were collected and dried in vacuo. Then the crude product was purified by silica gel chromatography (CH_2Cl_2/MeOH 100:1) to afford F-CHO as pale yellow solid (500 mg): Yield 12%. ^{1}H-NMR (400 MHz, DMSO-d_6, ppm): δ 6.62 (s, 1H), 6.62 (d, J = 1.24 Hz, 1H), 6.72 (d, J = 8.92 Hz, 1H), 6.85 (d, J = 1.24 Hz, 1H), 6.96 (d, J = 8.92 Hz, 1H), 7.32 (d, J = 7.52 Hz, 1H), 7.74 (t, J = 7.52 Hz, 1H), 7.82 (t, J = 7.52 Hz, 1H), 8.02 (d, J = 7.52 Hz, 1H), 10.26 (s, 1H, -O*H*), 10.64 (s, 1H, -C*H*O), 11.89 (s, 1H, -O*H*). ^{13}C-NMR (100 MHz, DMSO-d_6, ppm): δ = 81.78, 102.61, 109.09, 109.16, 109.64, 113.35, 113.49, 123.97, 124.77, 125.87, 129.00, 130.30, 135.78, 136.52, 150.82, 152.15, 152.34, 159.59, 162.91, 168.52, 192.84. Mass spectrometry (ESI negative ion mode for $[M - H]^-$): calcd for $[C_{21}H_{11}O_6^-]$: 359.0556; found: 359.0555.

Synthesis of IFC. F-CHO (500 mg, 1.39 mmol), CYC-CHO (523 mg, 2.78 mmol) and Cs_2CO_3 (1.4 g, 4.17 mmol) were dissolved in anhydrous DMF (12 mL). The mixture was stirred at 25 °C for 48 h to reveal an intense yellow spot. The precipitates were collected and dried in vacuo. Then the crude washed with deionized H_2O (25 mL) and the insoluble substances were then filtered. The resulting residue was dissolved in CH_2Cl_2 (100 mL) and washed with deionized H_2O (2 × 25 mL). The organic layer was dried over anhydrous Na_2SO_4, filtered and concentrated by evaporation. Purification on silica gel (CH_2Cl_2/MeOH 100:1) provided the desired IFC (125 mg, 0.28 mmol) as deep yellow solid in 20% yield. ^{1}H-NMR (400 MHz, DMSO-d_6, ppm): δ 1.69 (m, 2H, -CH_2), 2.33 (m, 2H, -CH_2), 2.73 (m, 2H, -CH_2), 6.62 (s, 2H, Ph-H), 6.75 (d, J = 8.80 Hz, 1H, Ph-H), 6.84 (s, 1H, Ph-H), 7.00 (d, J = 8.80 Hz, 1H, Ph-H), 7.32 (d, J = 7.56 Hz, 1H, Ph-H), 7.47 (s, 1H, alkene-H), 7.74 (t, J = 7.56 Hz, 1H, Ph-H), 7.82 (t, J = 7.56 Hz, 1H, Ph-H), 8.03 (d, J = 7.56 Hz, 1H, Ph-H), 10.24 (s, 1H, -C*H*O), 10.39 (s, 1H, -O*H*). ^{13}C-NMR (100 MHz, DMSO-d_6, ppm): δ = 19.64, 21.09, 29.28, 82.00, 102.40, 109.04, 109.79, 111.46, 112.89, 113.33, 113.86, 119.84, 123.99, 124.79, 125.77, 128.87, 129.03, 129.97, 130.30, 135.78, 145.80, 151.18, 152.09, 152.26, 158.96, 159.64, 168.54, 186.73. Mass spectrometry (ESI negative ion mode for $[M - H]^-$): calcd for $[C_{28}H_{17}O_6^-]$: 449.1025; found: 449.1028.

Synthesis of alkyne-CYC-CHO. PBr_3 (7 mL, 70 mmol) was slowly added to DMF (5 mL, 85 mmol) and $CHCl_3$ (25 mL) at 0 °C. After 60 min, alkyne-CYC (3 g, 20 mmol) in solution $CHCl_3$ (5 mL) was added and the mixture was stirred at 25 °C for 16 h. The resulting red solution was then poured onto ice and solid $NaHCO_3$ was slowly added until pH ~ 7. The layers were separated and the aqueous layer was extracted with CH_2Cl_2 (100 mL). The organic layer was dried over anhydrous Na_2SO_4, filtered and concentrated by evaporation. The chemical purity of the crude yellow oil was good and could be used directly in the next step without further purification. alkyne-CYC-**CHO** could be stored for months under N_2 below -20 °C.

Synthesis of alkyne-IFC. F-CHO (500 mg, 1.39 mmol), alkyne-CYC-CHO (672 mg, 2.78 mmol) and Cs_2CO_3 (1.4 g, 4.17 mmol) were dissolved in anhydrous DMF (12 mL). The mixture was stirred at 25 °C for 48 h to reveal an intense yellow spot. The precipitates were collected and dried in vacuo. Then the crude washed with deionized H_2O (25 mL) and the insoluble substances were then filtered. The resulting residue was dissolved in CH_2Cl_2 (100 mL) and washed with deionized H_2O (2 × 25 mL). The organic layer was dried over anhydrous Na_2SO_4, filtered and concentrated by evaporation. Purification on silica gel (CH_2Cl_2/MeOH 100:1) provided the desired alkyne-IFC (105 mg, 0.21 mmol) as deep yellow solid in 15% yield. ^{1}H-NMR (400 MHz, DMF-d_7, ppm): δ 2.52 - 2.72 (m, 2H, -CH_2), 2.97 - 3.10 (m, 2H, -CH_2), 3.44 (m, 1H, alkyne-H), 4.10 (m, 1H, -O*CH*), 4.34 (m, 2H, -CH_2), 6.78 (m, 2H, Ph-H), 6.91 (d, *J* = 8.80 Hz, 1H, Ph-H), 7.03 (s, *J* = 1.72 Hz, 1H, Ph-H), 7.11 (d, *J* = 8.80 Hz, 1H, Ph-H), 7.50 (m, 1H, Ph-H), 7.65 (s, 1H, alkene-H), 7.82 (t, *J* = 7.64 Hz, 1H, Ph-H), 7.90 (t, *J* = 7.64 Hz, 1H, Ph-H), 8.09 (d, *J* = 7.64 Hz, 1H, Ph-H), 10.33 (s, 1H, -C*H*O). Mass spectrometry (ESI negative ion mode for $[M - H]^-$): calcd for $[C_{31}H_{19}O_7^-]$: 503.1131; found: 503.1132.

Synthesis of DCM-IFC. DCM (89 mg, 0.43 mmol) and IFC (130 mg, 0.29 mmol) were dissolved in toluene (40 mL) along with acetic acid (0.5 mL) and piperidine (1.0 mL), the system was under argon protection and then refluxed for 10 h. Toluene was removed by evaporation, and the residue was purified by silica gel chromatography (CH_2Cl_2/MeOH 100:1) to get the desired product DCM-IFC (60 mg, 0.094 mmol), a dark purple solid. Yield was 32%. ^{1}H-NMR (400 MHz, DMSO-d_6, ppm): δ 1.80 (m, 2H, -CH_2), 2.69 (m, 2H, -CH_2), 6.58 (s, 2H, Ph-H), 6.65 (d, 1H, *J* = 8.64 Hz, Ph-H), 6.68 (d, *J* = 15.12 Hz, 1H, alkene-H), 6.79 (s, 1H, Ph-H), 6.91 (s, 1H, alkene-H), 6.99 (d, *J* = 8.64 Hz, 1H, Ph-H), 7.19 (s, 1H, alkene-H), 7.34 (d, *J* = 7.76 Hz, 1H, Ph-H), 7.53 (t, *J* = 7.76 Hz, 1H, Ph-H), 7.76 (t, *J* = 7.76 Hz, 1H, Ph-H), 7.80 – 7.88 (m, 3H, Ph-H),8.00 (d, *J* = 15.12 Hz, 1H, alkene-H), 8.04 (d, *J* = 7.76 Hz, 1H, Ph-H), 8.67 (d, *J* = 8.40 Hz, 1H, Ph-H), 10.24 (s, 1H, -O*H*). Mass spectrometry (ESI negative ion mode for $[M - H]^-$): calcd for $[C_{41}H_{23}N_2O_6^-]$: 639.1556; found: 639.1561.

Synthesis of TCB-IFC. TCB (42 mg, 0.22 mmol) and IFC (50 mg, 0.11 mmol) were dissolved in acetic anhydride (20 mL), the system was under argon protection and then refluxed for 3 h. Acetic anhydride was removed by evaporation, and the residue was purified by silica gel chromatography (CH_2Cl_2/MeOH 200:1) to get the desired product TCB-IFC (20 mg, 0.032 mmol), a dark purple solid. Yield was 30%. ^{1}H-NMR (400 MHz, $CDCl_3$, ppm): δ 1.83 (m, 2H, -CH_2), 2.80 (m, 2H, -CH_2), 2.93 (m, 2H, -CH_2), 6.63 (s, 1H), 6.63 (s, 1H), 6.69 (d, *J* = 8.92 Hz, 1H),

6.86 - 6.92 (m, 2H), 7.29 (d, *J* = 7.56 Hz, 1H), 7.57 - 7.66 (m, 5H), 7.75 (t, *J* = 7.06 Hz, 1H), 7.81 (t, *J* = 7.44 Hz, 1H), 7.84 (s, 1H), 8.04 (m, 2H), 10.32 (s, 1H, -O*H*) Mass spectrometry (ESI negative ion mode for $[M - H]^-$): calcd for $[C_{40}H_{22}N_3O_5^-]$: 624.1559; found: 624.1559.

Synthesis of BI-IFC. BI (162 mg, 0.44 mmol) and IFC (100 mg, 0.22 mmol) were dissolved in toluene (40 mL) along with acetic acid (0.5 mL) and piperidine (1.0 mL), the system was under argon protection and then refluxed for 10 h. Toluene was removed by evaporation, and the residue was purified by silica gel chromatography (CH_2Cl_2/MeOH 100:1) to get the desired product BI-IFC (30 mg, 0.045 mmol), a dark mazarine solid. Yield was 20%. ^{1}H-NMR (400 MHz, $CDCl_3$, ppm): δ 1.60 (m, 3H, $-CH_2CH_3$), 1.66 (s, 3H, $-CH_3$), 1.81 (s, 3H, $-CH_3$), 2.26 (m, 2H, $-CH_2$), 2.61 (m, 1H, $-CH_2$), 2.93 (m, 2H, $-CH_2$), 3.69 (m, 1H, $-CH_2$), 4.50 - 4.56 (m, 1H, $-CH_2CH_3$), 4.98 - 5.05 (m, 1H, $-CH_2CH_3$), 6.28 (d, *J* = 8.60 Hz, 1H, Ph-H), 6.36 (d, *J* = 8.84 Hz, 1H, Ph-H), 6.41 (d, *J* = 8.60 Hz, 1H, Ph-H), 6.69 (d, *J* = 15.28 Hz, 1H, alkene-H), 6.83 (d, *J* = 8.88 Hz, 1H, Ph-H), 7.09 (d, *J* = 7.16 Hz, 1H, Ph-H), 7.38 (m, 2H), 7.55 - 7.59 (m, 2H), 7.62 - 7.69 (m, 3H), 8.00 – 8.03 (m, 3H), 8.11 (d, *J* = 8.48 Hz, 1H, Ph-H), 8.34 (d, *J* = 15.28 Hz, 1H, alkene-H), 9.15 (s, 1H, -O*H*). Mass spectrometry (ESI positive ion mode for $[M]^+$): calcd for $[C_{45}H_{36}NO_5^+]$: 670.2593; found: 670.2600.

Synthesis of alkyne-DCM-IFC. DCM (55 mg, 0.26 mmol) and alkyne-IFC (60 mg, 0.12 mmol) were dissolved in toluene (40 mL) along with acetic acid (0.5 mL) and piperidine (1.0 mL), the system was under argon protection and then refluxed for 10 h. Toluene was removed by evaporation, and the residue was purified by silica gel chromatography (CH_2Cl_2/MeOH 100:1) to get the desired product alkyne-DCM-IFC (20 mg, 0.028 mmol), a dark purple solid. Yield was 23%. ^{1}H-NMR (400 MHz, DMSO-d_6, ppm): δ 1.46 (m, 2H, $-CH_2$), 2.82 (m, 2H, $-CH_2$), 3.48 (s, 1H, alkyne-H), 4.06 (m, 1H, -O*CH*), 4.29 (m, 2H, $-OCH_2$), 6.58 (s, 2H, Ph-H), 6.67 (d, *J* = 8.72 Hz, 1H, Ph-H), 6.74 (d, *J* = 15.96 Hz, 1H, alkene-H), 6.80 (s, 1H, Ph-H), 6.92 (s, 1H, alkene-H), 7.00 (d, *J* = 8.72 Hz, 1H, Ph-H), 7.28 (s, 1H, alkene-H), 7.34 (d, *J* = 7.76 Hz, 1H, Ph-H), 7.54 (t, *J* = 7.76 Hz, 1H, Ph-H), 7.75 (t, *J* = 7.76 Hz, 1H, Ph-H), 7.81 - 7.89 (m, 3H, Ph-H), 7.98 (d, *J* = 15.96 Hz, 1H, alkene-H), 8.04 (d, *J* = 7.76 Hz, 1H, Ph-H), 8.67 (d, *J* = 8.28 Hz, 1H, Ph-H), 10.24 (s, 1H, -O*H*). Mass spectrometry (ESI negative ion mode for $[M - H]^-$): calcd for $[C_{44}H_{25}N_2O_7^-]$: 693.1662; found: 693.1660.

Synthesis of DCM-IFC-ester. DCM-IFC (30 mg, 0.047 mmol) was dissolved in chloroform (30 mL) along with methanol (30 mL) and concentrated sulfuric acid (98%) (0.01 mL), the system was under argon protection and then refluxed for 10 h. Chloroform and methanol was removed by evaporation, and the residue was purified by silica gel chromatography (CH_2Cl_2/MeOH 100:1) to get the desired product DCM-IFC-ester (5 mg, 0.0074 mmol), a dark purple solid. Yield was 15%. ^{1}H-NMR (400 MHz, DMSO-d_6, ppm): δ 1.91 (m, 2H, $-CH_2$), 2.54 (m, 2H, $-CH_2$), 2.73 (m, 2H, $-CH_2$), 3.68 (s, 3H, $-CH_3$), 6.22 (d, *J* = 15.60 Hz, 1H, alkene-H), 6.50 (s, 1H, Ph-H), 6.56 (d, 1H, *J* = 9.68 Hz, Ph-H), 6.81 - 6.92 (m, 4H, Ph-H), 7.09 (s, 1H, alkene-H), 7.33 (d, *J* = 7.64 Hz, 1H, Ph-H), 7.42 (t, *J* = 8.32 Hz, 1H, Ph-H), 7.50 (d, *J* = 8.32 Hz, 1H, Ph-H), 7.67 – 7.73 (m, 2H, Ph-H), 7.78 (t, *J* = 7.48 Hz, 1H, Ph-H), 8.04 (d, *J* = 15.60 Hz, 1H, alkene-H), 8.28 (d, *J* = 7.80 Hz, 1H, Ph-H), 8.91 (d, *J* = 8.32 Hz, 1H, Ph-H). Mass spectrometry (ESI

positive ion mode for $[M + H]^+$): calcd for $[C_{42}H_{27}N_2O_6^+]$: 655.1869; found: 655.1873.

Synthesis of BI-IFC-ester. BI-IFC (30 mg, 0.044 mmol) was dissolved in acetic anhydride (20 mL), the system was under argon protection and then refluxed for 10 h. Acetic anhydride was removed by evaporation, and the residue was purified by silica gel chromatography (CH_2Cl_2/MeOH 100:1) to get the desired product BI-IFC-ester (10 mg, 0.014 mmol), a dark mazarine solid. Yield was 31%. ^{1}H-NMR (400 MHz, $CDCl_3$, ppm): δ 1.62 (t, 3H, -CH_2*CH*$_3$), 2.00 (m, 2H, -*CH*$_2$), 2.03 (s, 3H, -*CH*$_3$), 2.04 (s, 3H, -*CH*$_3$), 2.36 (s, 3H, -*CH*$_3$), 2.82 (m, 2H, -*CH*$_2$), 2.92 (m, 2H, -*CH*$_2$), 4.78 - 4.88 (m, 2H, -*CH*$_2$$CH_3$), 6.80 (d, J = 15.32 Hz, 1H, alkene-H), 6.83 (d, J = 8.76 Hz, 1H, Ph-H), 6.86 - 6.89 (m, 2H, Ph-H), 7.07 (d, J = 8.76 Hz, 1H, Ph-H), 7.17 (d, J = 7.36 Hz, 1H, Ph-H), 7.27 (s, 1H, alkene-H), 7.50 (s, 1H, Ph-H), 7.59 (t, J = 7.60 Hz, 1H, Ph-H), 7.68-7.76 (m, 4H, Ph-H), 8.02 (t, J = 9.20 Hz, 2H, Ph-H), 8.08 (d, J = 7.12 Hz, 1H, Ph-H), 8.22 (d, J = 8.48 Hz, 1H, Ph-H), 8.67 (d, J = 15.32 Hz, 1H, alkene-H). Mass spectrometry (ESI positive ion mode for $[M]^+$): calcd for $[C_{47}H_{38}NO_6^+]$: 712.2699; found: 712.2699.

Synthesis of IFC probes. The intermediate compound CPT-S-OH[4] was synthesized by the established procedures.

Synthesis of DCM-IFC-1. DCM-IFC (50 mg, 0.078 mmol) and DMAP (34 mg, 0.27 mmol) were dissolved in CH_2Cl_2 (15 mL) at 0 ºC, then acryloyl chloride (0.1 ml, mixed with 5 mL of CH_2Cl_2) was added dropwise and kept stirring at this temperature 30 min. Then the mixture was warmed to room temperature and stirred overnight. CH_2Cl_2 was removed by evaporation, and the residue was purified by silica gel chromatography (CH_2Cl_2/MeOH 100:1) to get the desired product DCM-IFC-1 (10 mg, 0.014 mmol), a dark purple solid. Yield was 18%. ^{1}H-NMR (400 MHz, $CDCl_3$, ppm): δ 1.91 (m, 2H, -*CH*$_2$), 2.52 (m, 2H, -*CH*$_2$), 2.68 (m, 2H, -*CH*$_2$), 6.08 (d, J = 10.48 Hz, 1H, alkene-H), 6.15 (d, J = 15.52 Hz, 1H, alkene-H), 6.34 (dd, J_1 = 10.48 Hz, J_2 = 17.32 Hz, 1H, alkene-H), 6.64 (d, J = 8.68 Hz, 1H, Ph-H), 6.65 (d, J = 17.32 Hz, 1H, alkene-H), 6.77 (s, 1H, alkene-H), 6.79 (d, J = 8.92 Hz, 1H, Ph-H), 6.83 (s, 1H, Ph-H), 6.84 (d, J = 1.8 Hz, 1H, Ph-H), 7.04 (s, 1H, alkene-H), 7.19 (d, J = 7.4 Hz, 1H, Ph-H), 7.25 (d, J = 1.8 Hz, 1H, Ph-H), 7.40 (t, J = 7.4 Hz, 1H, Ph-H), 7.52 (d, J = 7.68 Hz, 1H, Ph-H), 7.65-7.73 (m, 3H, Ph-H), 8.04 (d, 1H, J = 15.52 Hz, alkene-H), 8.07 (d, J = 6.80 Hz, 1H, Ph-H), 8.89 (d, J = 8.32 Hz, 1H, Ph-H). Mass spectrometry (ESI positive ion mode for $[M + H]^+$): calcd for $[C_{44}H_{27}N_2O_7^+]$: 695.1818; found: 695.1815.

Synthesis of DCM-IFC-2. DCM-IFC (50 mg, 0.078 mmol) and DMAP (34 mg, 0.27 mmol) were dissolved in CH_2Cl_2 (15 mL) at 0 ºC, then 2,4-Dinitrobenzenesulfonyl chloride (41 mg, 0.156 mmol, mixed with 5 mL of CH_2Cl_2) was added dropwise and kept stirring at this temperature 30 min. Then the mixture was warmed to room temperatur and stirred overnight. CH_2Cl_2 was removed by evaporation, and the residue was purified by silica gel chromatography (CH_2Cl_2/MeOH 100:1) to get the desired product DCM-IFC-2 (10 mg, 0.011 mmol), a dark purplish red solid. Yield was 14%. ^{1}H-NMR (400 MHz, DMSO-d_6, ppm): δ 1.80 (m, 2H, -*CH*$_2$), 2.67 (m, 2H, -*CH*$_2$), 6.75 (d, J = 15.96 Hz, 1H, alkene-H), 6.77 (d, J = 8.16 Hz, 1H, Ph-H), 6.93 - 6.99 (m, 3H), 7.11 (d, J = 8.92 Hz, 1H, Ph-H), 7.25 (s, 1H, alkene-H), 7.38 (d, J = 7.64 Hz, 1H, Ph-H), 7.55 - 7.59 (m, 2H), 7.77 (t, J = 7.28 Hz, 1H, Ph-H), 7.82 - 7.90 (m, 3H), 8.04 (d, 1H, J = 15.96 Hz, alkene-H), 8.07 (d, J = 7.88 Hz, 1H, Ph-H), 8.37 (d, J = 8.72 Hz, 1H, Ph-H), 8.63 (dd, J_1 = 8.72 Hz, J_2 = 2.28 Hz, 1H, Ph-H), 8.70 (d, J = 8.76 Hz, 1H, Ph-H), 9.14 (d, J = 2.28 Hz, 1H, Ph-H). Mass

spectrometry (ESI positive ion mode for $[M + H]^+$): calcd for $[C_{47}H_{27}N_4O_{12}S^+]$: 871.1346; found: 871.1343.

Synthesis of DCM-IFC-3. DCM-IFC (100 mg, 0.15 mmol), DMAP (39.5 mg, 0.30 mmol) and triphosgene (14 mg, 0.047 mmol) were dissolved in dry toluene (15 mL) under an argon atmosphere at room temperature. The resulting solution was refluxed under argon protection for 3 h. After removal of unreacted phosgene gas by flushing argon gas, a solution of CPT-S-OH (50 mg, 0.094 mmol) in $CHCl_3$ (10 mL) was added to the mixture and the reaction mixture was stirred overnight at room temperature. The solvent was removed by evaporation, and the residue was purified by silica gel chromatography (CH_2Cl_2/MeOH 100:1) to get the desired product DCM-IFC-3 (20 mg, 0.016 mmol), a dark purple solid. Yield was 11%. ^{1}H-NMR (400 MHz, $CDCl_3$, ppm): δ 1.01 (m, 3H, -$CH_2C\mathit{H_3}$), 1.90 (m, 2H, -$C\mathit{H_2}$), 2.11-2.25 (m, 2H, -$C\mathit{H_2}CH_3$), 2.51 (m, 2H, -$C\mathit{H_2}$), 2.68 (m, 2H, -$\mathit{CH_2}$) 2.98 - 3.01 (m, 4H, -$C\mathit{H_2}$-S-S-$C\mathit{H_2}$-), 4.36 - 4.50 (m, 4H, -$C\mathit{H_2}CH_2$-S-S-$CH_2C\mathit{H_2}$-), 5.27 (s, 1H, -N-CH), 5.28 (s, 1H, -N-CH), 5.38 (d, *J* = 17.28 Hz, 1H, -O-$C\mathit{H_2}$), 5.69 (d, *J* = 17.28 Hz, 1H, -O-$C\mathit{H_2}$), 6.64 (d, *J* = 8.60 Hz, 1H, Ph-H), 6.78 (d, *J* = 8.60 Hz, 2H, Ph-H), 6.80 - 6.88 (m, 2H, Ph-H), 7.04 (s, 1H, alkene-H), 7.18 (t, *J* = 6.76 Hz, 1H, Ph-H), 7.25 (t, *J* = 2.32 Hz, 1H, Ph-H), 7.35 (s, 1H, Ph-H), 7.41 (t, *J* = 7.94 Hz, 1H, Ph-H), 7.53 (d, *J* = 8.12 Hz, 1H, Ph-H), 7.65-7.71 (m, 5H, Ph-H), 7.84 (t, *J* = 7.20 Hz, 1H, Ph-H), 7.93 (dd, J_1 = 7.60 Hz, J_2 = 4.64 Hz, 1H, Ph-H), 8.02 (d, *J* = 3.60 Hz, 1H, Ph-H), 8.05-8.07 (m, 1H, Ph-H), 8.22 (d, *J* = 8.44 Hz, 1H, Ph-H), 8.40 (d, *J* = 4.64 Hz 1H, Ph-H), 8.89 (d, *J* = 7.40 Hz 1H, Ph-H). Mass spectrometry (ESI positive ion mode for $[M + Na]^+$): calcd for $[C_{67}H_{46}N_4O_{14}S_2Na^+]$: 1217.2350; found: 1217.2343.

Synthesis of Biotin-DCM-IFC. Alkyne-DCM-IFC (50 mg, 0.07 mmol) and Biotin-PEG_{15}-N_3 (108 mg, 0.18 mmol) were dissolved in dry DMF (2 mL), then CuI (20 mg, 0.108 mmol) was added under an argon atmosphere at room temperature. Then the reaction mixture was stirred overnight at room temperature. The solution was added to CH_2Cl_2 (20 mL) and washed with deionized H_2O (20 mL × 5). The organic layer was dried over anhydrous Na_2SO_4, filtered and concentrated by evaporation. The residue was purified by silica gel chromatography (CH_2Cl_2/MeOH 10:1) to get the desired product Biotin-DCM-IFC (30 mg, 0.028 mmol), a dark purple solid. Yield was 40%. ^{1}H-NMR (400 MHz, DMSO-d_6, ppm): δ 1.41 - 1.49 (m, 6H, -$\mathit{CH_2}$), 1.60 (m, 2H, -$\mathit{CH_2}$), 2.57 (m, 1H, -S*CH*), 2.79 - 2.84 (m, 3H, -S*CH*, -CH_2), 3.09 (m, 1H, -S*CH*), 3.50 (m, 60H, -O$\mathit{CH_2CH_2}$), 3.80 (m, 2H, -NCO$\mathit{CH_2}$), 4.00 (s, 1H, OC*H*), 4.12 (m, 2H, -O$C\mathit{H_2}$), 4.30 (m, 2H, -O$C\mathit{H_2}$), 4.51 (m, 2H, -N$C\mathit{H_2}$), 4.68 (s, 1H, NC*H*), 4.69 (s, 1H, NC*H*), 6.36 (s, 1H, -N*H*), 6.42 (s, 1H, -N*H*), 6.58 (s, 2H, Ph-H), 6.65 (d, *J* = 8.88 Hz, 1H, Ph-H), 6.72 (d, *J* = 15.56 Hz, 1H, alkene-H), 6.79 (s, 1H, Ph-H), 6.91 (s, 1H, alkene-H), 6.97 (d, *J* = 8.88 Hz, 1H, Ph-H), 7.25 (s, 1H, alkene-H), 7.33 (dd, J_1 = 7.52 Hz, J_2 = 4.84 Hz, 1H, Ph-H), 7.52 (t, *J* = 7.52 Hz, 1H, Ph-H), 7.75 (t, *J* = 4.84 Hz, 1H, Ph-H), 7.79 – 7.86 (m, 4H, Ph-H),7.96 (d, *J* = 15.56 Hz, 1H, alkene-H), 8.04 (d, *J* = 7.52 Hz, 1H, Ph-H), 8.08 (s, 1H, N*H*), 8.66 (d, *J* = 8.28 Hz, 1H, Ph-H), 10.26 (s, 1H, -O*H*). Mass spectrometry (ESI negative ion mode for $[M - H]^-$): calcd for $[C_{86}H_{105}N_8O_{24}S^-]$: 1665.6962; found: 1665.6958.

Synthesis of DCM-IFC-4. Biotin-DCM-IFC (30 mg, 0.028 mmol), DMAP (12 mg, 0.095 mmol) and triphosgene (4 mg, 0.014 mmol) were dissolved in dry toluene (15 mL) under an argon atmosphere at room temperature. The resulting solution was refluxed under argon protection for 3 h. After removal of unreacted phosgene gas by flushing

argon gas, a solution of CPT-S-OH (50 mg, 0.094 mmol) in $CHCl_3$ (10 mL) was added to the mixture and the reaction mixture was stirred overnight at room temperature. After removing the solvent under by evaporation, and the residue was purified by silica gel chromatography (CH_2Cl_2/MeOH 10:1) to get the desired product DCM-IFC-4 (10 mg, 0.0004 mmol), a dark purple solid. Yield was 14%. ^{1}H-NMR (400 MHz, $CDCl_3$, ppm): δ 1.01 (t, 3H, -CH_2*CH_3*),1.41 - 1.49 (m, 2H, -*CH_2*), 1.65 - 1.78 (m, 6H, -*CH_2*), 2.13 - 2.32 (m, 2H, -*CH_2*CH_3), 2.73 (m, 1H, -S*CH*), 2.78 - 2.94 (m, 3H, -S*CH*, -CH_2), 2.98 - 3.01 (m, 4H, *CH_2*CH_2-S-S-CH_2*CH_2*), 3.13 - 3.18 (m, 1H, -S*CH*), 3.38 - 3.46 (m, 4H, -NCO*CH_2*, -O*CH_2*), 3.56 (t, 2H, -N*CH_2*), 3.64 (m, 60H, -O*CH_2CH_2*), 4.06 (m, 1H, -OC*H*), 4.33 (m, 1H, -NC*H*), 4.41 (m, 1H, -NC*H*), 4.45 - 4.52 (m, 4H, -O*CH_2*), 4.56 (t, 2H, -O*CH_2*), 4.80 (s, 2H, -N*H*), 4.97 (s, 1H, N*H*), 5.28 (s, 2H, NC*H_2*), 5.38 (s, 1H, -O*CH*), 5.68 (m, 2H, -O*CH*, Ph-H), 6.66 (d, *J* = 8.76 Hz, 1H, Ph-H), 6.74 - 6.78 (m, 5H, Ph-H), 7.10 (d, *J* = 8.92 Hz, 1H, Ph-H), 7.19 (q, *J* = 11.84 Hz, 1H, Ph-H), 7.35 (s, 1H, Ph-H), 7.41 (t, *J* = 7.92 Hz, 1H, Ph-H), 7.53 (d, *J* = 8.36 Hz, 1H, Ph-H), 7.65 - 7.73 (m, 4H, Ph-H), 7.80 - 7.85 (m, 2H, Ph-H), 7.94 (t, *J* = 7.28 Hz, 1H, Ph-H), 8.02 (d, *J* = 15.88 Hz, 1H, alkene-H), 8.06 (d, *J* = 7.44 Hz, 1H, Ph-H), 8.22 (d, *J* = 8.48 Hz, 1H, Ph-H), 8.40 (d, *J* = 4.80 Hz 1H, Ph-H), 8.89 (d, *J* = 8.36 Hz 1H, Ph-H). Mass spectrometry (ESI positive ion mode for $[M + H]^+$): calcd for $[C_{112}H_{129}N_{10}O_{32}S_3^+]$: 2223.8047; found: 2223.8025.

In vitro cytotoxicity assay. The cell cytotoxicity of DCM-IFC-1, DCM-IFC-3 and DCM-IFC-4 to HeLa cells, A549 cells or QSG-7701 cells (normal cells) were measured by 3-(4, 5-dimethylthiazol-2-yl)-2, 5-diphenyltetrazolium bromide (MTT) assay. The cytotoxicity was evaluated by Cell Counting Kit-8 (Dojindo, Tokyo, Japan) according to the factory's instruction. Cells were plated in 96-well plates in 0.1 mL volume of DMEM or RPMI-1640 medium with 10 % FBS, at a density of 1×10^4 cells/well and added with desired concentrations of DCM-IFC-1, DCM-IFC-3 and DCM-IFC-4. After incubation for 24 h, absorbance was measured at 450 nm with a Tecan GENios Pro multifunction reader (Tecan Group Ltd., Maennedorf, Switzerland). Each concentration was measured in triplicate and used in three independent experiments. The relative cell viability was calculated by the equation: cell viability (%) = $(OD_{treated}/OD_{control}) \times 100\%$.

Supplementary References

1. Gogoi J, Gogoi P, & Boruah RC. One-Pot Stereoselective synthesis of (Z)-β-ketoenamides from β-Halo α,β-unsaturated aldehydes. *Eur. J. Org. Chem.* **2014,** 3483-3490 (2014).
2. Chen X, Ko SK, Kim MJ, Shin I, & Yoon J. A thiol-specific fluorescent probe and its application for bioimaging. *Chem. Commun.* **46,** 2751-2753 (2010).
3. Yang Z*, et al.* Folate-based near-infrared fluorescent theranostic gemcitabine delivery. *J. Am. Chem. Soc.* **135,** 11657-11662 (2013).
4. Ye M*, et al.* Dual-channel NIR activatable theranostic prodrug for *in vivo* spatiotemporal tracking thiol-triggered chemotherapy. *Chem. Sci.* **7,** 4958-4965 (2016).
5. Gu K*, et al.* Real-time tracking and in vivo visualization of beta-galactosidase activity in colorectal tumor with a ratiometric near-infrared fluorescent probe. *J. Am. Chem. Soc.* **138,** 5334-5340 (2016).
